# New Bioactive Polyketides from the Mangrove-Derived Fungus *Daldinia eschscholzii* HJX1P2

**DOI:** 10.3390/md23060238

**Published:** 2025-05-30

**Authors:** Miao Yu, Yikang Qiu, Shiji Chen, Jueying Shi, Xiu Gong, Jiayi Feng, Fangru Lin, Weinv Zeng, Wenyuan Kang, Caijuan Zheng, Guolei Huang

**Affiliations:** 1 Key Laboratory of Tropical Medicinal Resource Chemistry of Ministry of Education, College of Chemistry and Chemical Engineering, Hainan Normal University, Haikou 571158, China; yumiaonpc@126.com (M.Y.); qyk7747226@sina.com (Y.Q.); chenshijinpc@126.com (S.C.); jueying202406@163.com (J.S.); 15228548005@163.com (X.G.); 19308057394@163.com (J.F.); fl326688@163.com (F.L.); kangsgo@hainnu.edu.cn (W.K.); 2 Key Laboratory of Tropical Medicinal Plant Chemistry of Hainan Province, Haikou 571158, China; 3 International Center for Aging and Cancer, Hainan Medical University, Haikou 571158, China; hy0322027@muhn.edu.cn

**Keywords:** naphthalene–chroman dimers, *Daldinia eschscholzii*, anti-inflammatory activity, antioxidant activity, anti-bacterial activity

## Abstract

Three new naphthalene–chroman dimer derivatives, daldinaphchromes A–C (**1**–**3**), two new chroman derivatives, daldichromes A (**5**) and B (**6**), along with five known compounds (**4**, **7**–**10**) were isolated from the mangrove-derived fungus *Daldinia eschscholzii* HJX1P2. Their structures and stereochemistries were elucidated through detailed NMR and MS analyses, calculated electronic circular dichroism, and comparison with previously reported data. Compound **1** demonstrated inhibitory effects on nitric oxide (NO) production in LPS-induced RAW 264.7 cells, with an IC_50_ value of 62.9 µM, and more effectively suppressed the expression of interleukin (IL)-6 than dexamethasone. A further mechanistic study suggested that **1** could prohibit the expression of iNOS in RAW 264.7 cells, and the molecular docking study suggested a possible interaction between **1** and the iNOS protein. Compounds **7** and **8** exhibited moderate to potent DPPH radical scavenging activity, with IC_50_ values of 117.4 and 46.2 µM, respectively, compared with the positive control ascorbic acid (IC_50_ = 45.6 µM). Compounds **4** and **10** showed ABTS^+^ radical scavenging activity, with IC_50_ values of 66.6 and 33.2 µM, respectively, which were equal to or lower than that of the positive control vitamin C (IC_50_ = 59.7 µM). Compounds **1**–**3**, **7**, and **9** showed antibacterial activity against three *Staphylococcus aureus* strains, with MIC values of 74.4–390.6 μM.

## 1. Introduction

Mangroves, as highly productive ecosystems widely distributed along tropical and subtropical coasts, harbor rich microbial resources, and have a significant role in altering microbial communities and nutrient cycles [1]. Mangrove fungi are considered to be an underexplored source of novel bioactive compounds, which meet the need of confronting new multidrug-resistant pathogens [2,3,4,5,6,7]. Among them, the genus *Daldinia* has shown great potential in medicine, environmental protection, and agriculture, emerging as a promising source for bioactive compound discovery. From January 1995 to June 2024, 280 metabolites, including 196 new compounds and 112 bioactive compounds, were obtained from the genus *Daldinia* [7,8,9]. *Daldinia* sp. isolated from mangroves produce a wide range of biologically active secondary metabolites, such as polyketides, alkaloids, polyphenols, and terpenoids [10,11,12,13].

**Figure 1:**
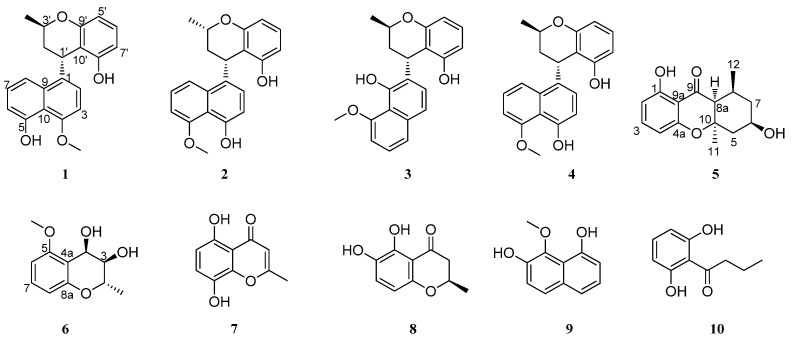
Structures of compounds **1**–**10**.

During our ongoing investigations into the fungi inhabiting mangrove environments [14,15,16,17,18,19], the fermentation of the fungus *Daldinia eschscholzii* HJX1P2, collected from the Hainan Mangrove Conservation Area, displayed significant anti-inflammatory activity. The subsequent purification led to the discovery of three new naphthalene–chroman dimer derivatives, daldinaphchromes A–C (**1**–**3**), two new chroman derivatives, daldichromes A (**5**) and B (**6**), along with five known compounds, nodulisporin D (**4**) [20], 8-ethyl-7-hydroxy-2,3-dimethyl-4-oxo-chromene-5-carboxylic acid (**7**) [21], (2*S*)-2,3-dihydro-5,6-dihydroxy-2-methyl-4*H*-1-benzopyran-4-one (**8**) [22], 8-methoxynaphthalene-1,7-diol (**9**) [23], and 1-(2,6-dihydroxy-phenyl)butan-1-one (**10**) [24] (Figure 1). Herein, we report the isolation, structure elucidation, and bioactivity of these compounds.

## 2. Results and Discussion

### 2.1. Structural Determination

**Figure 2:**
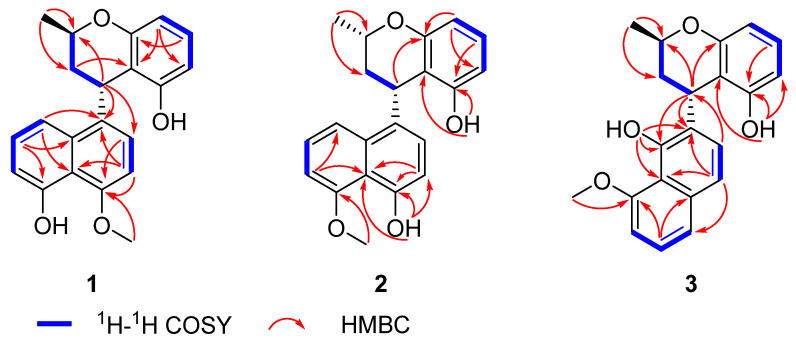
Key HMBC and ^1^H-^1^H COSY correlations of compounds **1**–**3**.

Compound **1** was isolated as a yellow gum. Its molecular formula was determined as C_21_H_20_O_4_ based on an HR-ESI-MS ion peak at *m*/*z* 335.1291 [M-H]^−^, appropriate for twelve degrees of unsaturation. The ^1^H NMR spectroscopic data (Table 1), combined with the HSQC spectrum of **1**, showed two hydroxyl groups at *δ*_H_ 9.36 (1H, s, 5-OH) and 9.62 (1H, s, 8′-OH); eight aromatic protons at *δ*_H_ 6.60 (1H, d, *J* = 8.0 Hz, H-2), 6.80 (1H, d, *J* = 8.0 Hz, H-3), 6.84 (1H, d, *J* = 7.8 Hz, H-6), 7.45 (1H, t, *J* = 7.8 Hz, H-7), 7.70 (1H, d, *J* = 7.8 Hz, H-8), 6.35 (1H, d, *J* = 8.4 Hz, H-5′), 6.97 (1H, t, *J* = 8.4 Hz, H-6′), and 6.35 (1H, d, *J* = 8.4 Hz, H-7′); one oxygenated methine signal at *δ*_H_ 3.87 (1H, m, H-3′); one methoxyl group at *δ*_H_ 3.96 (3H, s, 4-OMe); one methine proton at *δ*_H_ 4.80 (1H, d, *J* = 5.6 Hz, H-1′); one methylene group at *δ*_H_ 1.95 (1H, ddd, *J* = 13.6, 11.6, 5.6 Hz, H-2′*β*) and 1.86 (1H, d, *J* = 13.6 Hz, H-2′*α*); and one methyl group at *δ*_H_ 1.18 (3H, d, *J* = 6.2 Hz, 3′-Me). The ^13^C NMR data (Table 2) and DEPT spectrum of **1** indicated the presence of 21 carbon signals, including sixteen aromatic carbons at *δ*_C_ 134.3 (C-1), 125.5 (C-2), 103.5 (C-3), 154.4 (C-4), 155.0 (C-5), 110.0 (C-6), 127.6 (C-7), 114.2 (C-8), 133.3 (C-9), 114.8 (C-10), 106.8 (C-5′), 127.7 (C-6′), 106.5 (C-7′), 155.7 (C-8′), 156.4 (C-9′), and 110.6 (C-10′); one oxygenated methine carbon at *δ*_C_ 66.8 (C-3′); one methoxy carbon at *δ*_C_ 56.1 (4-OMe); one methylene carbon at *δ*_C_ 35.0 (C-2′); one methine carbon at *δ*_C_ 31.3 (C-1′); and one methyl carbon at *δ*_C_ 21.0 (3′-Me). These data suggest that **1** was similar nodulisporin D (**4**) [20]. The major difference involved the displacement of the methoxy and hydroxyl groups compared with those of **4**, which was deduced by the correlations of H-2 and H-3 with C-4 and 4-OMe with C-4, indicating that the methoxy group was attached to C-4 in **1** rather than to C-1 in **4**, as shown in the HMBC spectrum (Figure 1). The whole structure of **1** was further confirmed according to the ^1^H-^1^H COSY and HMBC spectra. The ^1^H-^1^H COSY of **1** allowed for the identification of four fragments, –CH(1′)–CH_2_(2′)–CH(3′)–CH_3_, –CH(5′)–CH(6′)–CH(7′)–, –CH(2)–CH(3)–, and –CH(6)–CH(7)–CH(8)–. The HMBC correlations of H-3 with C-1/C-10, H-7 with C-5/C-9, H-8 with C-1, H-5′ with C-7′/C-10′, H-1′ with C-3′, 3′-Me with C-2′/C-3′, and 4-OMe with C-4 confirmed the presence of naphthalene and chroman units (Figure 2). The connection between the chroman and naphthalene moieties within the structure was established by correlations of H-1′ with C-1/C-2 in the HMBC spectrum, further confirming the planar structure of **1**.

**Figure 3:**
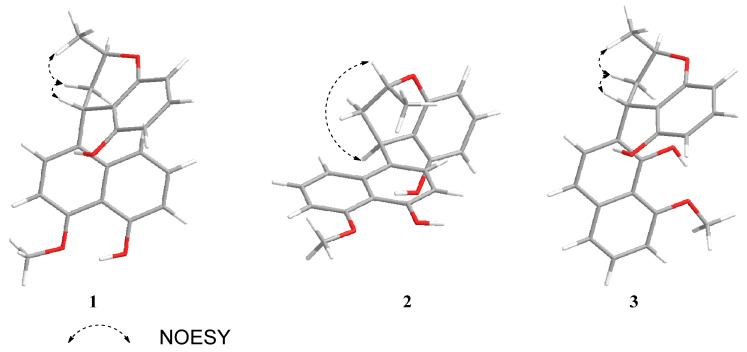
Key NOESY correlations of compounds **1**–**3**.

**Figure 4:**
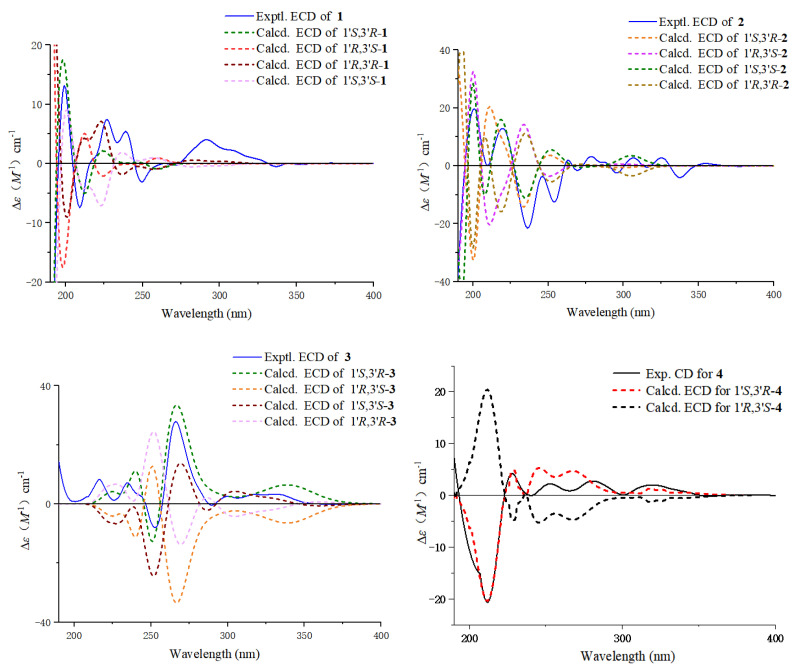
Experimental and calculated ECD spectra of compounds **1**–**4**. The green line corresponds to the optimal fitting configuration of compounds **1**–**3**. The relative configuration of compound **4** had been determined in the literature; therefore, only two configurations were computed, with the red line corresponding to the optimal fitting configuration.

The relative configurations of C-1′ and C-3′ were deduced from the NOESY correlations between H-1′ and H-2′*β*, as well as between H-2′*β* and 3′-Me, combined with the coupling constants of *J*_1′,2′*β*_ = 5.8 Hz and *J*_2′*β*,3′*α*_ = 11.6 Hz, indicating the H-1′*β* and H-3′*α* configurations (Figure 3). The absolute configuration of **1** was determined as 1′*S*,3′*R* according to the quantum chemical ECD calculation (Figure 4) and named as daldinaphchrom A.

Compound **2** was also obtained as a yellow gum, with a molecular formula of C_21_H_20_O_4_ based on HR-ESI-MS data at *m*/*z* 359.1258 [M + Na]^+^, indicating 12 degrees of unsaturation. The ^1^H NMR data (Table 1), combined with the HSQC spectrum of **2**, showed two hydroxyl groups at 4-OH (*δ*_H_ 9.34) and 8′-OH (*δ*_H_ 8.90); eight aromatic protons at *δ*_H_ 7.77 (1H, m, H-8), 7.37 (1H, m, H-7), 6.94 (1H, m, H-6), 6.93 (1H, m, H-6′), 6.90 (1H, m, H-2), 6.62 (1H, d, *J* = 7.8 Hz, H-3), 6.35 (1H, d, *J* = 7.4 Hz, H-5′), and 6.25 (1H, d, *J* = 7.4 Hz, H-7′); one oxygenated methine signal at *δ*_H_ 4.10 (1H, m, H-3′); one methoxyl group at *δ*_H_ 4.02 (3H, s, 5-OMe); one methine proton at *δ*_H_ 4.79 (1H, s, H-1′); one methylene group at *δ*_H_ 2.48 (1H, m, H-2′*α*) and 1.67 (1H, m, H-2′*β*); and one methyl group at *δ*_H_ 1.24 (3H, d, *J* = 6.2 Hz, 3′-Me). Furthermore, the ^13^C NMR data (Table 2) and DEPT spectrum of **2** indicated the presence of 21 carbon signals, including sixteen aromatic carbons, one oxygenated methine carbon, one methoxy carbon, one methylene carbon, one methine carbon, and one methyl carbon. These ^1^H and ^13^C NMR spectroscopic data suggested that the plane structure of **2** was the same as that of nodulisporin D (**4**) [20]; these results were also confirmed by the COSY and HMBC correlations (Figure 2). The clear difference was the configurations of C-1′ and C-3′. The NOESY correlation of H-1′ and H-3′ indicated that H-1′ and H-3′ exhibited the same configuration in **2** (Figure 3). The absolute configuration of compound **2** was determined to be 1′*S*,3′*S*, while that of compound **4** was determined to be 1′*S*,3′*R* based on ECD calculations (Figure 4). Thus, the structure of compound **2** was identified as that in Figure 1 and named as daldinaphchrome B.

Compound **3** was also isolated as a yellow gum and had the molecular formula C_21_H_20_O_4_ based on the prominent signal at *m*/*z* 359.1259 [M + Na]^+^ in the HR-ESI-MS spectrum. The ^1^H and ^13^C NMR spectroscopic data (Table 1 and Table 2) indicated that the planar structure of **3** was identical to that of nodulisporin E [20]. However, the optical rotation value of nodulisporin E was [*α*${]}_{D}^{25}$ = –4.5 (*c* = 0.02, CHCl_3_), and that of compound **3** was [*α*${]}_{D}^{25}$ = +19.5 (*c* = 0.2, MeOH), indicating the differences in their configurations. The relative configuration of **3** was deduced by NOESY correlations, as was that of nodulisporin E (Figure 3). The absolute configuration of compound **3** was determined to be 1′*S*,3′*R* based on quantum chemical ECD calculations (Figure 4). Compound **3** was named daldinaphchrom C.

**Figure 5:**
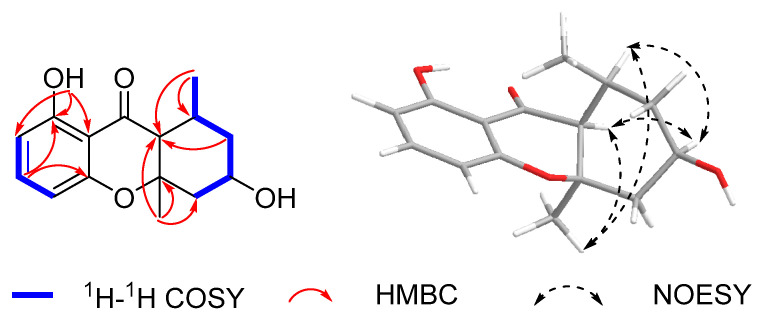
Key HMBC, ^1^H-^1^H COSY, and NOESY correlations of compound **5**.

**Figure 6:**
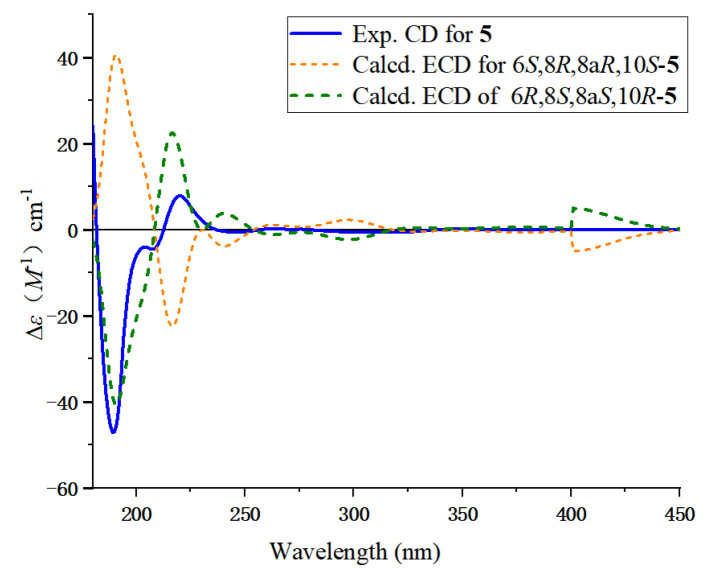
Experimental and calculated ECD spectra of compound **5** and CD spectrum of monodictysin C.

Compound **5** was obtained as a white powder, with a molecular formula of C_15_H_18_O_4_ based on the HR-ESI-MS data at *m*/*z* 285.1098 [M + Na]^+^, indicating seven degrees of unsaturation. The ^1^H NMR data (Table 3), combined with the HSQC spectrum of **5** showed three aromatic protons at *δ*_H_ 6.47 (1H, d, *J* = 8.4 Hz, H-2), 7.34 (1H, t, *J* = 8.4 Hz, H-3), and 6.36 (1H, d, *J* = 8.4 Hz, H-4); one oxygenated methine proton at *δ*_H_ 4.13 (2H, m, H-6); two methine protons at *δ*_H_ 1.86 (1H, m, H-8) and 1.85 (1H, br s, H-8a); two methylene groups at *δ*_H_ 2.41 (1H, m, H-5*α*), 1.39 (1H, m, H-5*β*), 2.06 (1H, d, *J* = 12.0 Hz, H-7*α*), and 1.09 (1H, m, H-7*β*); and two methyl groups at *δ*_H_ 1.35 (3H, s, H-11) and 0.98 (3H, d, *J* = 6.2 Hz, H-12). The ^13^C NMR data (Table 3) contained 15 resonances resulting from two methyl, two methylene, five methine, and six quaternary carbons, including a carbonyl group (*δ*_C_ 201.3, C-9), suggested a chromone skeleton that was further verified by the HMBC correlations. Additionally, there was one oxygenated methine group at *δ*_C_ 66.0 (C-6), two methylene carbon signals at *δ*_C_ 46.1 (C-5) and 42.6 (C-7), one methine group at *δ*_C_ 30.6 (C-8), and two methyl groups at *δ*_C_ 24.4 (C-11) and 19.5 (C-12). Combined with the unsaturation display, this indicates the presence of another ring in the structure. The ^1^H-^1^H COSY correlations of H-5*α*/H-6/H-7*β*/H-8a/H-12 suggested the presence of a six-membered ring fragment in the structure. The HMBC correlations of H-12 with C-8/C-8a and H-11 with C-5/C-10 indicated the presence of two methyl groups connected at C-8 and C-10, establishing the planar structure of compound **5** (Figure 5). The NOESY correlations of H-6/H-8 and H-6/H-8a, H-11/H-5*α* and H-5*α*/H-6, and 12-Me/ H-5*β* confirmed the relative configurations of **5**. The absolute configuration of **5** was determined to be 6*R*,8*S*,8a*S*,10*R* based on comparing its CD spectrum with that of monodictysin C [25]. The observation of a strong positive cotton effect at 218 nm (+8.93) confirmed that it shared the same absolute configuration as that of the reported structure. The configuration of **5** was also confirmed by the ECD calculations. (Figure 6). Thus, compound **5** was named as daldichrome A (**5**).

**Figure 7:**
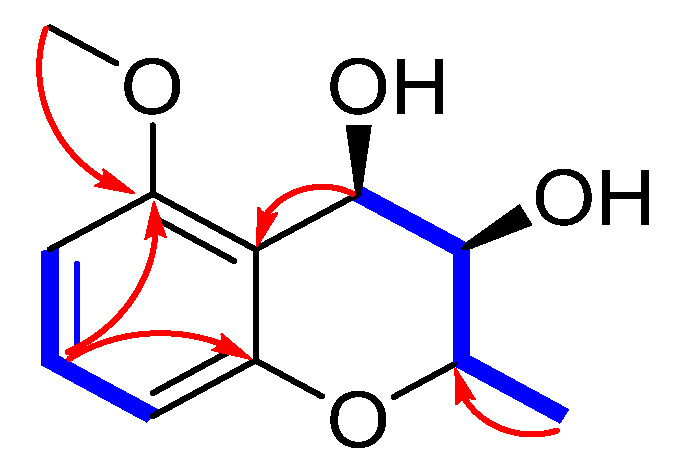
Key HMBC and ^1^H-^1^H COSY correlations of compound **6**.

**Figure 8:**
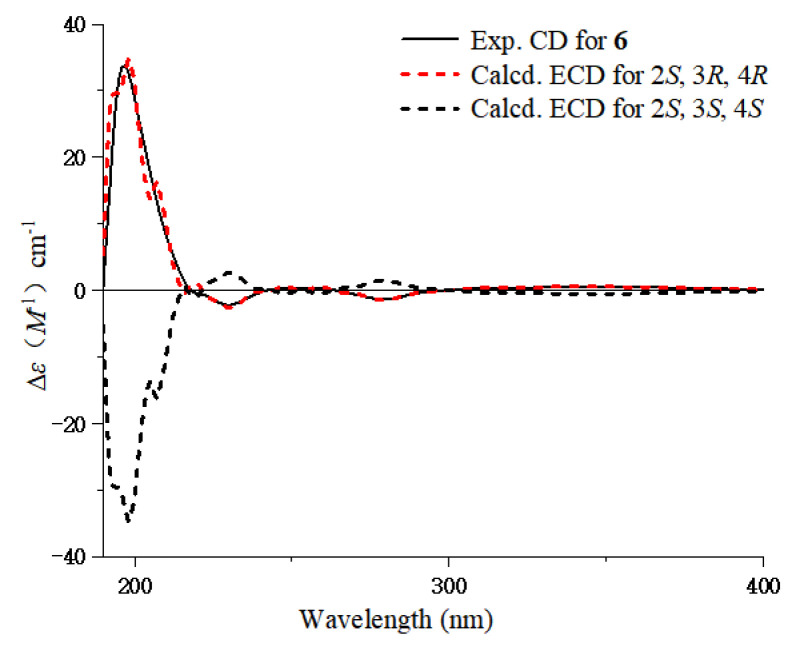
Experimental and calculated ECD spectra of compound **6**.

Compound **6** was obtained as a yellow gum, with a molecular formula of C_11_H_14_O_4_ based on HR-ESI-MS data at *m*/*z* 233.0780 [M + Na]^+^, indicating five degrees of unsaturation. The ^1^H NMR spectroscopic data (Table 3), combined with the HSQC spectrum of **6**, showed three aromatic protons at *δ*_H_ 7.17 (1H, dd, *J* = 8.4, 8.4 Hz, H-7), 6.51 (1H, d, *J* = 8.4 Hz, H-8), and 6.47 (1H, d, *J* = 8.4 Hz, H-6); three oxygenated methine protons at *δ*_H_ 4.97 (1H, d, *J* = 4.2 Hz, H-4), 4.06 (1H, dq, *J* = 9.6, 6.0 Hz, H-2), and 3.61 (1H, br s, H-3); one methyl group at *δ*_H_ 1.49 (3H, d, *J* = 6.0 Hz, 2-Me); and one methoxy group at *δ*_H_ 3.88 (3H, s, 5-OMe). In addition, 11 carbon signals were detected in the ^13^C NMR spectrum, including six aromatic carbons at *δ*_C_ 158.9, 155.2, 130.3, 112.2, 109.9, and 102.4; three oxygenated methine carbons at 71.2, 71.1, and 61.5; one methoxy group at 55.8 (5-OMe); and one methyl group at 17.6 (2-Me). The ^1^H-^1^H COSY correlations of H-6/H-7/H-8 and 2-Me/H-2/H-3/H-4 suggested two fragments. Combining the HMBC correlations of H-7/C-5 and C-8a, H-5/5-OMe established the planar structure of compound **6** (Figure 7). The large coupling constant (*J*_2,3_ = 9.6 Hz) and the small coupling constant (*J*_3,4_ = 4.2 Hz) in the ^1^H NMR spectrum indicate the trans configuration of H-2 and H-3, and the cis configuration of the vicinal diol of H-3 and H-4. The absolute configuration of compound **6** was determined to be 2*S*,3*R*,4*R* based on quantum chemical ECD calculations (Figure 8), and it was named as daldichrome B (**6**).

The other compounds were identified as 8-ethyl-7-hydroxy-2,3-dimethyl-4-oxo-chromene-5-carboxylic acid (**7**), (2*S*)-2,3-dihydro-5,6-dihydroxy-2-methyl-4*H*-1-benzopyran-4-one (**8**), 8-methoxynaphthalene-1,7-diol (**9**), and 1-(2,6-dihydroxy-phenyl)butan-1-one (**10**). The ^1^H and ^13^C NMR data of the known compounds **4**, **7**–**10** are in Supplementary Materials.

### 2.2. Bioactivity Assay

**Figure 9:**
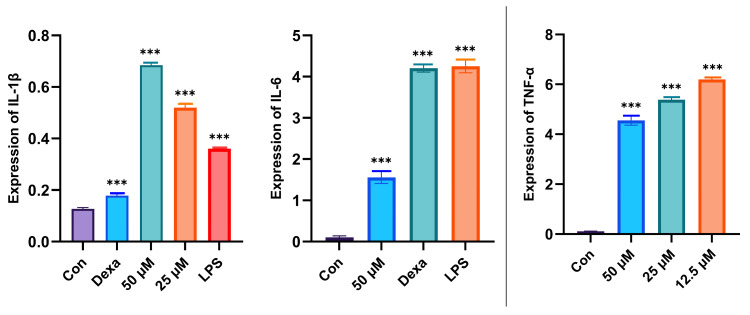
The expression levels of IL-1*β*, IL-6, and TNF-*α* of compound **1**. Data rendered are the mean ± SD, n = 3. In comparison with the control, *** *p* < 0.001. Con: Control, Dexa: Dexamethasone.

**Figure 10:**
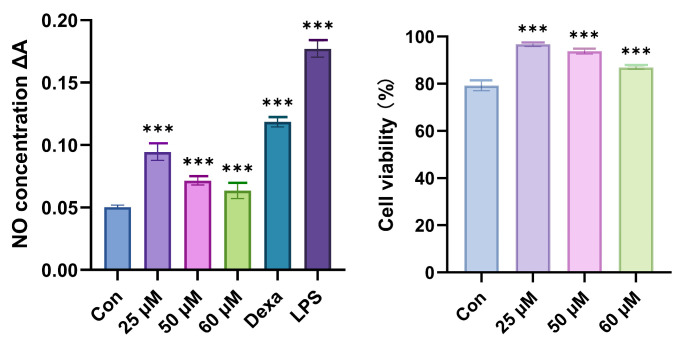
Influences of compound **1** on NO production for LPS-induced RAW 264.7 cells and cell viability. Data rendered are the mean ± SD, n = 3. In comparison to the control, *** *p* < 0.001. Con: Control, Dexa: Dexamethasone.

Compounds **1**–**4** were evaluated for their inhibitory activities against LPS-induced nitric oxide (NO) production in RAW 264.7 mouse macrophages, with compounds **2**–**4** exhibiting >50% inhibition at a concentration of 100 μM. Only compound **1** demonstrated moderate inhibition of NO accumulation induced by LPS on RAW 264.7 cells in a dose-dependent manner at concentrations of 25, 50, and 60 μM. Compound **1** exhibited anti-inflammatory activity with an IC_50_ value of 62.9 µM, better than that of the positive control dexamethasone, with an IC_50_ value of 136.8 µM. Additionally, compound **1** significantly inhibited IL-6 production in vitro, surpassing the inhibitory activity of dexamethasone (Figure 9). Compound **1** did not show toxicity at concentrations of 25, 50, and 60 µM (Figure 10).

**Figure 11:**
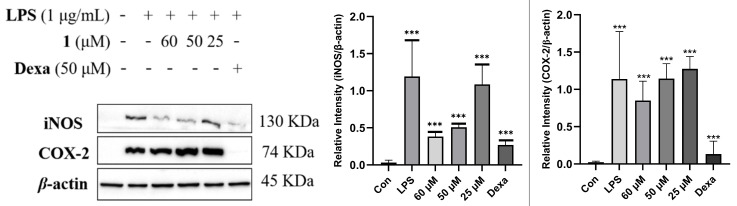
Influences of compound **1** on iNOS, COX-2, and *β*-actin protein expression were detected using western blotting. Data rendered are the mean ± SD, n = 3. In comparison with the control, *** *p* < 0.001. Con: Control, Dexa: Dexamethasone.

The inhibitory effects of inflammation-related iNOS and COX-2 for **1** were also measured using western blotting. As a result, the protein expression of iNOS was apparently down-regulated after the treatment of **1** with different concentrations (60.0, 50.0, and 25.0 µM) in a dose-dependent manner. Compound **1** exhibited no significant effect on the expression of the COX-2 protein (Figure 11).

**Figure 12:**
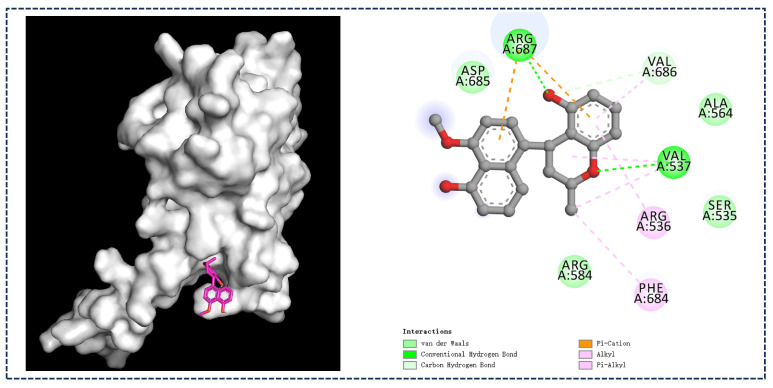
Binding positions between **1** and iNOS protein.

In order to study the possible recognition between the iNOS protein and compound **1**, AutoDock Vina was applied, as depicted in Figure 12 [26]; **1** exhibited an optimal fit within the binding pocket of iNOS protein, which was delineated by the side chains of five amino acid residues, Arg 687, Val 686, Val 537, Arg 536, and Phe 684, which may be critical for the observed inhibitory activity.

Compounds **7** and **8** exhibited DPPH radical scavenging activity, with IC_50_ values of 117.4 µM and 46.2 µM, respectively. Compound **8** demonstrated activity compared with the positive control, ascorbic acid, which had an IC_50_ value of 45.6 µM. Compounds **4** and **10** showed ABTS^+^ radical scavenging activity, with IC_50_ values of 66.6 µM and 33.2 µM, respectively. Notably, compound **10** demonstrated better efficacy than the positive control (vitamin C, IC_50_ = 59.7 µM).

Compounds **1**–**3**, **7,** and **9** showed weak antibacterial activity against *Staphylococcus aureus* (ATCC 29213) and two Methicillin-resistant *S. aureus* (MRSA) (ATCC 700699 and 43300), with MIC values of 74.4–390.6 μM, while the positive control vanconmycin exhibited an IC_50_ value of 1.0 µM (Table 4).

## 3. Materials and Methods

### 3.1. General Experimental Procedures

A Modular Circular Polarimeter 500 (Anton Paar, Graz, Austria) was used to detect optical rotation. A JASCO J-715 spectrophotometer (JASCO, Tokyo, Japan) was used to measure ECD spectra. The 1D (^1^H and ^13^C) and 2D (HSQC, HMBC, COSY, and NOESY) NMR spectra were measured on an NMR spectrometer (JEOL, 600 MHz, Tokyo, Japan) and a Bruker AV-400 (Bruker Corporation, Zurich, Switzerland) instrument with TMS as the internal standard. Chemical shifts (*δ*, ppm) were referenced using the residual solvent signals of DMSO-*d*_6_ (*δ*_H_ 2.50; *δ*_C_ 39.5), CD_3_OD-*d*_4_ (*δ*_H_ 3.31; *δ*_C_ 49.0), and CDCl_3_ (*δ*_H_ 7.26; *δ*_C_ 77.2). ESI-MS and HR-ESI-MS spectra were obtained on a Bruker Daltonics Apex-Ultra 7.0 T (Bruker Corporation, Billerica, MA, USA) and a Q-TOF Ultima Global GAA076 LC mass spectrometer. For semipreparative HPLC, an Agilent 1100 prep-HPLC system with a Waters C18 semipreparative column (9.4 × 250 mm, 7 μm) was used. Sephadex LH-20 (Pharmacia Co. Ltd., Sandwich, UK) and silica gel (200–300 and 300–400 mesh, Qingdao Marine Chemical Factory, Qingdao, China) were used for column chromatography (CC). Silica gel (GF254) for TLC were supplied by the Qingdao Marine Chemical Factory in China. All solvents used were of analytical grade (Guangzhou, China).

### 3.2. Fungal Material

The fungal strain HJX1P2 was collected from the Hainan Mangrove Conservation Area, Haikou. It was stored in the Key Laboratory of Tropical Medicinal Resource Chemistry of the Ministry of Education, College of Chemistry and Chemical Engineering, Hainan Normal University, Haikou, China. The strain was designated *Daldinia eschscholzii* HJX1P2 based on BLAST analysis of the ITS sequence (Supporting Information). Finally, the sequence was deposited in GenBank with the accession number OQ683818.

### 3.3. Fermentation and Extraction

The fungal strain *Daldinia eschscholzii* HJX1P2 was statically cultivated in potato dextrose broth PDB medium (26.0 g/1 L H_2_O, Potato extract 6.0 g, Glucose 20.0 g), followed by culture in 200 mL of PDB medium (0.3% sea salt) in 1 L Erlenmeyer flasks at 28 °C for 3 days on a rotary shaker at 180 rpm. A large-scale fermentation was then conducted at 26 °C for 28 days using a rice medium (50 g rice, 0.3% sea salt, and 50 mL H_2_O) in 1 L flasks (200 flasks total) under static conditions. The entire fermented culture was extracted with ethyl acetate three times, yielding a brown extract weighing 60.0 g.

### 3.4. Isolation and Purification

The extract was evaporated to dryness under a vacuum to obtain the organic extract (60.0 g), which was separated in twelve fractions (Fr.1−Fr.12) by silica gel column chromatography (CC) eluting with a petroleum ether (PE)/EtOAc gradient system from 0:1 to 1:0. Fraction Fr.1 was analyzed using TLC and ^1^H NMR to reveal mainly fatty acids. Fraction Fr.2 (4.1 g) was subjected to CC over octadecylsilyl (ODS) eluting with a MeOH/H_2_O gradient (30–100%) to yield six subfractions (SFr.2-1–SFr.2-6). Subfraction SFr.2-4 (0.85 g) was separated by CC on Sephadex LH-20 eluting with PE/CHCl_3_/MeOH (2:1:1, *v*/*v*/*v*) to yield three subfractions (SFr.2-4-1 to SFr.2-4-3), and then further purified by semipreparative HPLC (MeOH/H_2_O, 65:35, 2 mL/min) to give **1** (13.5 mg, *t*_R_ = 37.1 min), **4** (14.1 mg, *t*_R_ = 32.3 min), and **8** (4.4 mg, *t*_R_ = 15.5 min). Subfraction SFr.2-5 (0.17 g) was purified by semipreparative HPLC (MeOH/H_2_O, 70:30, 2 mL/min) to give **9** (6.5 mg, *t*_R_ = 25.3 min). Fraction Fr.4 (3.9 g) was subjected to CC over ODS eluting with a MeOH/H_2_O gradient (30–100%) to yield four subfractions (SFr.4-1−SFr.4-4). Subfraction SFr.4-2 (0.56 g) was separated by CC on Sephadex LH-20 eluting with PE/CHCl_3_/MeOH (2:1:1, *v*/*v*/*v*) to yield four subfractions (SFr.4-2-1 to SFr.4-2-4), and then further purified by semipreparative HPLC (MeOH/H_2_O, 65:35, 2 mL/min) to give **2** (8.4 mg, *t*_R_ = 27.5 min), **3** (10.5 mg, *t*_R_ = 33.5 min), **7** (2.3 mg, *t*_R_ = 35.8 min), and **10** (2.9 mg, *t*_R_ = 15.0 min). Fraction Fr.7 (1.9 g) was subjected by silica gel CC with a PE/EtOAc gradient system from 0:1 to 1:0 to yield seven subfractions (SFr.7-1 to SFr.7-7). Subfraction SFr.7-7 (0.60 g) was separated on Sephadex LH-20 eluting with PE/CHCl_3_/MeOH (2:1:1, *v*/*v*/*v*) to yield three subfractions (SFr.7-7-1 to SFr.7-7-3), and then further purified by semipreparative HPLC (MeOH/H_2_O, 30:70, 2 mL/min) to give **5** (1.5 mg, *t*_R_ = 14.5 min) and **6** (1.4 mg, *t*_R_ = 20.9 min). The HPLC detection wavelengths were 254, 210, 230, and 280 nm.

### 3.5. Spectroscopic Data of Compounds

Daldinaphchrome A (**1**): yellow oil; [*α*${]}_{D}^{25}$ −161.0 (*c* 0.2, CH_3_OH); UV (MeOH) *λ*_max_ (log *ε*) = 201, 203, and 213; IR (KBr) *ν*_max_ 3447, 1594, 1352, 1020, and 518 cm^−1^; ECD (2.98 mM, MeOH) *λ*max (∆*ε*) 198 (+17.51), 213 (−4.94), and 224 (+2.12); ^1^H and ^13^C NMR data, see Table 1 and Table 2; HRESIMS *m*/*z* 335.1291 [M-H]^−^ (calcd for C_21_H_20_O_4_Na^+^, 335.1289).

Daldinaphchrome B (**2**): brown oil; [*α*${]}_{D}^{25}$ +18.0 (*c* 0.2, CH_3_OH); UV (MeOH) *λ*_max_ (log *ε*) 214, 257, and 331; IR (KBr) *ν*_max_ 3462, 1596, 1353, and 527 cm^−1^; ECD (2.98 mM, MeOH) *λ*max (∆*ε*) 200 (+28.40), 207 (−9.56), 219 (+15.86), and 234 (−11.06); ^1^H and ^13^C NMR data, see Table 1 and Table 2; HRESIMS *m*/*z* 359.1258 [M + Na]^+^ (calcd for C_21_H_20_O_4_ Na ^+^, 359.1254).

Daldinaphchrome C (**3**): yellow oil; [*α*${]}_{D}^{25}$ +19.5 (*c* 0.2, CH_3_OH); UV (MeOH) *λ*_max_ (log *ε*) 211, 257, and 327; IR (KBr) ν_max_ 3450, 2829, 1596, 1354, and 517 cm^−1^; ECD (2.98 mM, MeOH) *λ*max (∆*ε*) 239 (+10.95), 251 (−12.68), and 268 (+33.07); ^1^H and ^13^C NMR data, see Table 1 and Table 2; HRESIMS *m*/*z* 359.1254 [M + Na]^+^ (calcd for C_21_H_20_O_4_Na^+^, 359.1259).

Daldichrome A (**5**): colorless oil; [*α*${]}_{D}^{25}$ +161.0 (*c* 0.1, CH_3_OH); UV (MeOH) *λ*_max_ (log *ε*) 199, 219, 248, and 278; IR (KBr) ν_max_ 3454, 2830, 1597, 1356, and 518 cm^−1^; ECD (3.82 mM, MeOH) *λ*max (∆*ε*) 190 (−40.32), 216 (+22.35), and 241 (+3.80); ^1^H and ^13^C NMR data, see Table 3; HRESIMS *m*/*z* 285.1098 [M + Na]^+^ (calcd for C_15_H_18_O_4_Na^+^, 285.1097).

Daldichrome B (**6**): yellow solid; [*α*${]}_{D}^{25}$ +32.0 (*c* 0.1, CH_3_OH); UV (MeOH) *λ*_max_ (log *ε*) 200, 215, 253, and 278; IR (KBr) ν_max_ 3455, 11595, 1353, and 517 cm^−1^; ECD (4.76 mM, MeOH) *λ*max (∆*ε*) 205 (+14.04), 211 (−5.66), 217 (+16.80), and 222 (−7.25); ^1^H and ^13^C NMR data see Table 3; HRESIMS *m*/*z* 233.0780 [M + Na]^+^ (calcd for C_11_H_14_O_4_Na^+^, 233.0784).

### 3.6. ECD Computation Section

Geometries were optimized at the level of B3LYP/6-31G(d) using density functional theory (DFT) calculations. The optimized geometries to be minima (no imaginary frequency) or transition states (TSs, having unique one imaginary frequency) were verified by harmonic frequency analysis. The ECD calculations for the stable conformers were carried out at the B3LYP/6-31G(d) level in the gas phase using Gaussian 09 software [27]. Boltzmann statistics were used to combine ECD spectra using SpecDis 1.64 software [28].

### 3.7. Biological Assay Protocols

#### 3.7.1. Anti-Inflammation Activity Assay

The murine RAW 264.7 macrophage cells (Shanghai Cell Bank of the Chinese Academy of Sciences, Shanghai, China) were cultured in Dulbecco’s modified Eagle’s medium (DMEM) supplemented with 10% (*v*/*v*) heatinactivated fetal bovine serum (FBS) and 1% penicillin–streptomycin (100 U/mL, Gibco) at 37 °C and maintained in 5% CO_2_ humidified air. RAW 264.7 cells (1 × 106 cells/well) were cultured in six-well plates with serum-free DMEM for 12 h and then pretreated with compound **1** (25, 50, and 60 μM) for 2 h prior to stimulation with LPS (1 μg/mL) for 18 h. The supernatant was harvested, and the concentrations of TNF-*α*, IL-1*β*, and IL-6 in the culture medium were determined using commercial ELISA kits according to the instructions.

#### 3.7.2. Antioxidant Activity Assay

The obtained compounds were evaluated for their antioxidant activities against DPPH and ABTS^+^ [24,29]. The effect of the compounds on DPPH radicals were estimated as previously reported. In summary, a methanolic DPPH solution was supplemented with compounds to achieve final concentrations of 6.25–100 µg/mL. The mixture was shaken and allowed to stand for 20 min at room temperature in the dark, after which the OD_517_ values were measured using a microplate reader (SYNERGY H1 BioTek). The scavenging rate (K%) was calculated as follows: K = (A _blank group_ − A)/A _blank group_ × 100%. Vitamin C served as the positive control. The free radical scavenging rate (K%) was calculated from the OD_517_ values, and EC_50_ was determined using IBM SPSS Statistics 25.

The stored solution of ABTS was diluted with 95% ethanol to ABST^+^ solution with an absorbance of 0.70 ± 0.02 at 734 nm. Then, the mixture containing 10 μL of sample solution and 190 μL of ABTS^+^ solution was cultured for 6 min at 25 °C in the dark. The mixture was shaken and allowed to stand for 7 min at room temperature in the dark, after which the OD_734_ values were measured using a microplate reader (SYNERGY H1 BioTek). The scavenging rate (SR%) was calculated as follows: SR = (A _blank group_ − A)/A _blank group_ × 100%. Vitamin C served as the positive control. The scavenging rate (SR%) was calculated from the OD_734_ values, and EC_50_ was determined using IBM SPSS statistics 25.

#### 3.7.3. Antibacterial Activity Assay

The antibacterial activities against three bacteria *Staphylococcus aureus* (ATCC700699, ATCC43300, ATCC29213) were evaluated in 96-well plates with a twofold serial dilution method described previously [30]. The minimum inhibitory concentration (MIC) values are shown in Table 4. The incubation conditions were as follows: fungi PDB medium (potato extract 0.4% and glucose 2%), 26 °C, 48 h; bacteria LB medium (yeast extract 0.5%, tryptone 1%, and NaCl 0.5%), 26 °C, 48 h.

### 3.8. Molecular Docking Studies

Molecular docking simulations were performed as previously described [31].

### 3.9. Statistical Analysis

The data obtained are presented as the means ± SD of three independent experiments. A one-way analysis of variance (ANOVA) test was used for statistical analysis. GraphPad Prism 5.02 (GraphPad Software Inc., San Diego, CA, USA) was used to perform the analyses.

## 4. Conclusions

In conclusion, five new metabolites, including three new naphthalene–chroman dimer derivatives (**1**–**3**) and two new chroman derivatives (**5** and **6**), together with five known ones (**4**, **7**–**10**), were isolated from the mangrove-sediment-derived fungus *Daldinia eschscholzii* HJX1P2. Their structures were determined by extensive spectroscopic analyses and ECD calculations. Compound **1** exhibited significant inhibitory effects on NO secretion in LPS-activated RAW 264.7 macrophage cells, with an IC_50_ value of 62.9 μM, and effectively suppressed the expression of IL-6 in LPS-induced RAW 264.7 cells. Compounds **7** and **8** showed DPPH radical scavenging activity, with IC_50_ values of 117.4 and 46.2 µM, respectively. Compounds **4** and **10** showed ABTS^+^ radical scavenging activity, with IC_50_ values of 66.6 and 33.2 µM, respectively. In addition, compounds **1**–**3**, **7,** and **9** showed weak antibacterial activity against *S. aureus* and two MRSA strains, with MIC values ranging from 74.4 to 390.6 μM. These results indicated that these naphthalene–chroman dimer derivatives could serve as promising lead molecules for the development of anti-inflammatory and antibacterial drugs.

## Figures and Tables

**Table 1:** ^1^H NMR spectroscopic data of compounds **1**–**4** in DMSO-*d*_6_.

Position	1	2	3	4
	*δ*_H_ (*J* in Hz)	*δ*_H_ (*J* in Hz)	*δ*_H_ (*J* in Hz)	*δ*_H_ (*J* in Hz)
1	-	-	7.15, d (8.4)	-
2	6.60 d (8.0)	6.90, m	6.70, d (8.4)	6.58, d (8.0)
3	6.80 d (8.0)	6.62, d (7.8)	-	6.62, d (8.0)
4	-	-	-	-
5	-	-	-	-
6	6.84 d (7.8)	6.94, m	6.94, d (7.2)	7.03, d (8.0)
7	7.45 t (7.8)	7.37, s	7.30, t (7.2)	7.49, t (8.0)
8	7.70 d (7.8)	7.77, s	7.34, d (7.8)	7.84, d (8.8)
9	-	-	-	-
10	-	-	-	-
1′	4.80, d (5.6)	4.79, s	4.61, d (4.8)	4.81, d (5.2)
2′	*α*, 1.86, d (13.6), *β*, 1.95. ddd (13.6, 11.6, 5.6)	*α*, 1.67, m, *β*, 2.48, m	*α*, 1.81, dt (13.6, 5.8), *β*, 2.06, d (13.6)	*α*, 1.84, m *β*, 1.94, m
3′	3.87, m	4.10, m	3.87, ddq (1.6, 6.0, 12.4)	3.85, m
5′	6.35, d (8.4)	6.35, d (7.4)	6.34, d (7.8)	6.35, d (8.0)
6′	6.97, t (8.4)	6.93, m	6.97, d (8.4)	6.97, t (8.0)
7′	6.35, d (8.4)	6.25, d (7.4)	6.33, d (7.8)	6.35, d (8.0)
8′	-	-	-	-
9′	-	-	-	-
10′	-	-	-	-
3′-Me	1.18, d (6.2)	1.24, d (6.2)	1.21, d (6.6)	1.17, d (6.0)
8′-OH	9.62, s	8.90, s	9.18, s	9.16, s
4/5-OH	9.36, s	9.34, s	9.82, s	9.41, s
4/5-OMe	3.96, s	4.02, s	4.06, s	4.04, s

**Table 2:** ^13^C NMR spectroscopic data of compounds **1**–**4** in DMSO-*d*_6_.

Position	1	2	3	4
	*δ*_C_, Type	*δ*_C_, Type	*δ*_C_, Type	*δ*_C_, Type
1	134.3, C	127.1, C	117.7, CH	131.7, C
2	125.5, CH	127.1, CH (overlapped)	127.6, CH	127.0, CH
3	103.5, CH	110.1, CH	126.4, C	109.0, CH
4	154.4, C	152.0, C	149.9, C	152.5, C
5	155.0, C	156.6, C (overlapped)	155.7, C	156.7, C
6	110.0, CH	104.1, CH	104.4, CH	104.6, CH
7	127.6, CH	125.7, CH	125.5, CH	126.4, CH
8	114.2, CH	117.0, CH	120.9, CH	116.7, CH
9	133.3, C	133.7, C	134.7, C	133.1, C
10	114.8, C	114.7, C	114.3, C	115.0, C
1′	31.3, CH	35.3, CH	29.5, CH	31.2, CH
2′	35.0, CH_2_	41.7, CH_2_	34.8, CH_2_	35.4, CH_2_
3′	66.8, CH	71.8, CH	67.3, CH	66.8, CH
5′	106.8, CH	107.6, CH	106.9, CH	106.9, CH
6′	127.7, CH	127.1, CH (overlapped)	127.8, CH	127.6, CH
7′	106.5, CH	107.5, CH	106.4, CH	106.5, CH
8′	155.7, C	156.6, C	155.7, C	155.6, C
9′	156.4, C	158.0, C	156.5, C	156.6, C
10′	110.6, C	113.6, C	110.5, C	110.7, C
4/5-OMe	56.1, OMe	56.3, OMe	56.3, OMe	56.4, OMe
3′-Me	21.0, CH_3_	21.1, CH_3_	21.3, CH_3_	21.1, CH_3_

**Table 3:** ^1^H and ^13^C NMR spectroscopic data of compounds **5** and **6** in CDCl_3_.

Position	5		6	
	*δ*_H_ (*J* in Hz)	*δ*_C_	*δ*_H_ (*J* in Hz)	*δ*_C_
1	-	162.2, C	-	-
2	6.47, d (8.4)	108.9, CH	4.06, dq (9.6, 6.0)	71.1, CH
3	7.34, t (8.4)	138.2, CH	3.61, m	71.2, CH
4	6.36, d (8.4)	107.7, CH	4.97, d (4.2)	61.5, CH
4a	-	158.9, C	-	112.2, C
5	*α*, 2.41, m; *β*, 1.39, m	46.1, CH_2_	-	158.9, C
6	4.13, m	66.0, CH	6.47, d (8.4)	102.4, CH
7	*α*, 2.06, d (12.0) *β*, 1.09, m	42.6, CH_2_	7.17, dd (8.4, 8.4)	130.3, CH
8	1.86, m	30.6, CH	6.51, d (8.4)	109.9, CH
8a	1.85, br s	58.5, CH	-	155.2, C
2-Me	-	-	1.49, d (6.0)	17.6, CH_3_
5-OMe	-	-	3.88, s	55.8, CH_3_
9	-	201.3, C	-	-
9a	-	106.3, C	-	-
10	-	81.3, C	-	-
11	1.35, s	24.4, CH_3_	-	-
12	0.98, d (6.2)	19.5, CH_3_	-	-
1-OH	11.59, s	-	-	-
6-OH	3.49, s	-	-	-

**Table 4:** MIC values (µM) of antibacterial activity of compounds **1**–**3**, **7,** and **9**.

Compounds	*S. aureus* (ATCC 29213)	MRSA (ATCC 700699)	MRSA (ATCC 43300)
**1**	74.4	-	-
**2**	74.4	-	-
**3**	148.8	-	-
**7**	-	130.2	390.6
**9**	-	131.6	263.2
Vancomycin	1.0	1.0	1.0

“-” signifies MIC > 500 μM, positive control: vancomycin.

## Data Availability

The data for the research results can be obtained from supporting information.

## Supplementary Materials

The following supporting information can be downloaded at: https://www.mdpi.com/article/10.3390/md23060238/s1, Figure S1. The ^1^H NMR spectrum of **1** in DMSO-*d*_6_; Figure S2. The ^13^C NMR spectrum of **1** in DMSO-*d*_6_; Figure S3. The DEPT-135 spectrum of **1** in DMSO-*d*_6_; Figure S4. The HSQC spectrum of **1** in DMSO-*d*_6_; Figure S5. The ^1^H-^1^H COSY spectrum of **1** in DMSO-*d*_6_; Figure S6. The HMBC spectrum of 1 in DMSO-*d*_6_; Figure S7. The NOESY spectrum of **1** in DMSO-*d*_6_; Figure S8. The HR-ESI-MS spectrum of **1**; Figure S9. The ^1^H NMR spectrum of **2** in DMSO-*d*_6_; Figure S10. The ^13^C NMR spectrum of **2** in DMSO-*d*_6_; Figure S11. The DEPT-135 spectrum of **2** in DMSO-*d*_6_; Figure S12. The HSQC spectrum of **2** in DMSO-*d*_6_; Figure S13. The ^1^H-^1^H COSY spectrum of **2** in DMSO-*d*_6_; Figure S14. The HMBC spectrum of **2** in DMSO-*d*_6_; Figure S15. The NOESY spectrum of **2** in DMSO-*d*_6_; Figure S16. The HR-ESI-MS spectrum of **2**; Figure S17. The ^1^H NMR spectrum of **3** in DMSO-*d*_6_; Figure S18. The ^13^C NMR spectrum of **3** in DMSO-*d*_6_; Figure S19. The DEPT-135 spectrum of **3** in DMSO-*d*_6_; Figure S20. The HSQC spectrum of **3** in DMSO-*d*_6_; Figure S21. The ^1^H-^1^H COSY spectrum of **3** in DMSO-*d*_6_; Figure S22. The HMBC spectrum of **3** in DMSO-*d*_6_; Figure S23. The NOESY spectrum of **3** in DMSO-*d*_6_; Figure S24. The HR-ESI-MS spectrum of **3**; Figure S25. The ^1^H NMR spectrum of **5** in CDCl_3_; Figure S26. The ^13^C NMR spectrum of **5** in CDCl_3_; Figure S27. The DEPT-135 spectrum of **5** in CDCl_3_; Figure S28. The HSQC spectrum of **5** in CDCl_3_; Figure S29. The ^1^H-^1^H COSY spectrum of **5** in CDCl_3_; Figure S30. The HMBC spectrum of **5** in CDCl_3_; Figure S31. The NOESY spectrum of **5** in CDCl_3_; Figure S32. The HR-ESI-MS spectrum of **5**; Figure S33. The ^1^H NMR spectrum of **6** in CDCl_3_; Figure S34. The ^13^C NMR spectrum of **6** in CDCl_3_; Figure S35. The DEPT-135 spectrum of **6** in CDCl_3_; Figure S36. The HSQC spectrum of **6** in CDCl_3_; Figure S37. The ^1^H-^1^H COSY spectrum of **6** in CDCl_3_; Figure S38. The HMBC spectrum of **6** in CDCl_3_; Figure S39. The NOESY spectrum of **6** in CDCl_3_; Figure S40. The HR-ESI-MS spectrum of **6**; Figure S41. The ^1^H NMR spectrum of **2** in CD_3_OD-*d*_4_; Figure S42. The ^13^C NMR spectrum of **2** in CD_3_OD-*d*_4_; Figure S43. The DEPT-135 spectrum of **2** in CD_3_OD-*d*_4_; Figure S44. The HSQC spectrum of **2** in CD_3_OD-*d*_4_; Figure S45. The UV spectrum of **1**; Figure S46. The UV spectrum of **2**; Figure S47. The UV spectrum of **3**; Figure S48. The UV spectrum of **5**; Figure S49. The UV spectrum of **6**; Figure S50. The IR spectrum of **1**; Figure S51. The IR spectrum of **2**; Figure S52. The IR spectrum of **3**; Figure S53. The IR spectrum of **5**; Figure S54. The IR spectrum of **6**; Figure S55. The ^1^H NMR spectrum of **4** in DMSO-*d*_6_; Figure S56. The ^13^C NMR spectrum of **4** in DMSO-*d*_6_; Figure S57. The ^1^H NMR spectrum of **7** in DMSO-*d*_6_; Figure S58. The ^13^C NMR spectrum of **7** in DMSO-*d*_6_; Figure S59. The ^1^H NMR spectrum of **8** in CDCl_3_; Figure S60. The ^13^C NMR spectrum of **8** in CDCl_3_; Figure S61. The ^1^H NMR spectrum of **9** in CD_3_OD; Figure S62. The ^13^C NMR spectrum of **9** in CD_3_OD; Figure S63. The ^1^H NMR spectrum of **10** in CDCl_3_; Figure S64. The ^13^C NMR spectrum of **10** in CDCl_3_.
