# Supplementary material for: New Bioactive Polyketides from the Mangrove-Derived Fungus Daldinia eschscholzii HJX1P2
Source: Mar Drugs. 2025 May 30;23(6):238. doi: 10.3390/md23060238 (PMC12193800; doi:10.3390/md23060238)
Supplement: Supplementary file 1 [file marinedrugs-23-00238-s001.zip › marinedrugs-3652912-supplementary.pdf]

New Bioactive Polyketides from the Mangrove-Derived Fungus *Daldinia eschscholzii* HJX1P2

Miao Yu [1,2#], Yikang Qiu [1,2#], Shiji Chen [1,2], Jueying Shi [1,2], Xiu Gong [1,2], Jiayi Feng [1,2], Fangru Lin [1,2], Weinv Zeng[3], Wenyuan Kang [1,2], Caijuan Zheng [1,2,]* and Guolei Huang [1,2,]*

1 Key Laboratory of Tropical Medicinal Resource Chemistry of Ministry of Education, College of Chemistry and Chemical Engineering, Hainan Normal University, Haikou 571158, China; yumiaonpc@126.com (M.Y.); qyk7747226@sina.com (Y.Q.); chenshijinpc@126.com (S.C.); jueying202406@163.com (J.S.); 15228548005@163.com (X.G.); 19308057394@163.com (J.F.); fl326688@163.com (F.L.); hy0322027@muhn.edu.cn (W.Z.); kangsgo@hainnu.edu.cn (W.K.)

2 Key Laboratory of Tropical Medicinal Plant Chemistry of Hainan Province, Haikou 571158, China

3 International Center for Aging and Cancer, Hainan Medical University

* Correspondence: huangguolei1982@163.com (G.H.); caijuan2002@163.com (C.Z.)

New Bioactive Polyketides from the Mangrove-Derived Fungus *Daldinia eschscholzii* HJX1P2

Content

Figure S1. The ^{1}H NMR spectrum of **1** in DMSO-d_6

Figure S2. The ^{13}C NMR spectrum of **1** in DMSO-d_6

Figure S3. The DEPT-135 spectrum of **1** in DMSO-d_6

Figure S4. The HSQC spectrum of **1** in DMSO-d_6

Figure S5. The ^{1}H-^{1}H COSY spectrum of **1** in DMSO-d_6

Figure S6. The HMBC spectrum of **1** in DMSO-d_6

Figure S7. The NOESY spectrum of **1** in DMSO-d_6

Figure S8. The HR-ESI-MS spectrum of **1**

Figure S9. The ^{1}H NMR spectrum of **2** in DMSO-d_6

Figure S10. The ^{13}C NMR spectrum of **2** in DMSO-d_6

Figure S11. The DEPT-135 spectrum of **2** in DMSO-d_6

Figure S12. The HSQC spectrum of **2** in DMSO-d_6

Figure S13. The ^{1}H-^{1}H COSY spectrum of **2** in DMSO-d_6

Figure S14. The HMBC spectrum of **2** in DMSO-d_6

Figure S15. The NOESY spectrum of **2** in DMSO-d_6

Figure S16. The HR-ESI-MS spectrum of **2**

Figure S17. The ^{1}H NMR spectrum of **3** in DMSO-d_6

Figure S18. The ^{13}C NMR spectrum of **3** in DMSO-d_6

Figure S19. The DEPT-135 spectrum of **3** in DMSO-d_6

Figure S20. The HSQC spectrum of **3** in DMSO-d_6

Figure S21. The ^{1}H-^{1}H COSY spectrum of **3** in DMSO-d_6

Figure S22. The HMBC spectrum of **3** in DMSO-d_6

Figure S23. The NOESY spectrum of **3** in DMSO-d_6

Figure S24. The HR-ESI-MS spectrum of **3**

Figure S25. The ^{1}H NMR spectrum of **5** in $CDCl_3$

Figure S26. The ^{13}C NMR spectrum of **5** in $CDCl_3$

Figure S27. The DEPT-135 spectrum of **5** in $CDCl_3$

Figure S28. The HSQC spectrum of **5** in $CDCl_3$

Figure S29. The ^{1}H-^{1}H COSY spectrum of **5** in $CDCl_3$

Figure S30. The HMBC spectrum of **5** in $CDCl_3$

Figure S31. The NOESY spectrum of **5** in $CDCl_3$

Figure S32. The HR-ESI-MS spectrum of **5**

Figure S33. The ^{1}H NMR spectrum of **6** in $CDCl_3$

Figure S34. The ^{13}C NMR spectrum of **6** in $CDCl_3$

Figure S35. The DEPT-135 spectrum of **6** in $CDCl_3$

Figure S36. The HSQC spectrum of **6** in $CDCl_3$

Figure S37. The ^{1}H-^{1}H COSY spectrum of **6** in $CDCl_3$

Figure S38. The HMBC spectrum of **6** in $CDCl_3$

Figure S39. The NOESY spectrum of **6** in $CDCl_3$

Figure S40. The HR-ESI-MS spectrum of **6**

Figure S41. The ^{1}H NMR spectrum of **2** in CD_3OD-d_4

Figure S42. The ^{13}C NMR spectrum of **2** in CD_3OD-d_4

Figure S43. The DEPT-135 spectrum of **2** in CD_3OD-d_4

Figure S44. The HSQC spectrum of **2** in CD_3OD-d_4

Figure S45. The UV spectrum of **1**

Figure S46. The UV spectrum of **2**

Figure S47. The UV spectrum of **3**

Figure S48. The UV spectrum of **5**

Figure S49. The UV spectrum of **6**

Figure S50. The IR spectrum of **1**

Figure S51. The IR spectrum of **2**

Figure S52. The IR spectrum of **3**

Figure S53. The IR spectrum of **5**

Figure S54. The IR spectrum of **6**

Figure S55. The ^{1}H NMR spectrum of **4** in DMSO-d_6

Figure S56. The ^{13}C NMR spectrum of **4** in DMSO-d_6

Figure S57. The ^{1}H NMR spectrum of **7** in DMSO-d_6

Figure S58. The ^{13}C NMR spectrum of **7** in DMSO-d_6

Figure S59. The ^{1}H NMR spectrum of **8** in $CDCl_3$

Figure S60. The ^{13}C NMR spectrum of **8** in $CDCl_3$

Figure S61. The ^{1}H NMR spectrum of **9** in CD_3OD

Figure S62. The ^{13}C NMR spectrum of **9** in CD_3OD

Figure S63. The ^{1}H NMR spectrum of **10** in $CDCl_3$

Figure S64. The ^{13}C NMR spectrum of **10** in $CDCl_3$

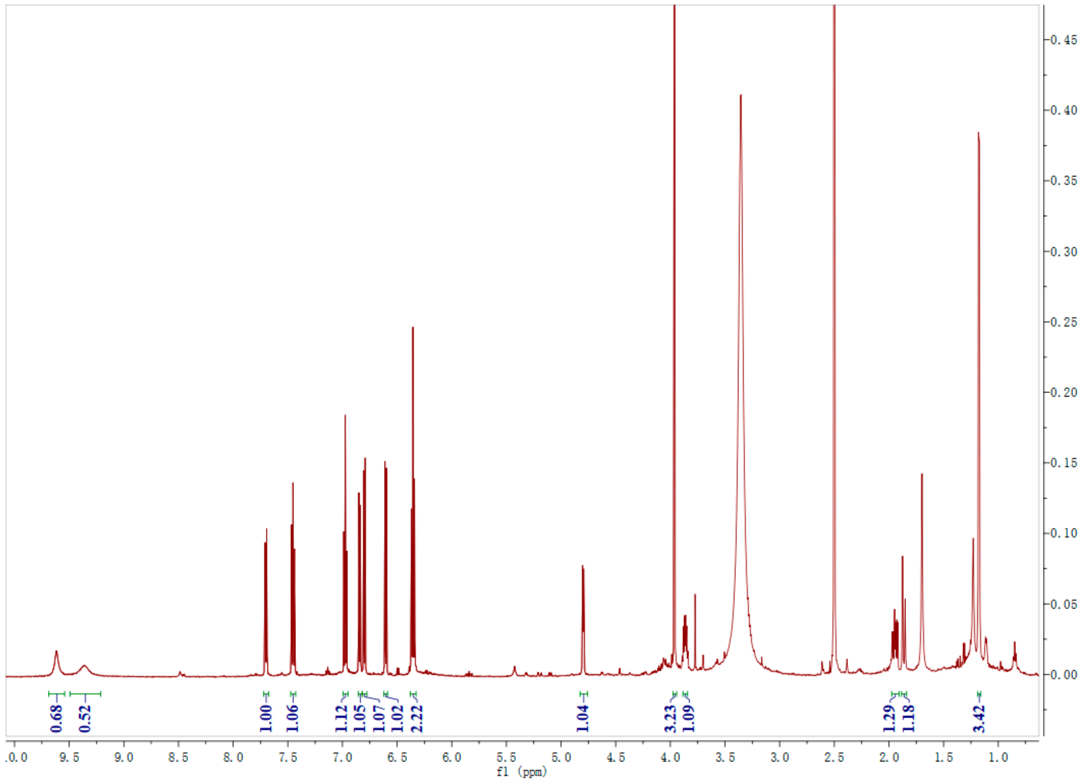

Figure S1. The ^{1}H NMR spectrum of **1** in DMSO-d_6

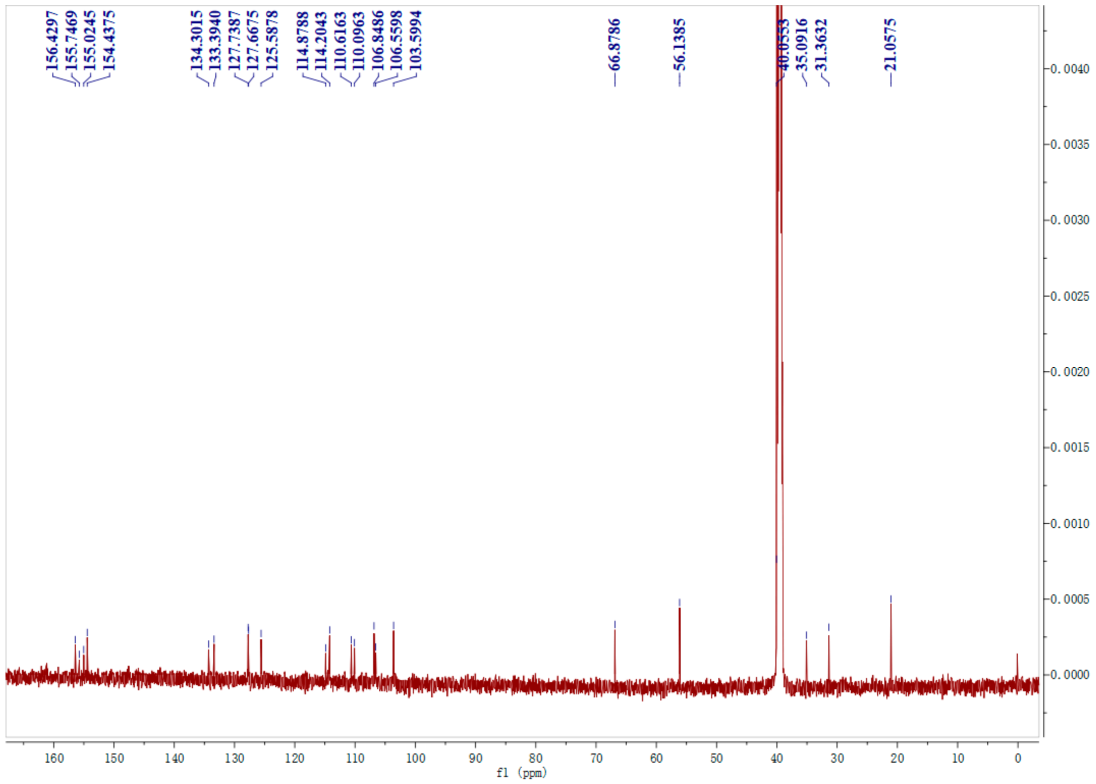

Figure S2. The ^{13}C NMR spectrum of **1** in DMSO-d_6

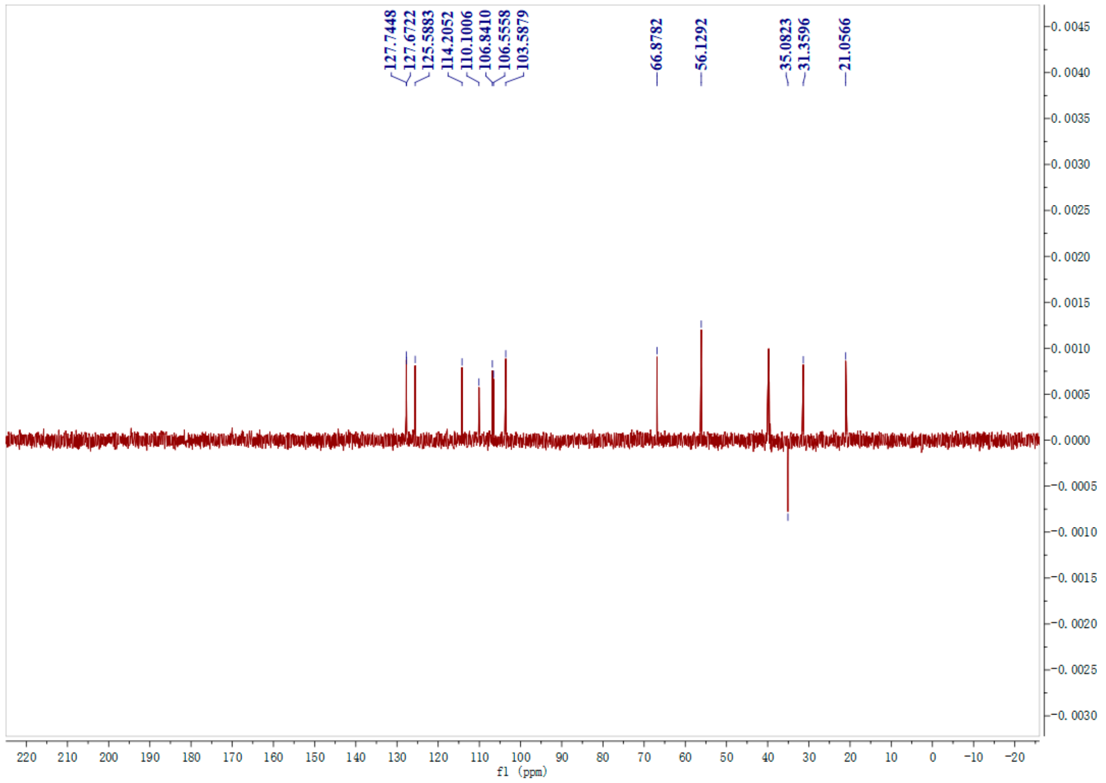

Figure S3. The DEPT-135 spectrum of **1** in DMSO-d_6

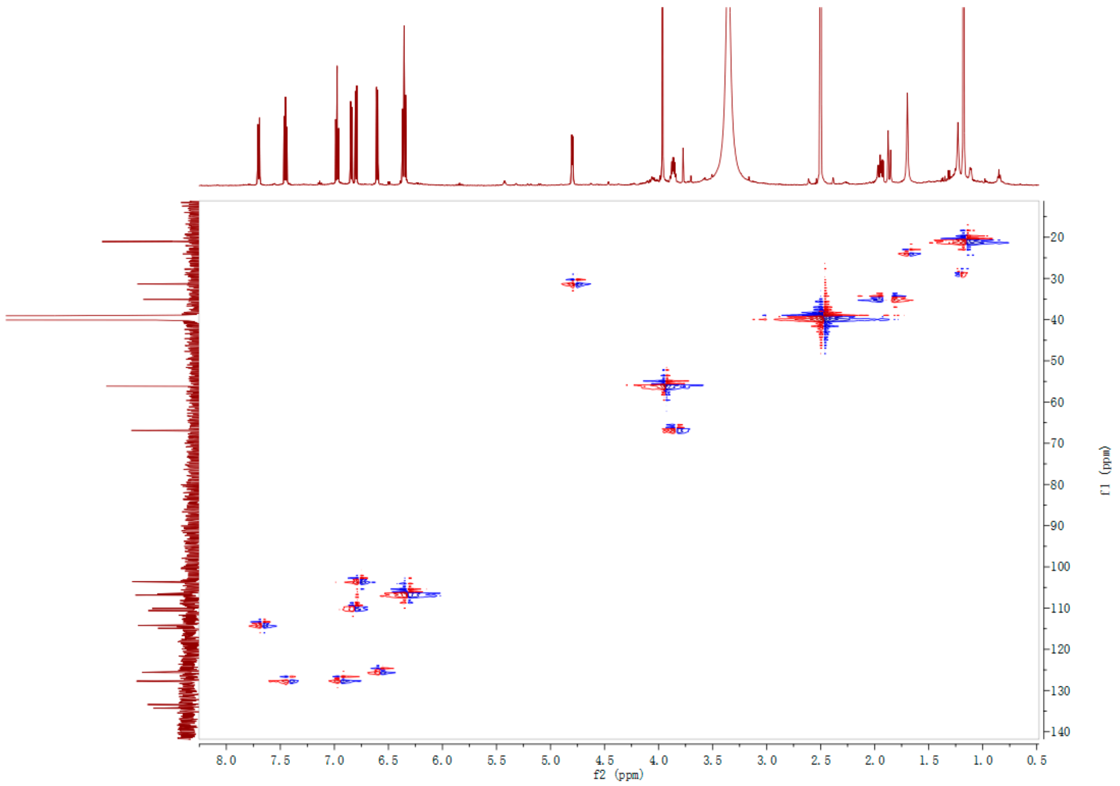

Figure S4. The HSQC spectrum of **1** in DMSO-d_6

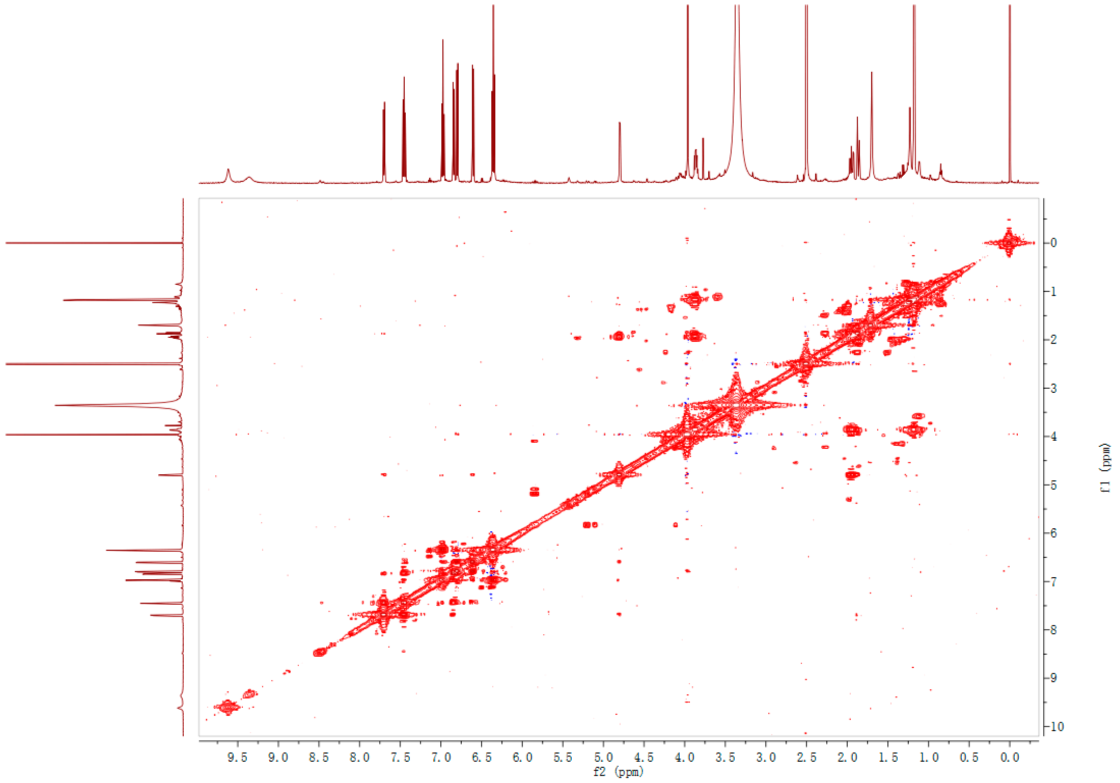

Figure S5. The COSY spectrum of **1** in DMSO-d_6

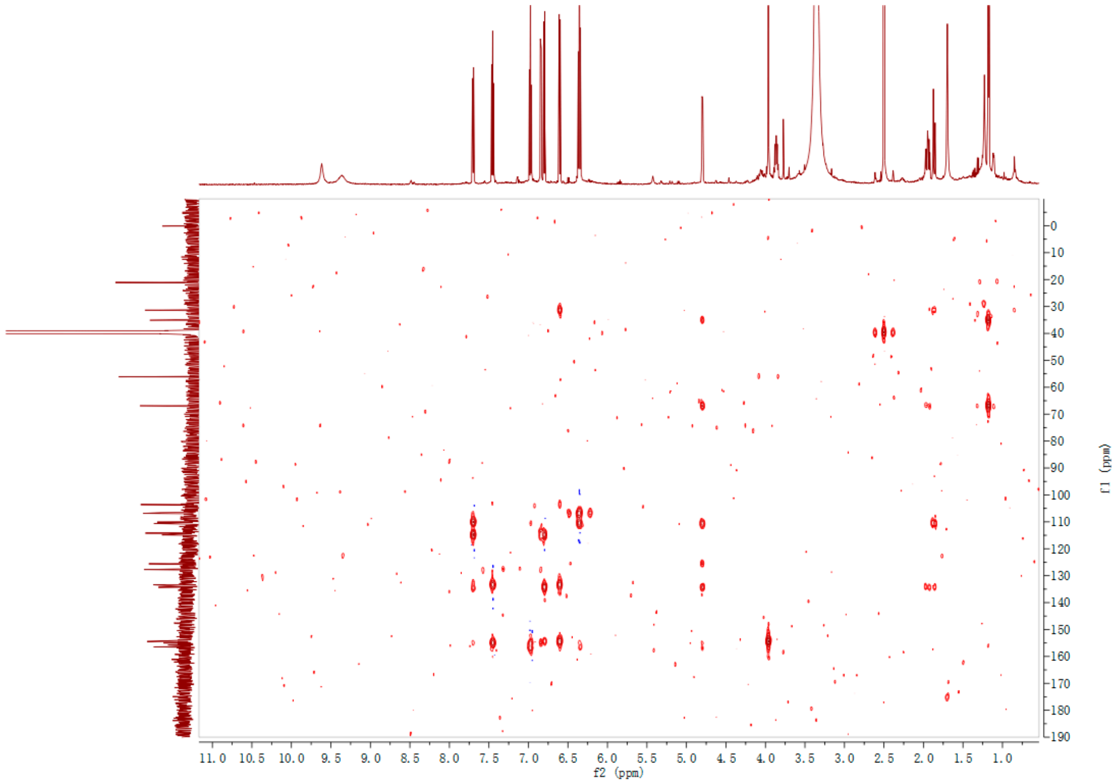

Figure S6. The HMBC spectrum of **1** in DMSO-d_6

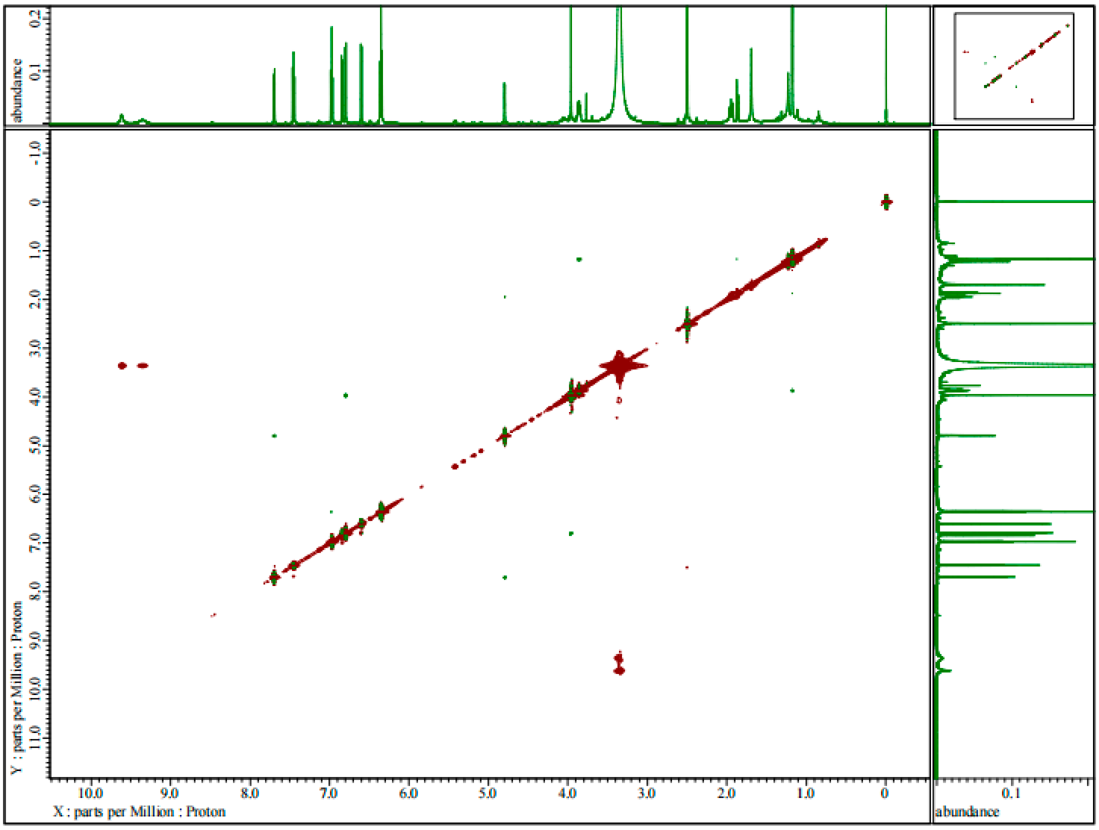

Figure S7. The NOESY spectrum of **1** in DMSO-d_6

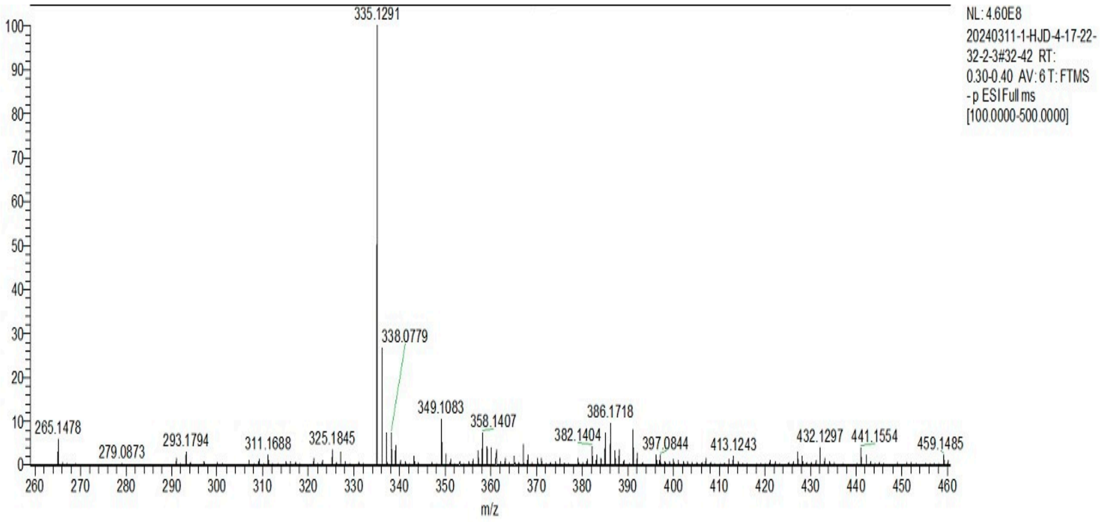

Figure S8. The HR-ESI-MS spectrum of **1**

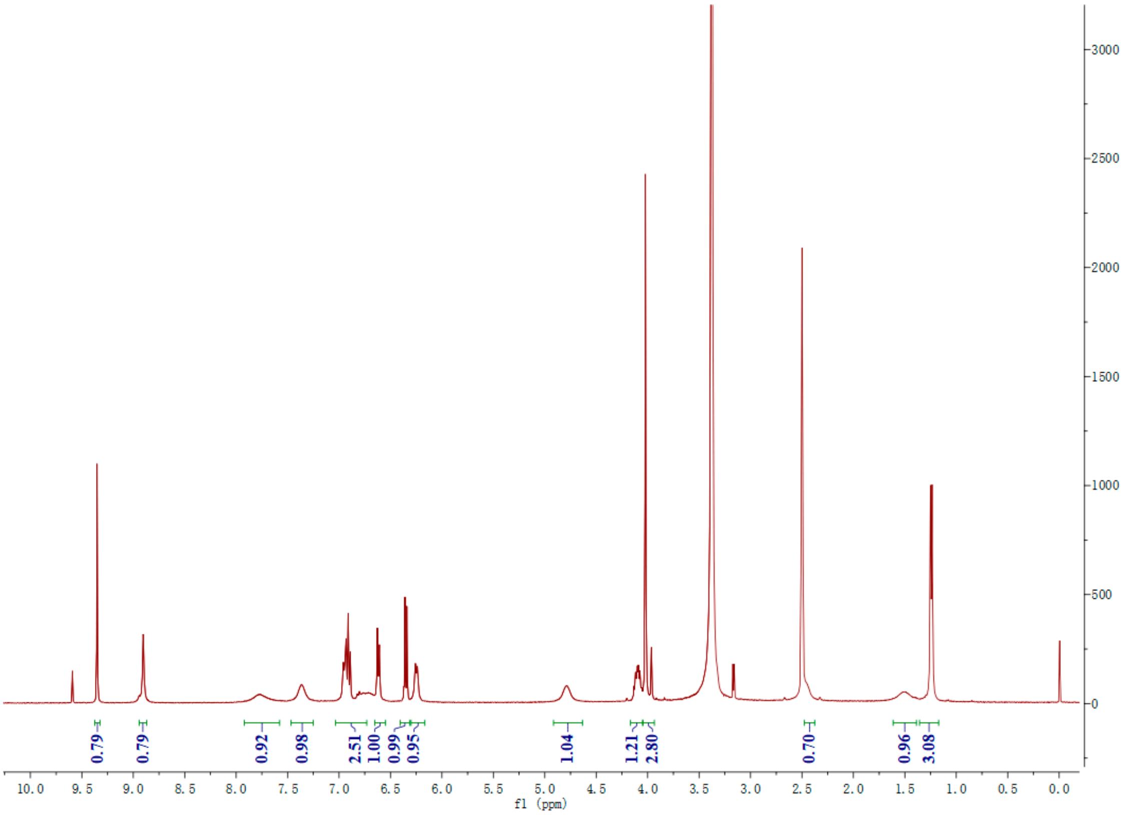

Figure S9. The ^{1}H NMR spectrum of **2** in DMSO-*d*6

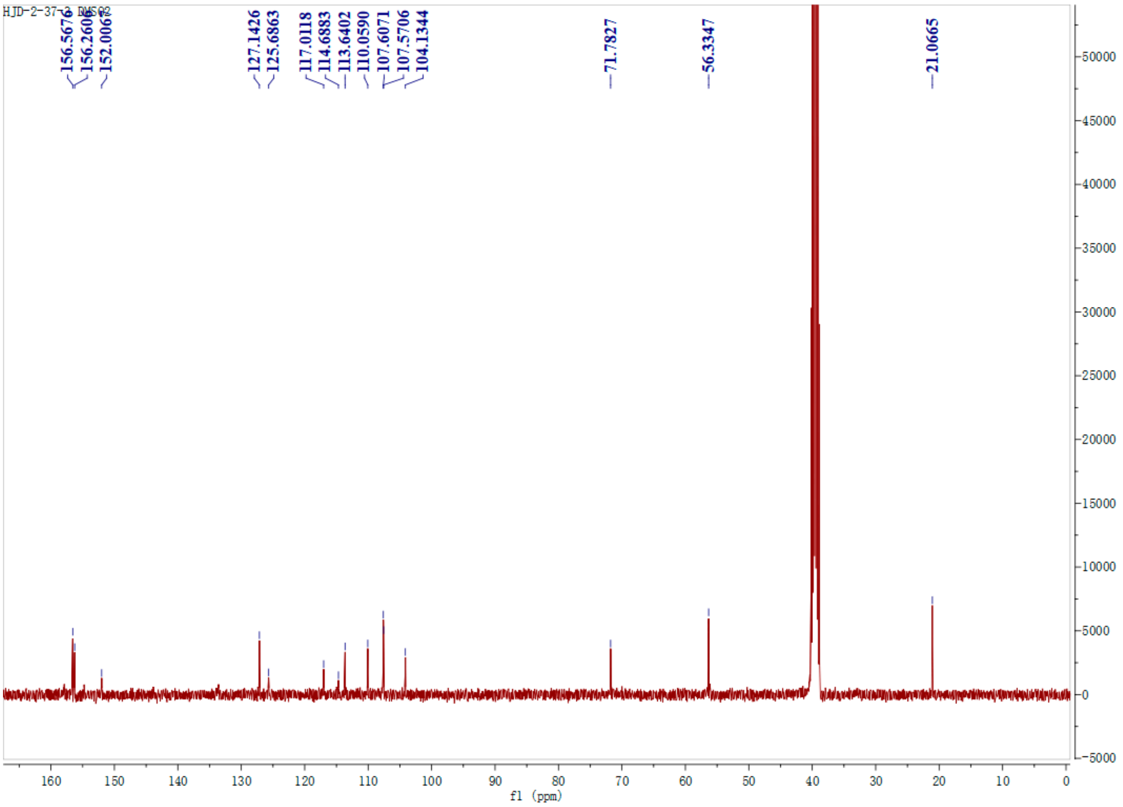

Figure S10. The ^{13}C NMR spectrum of **2** in DMSO-*d*6

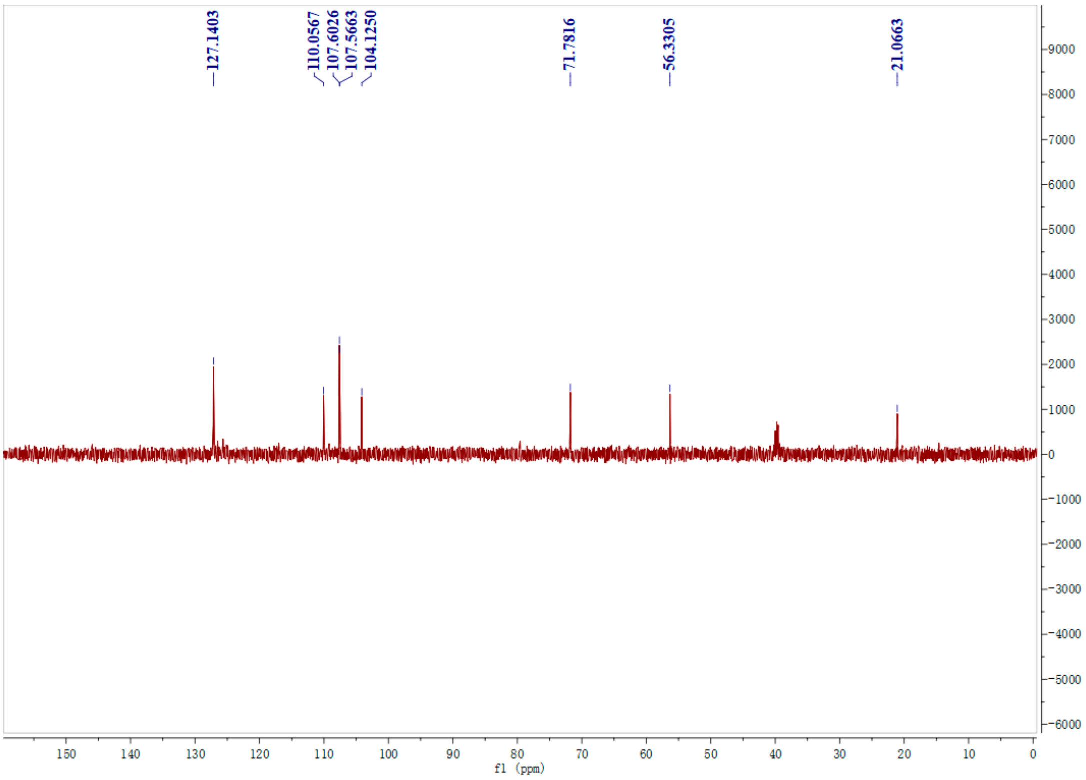

Figure S11. The DEPT-135 spectrum of **2** in DMSO-d_6

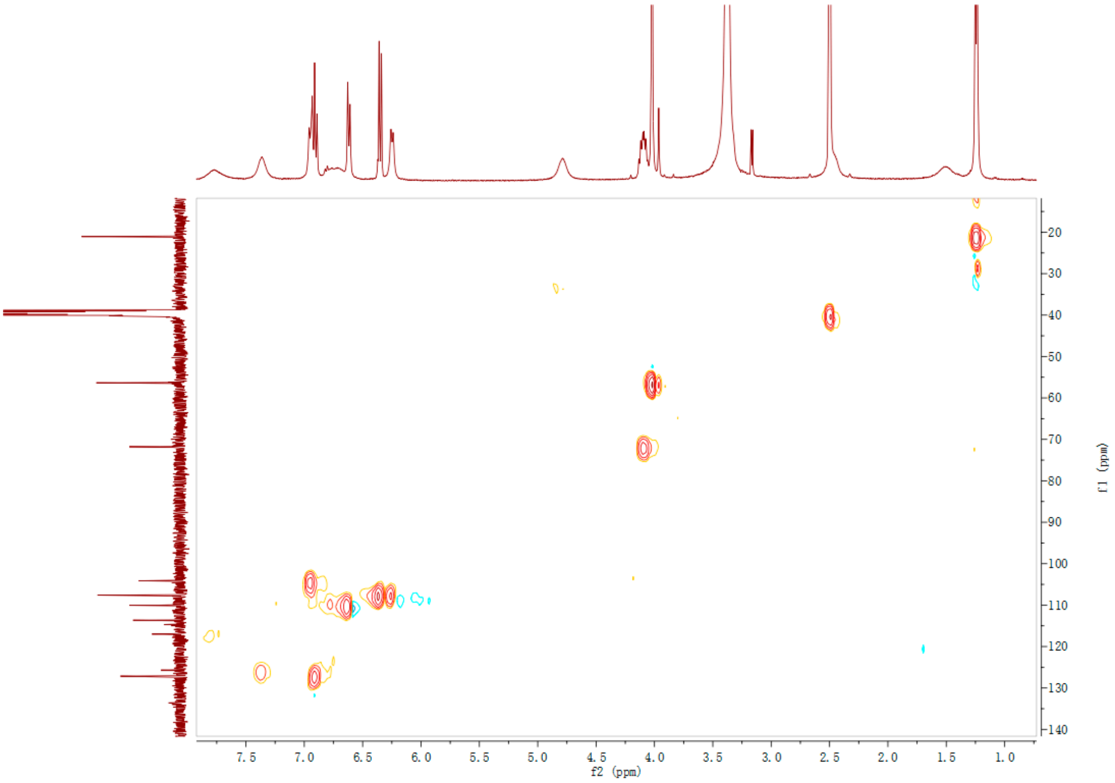

Figure S12. The HSQC spectrum of **2** in DMSO-d_6

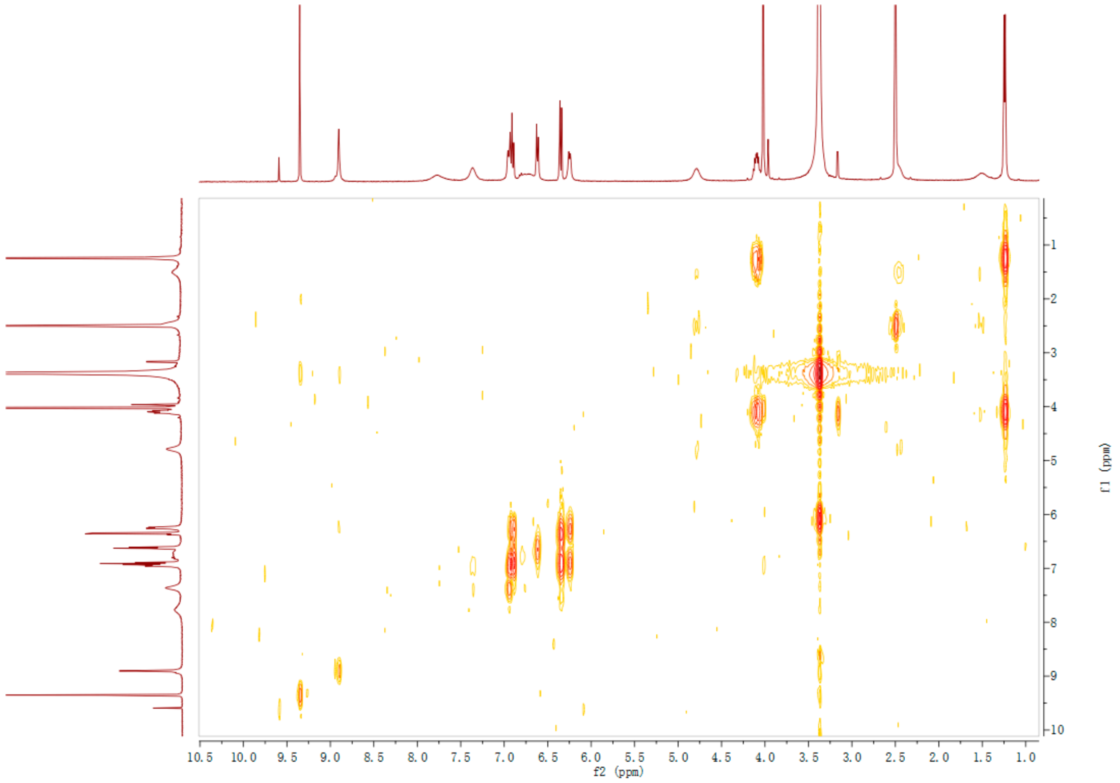

Figure S13. The ^{1}H-^{1}H COSY spectrum of **2** in DMSO-*d*6

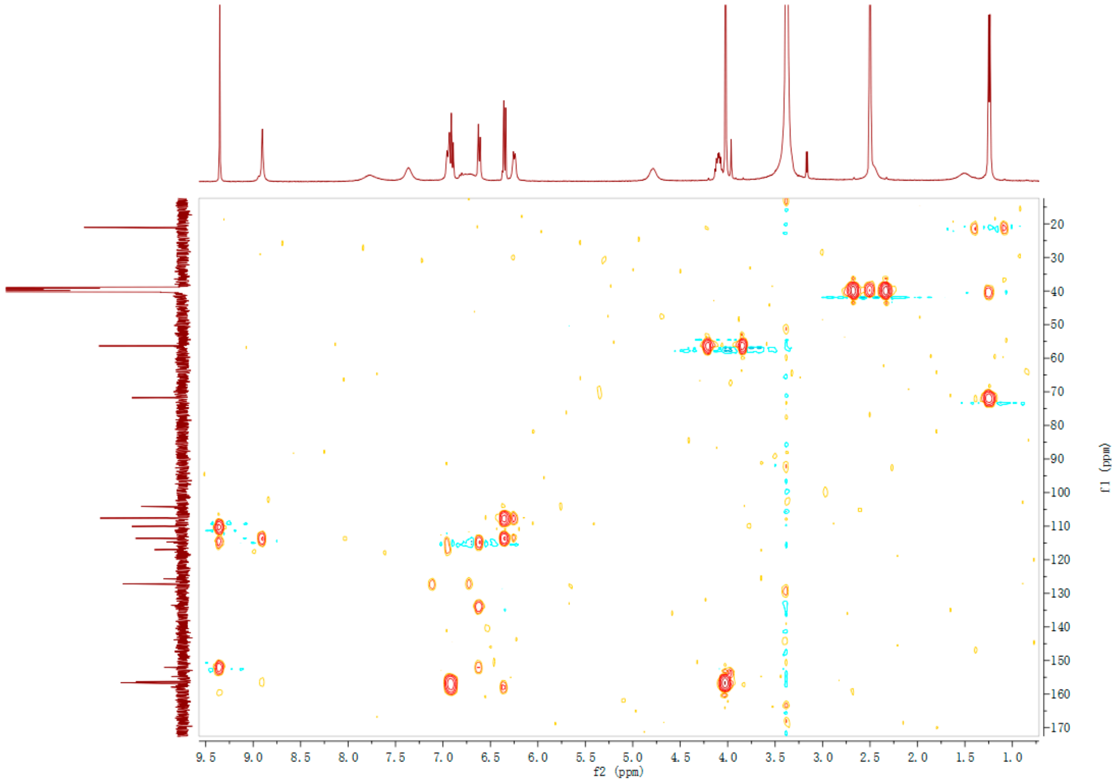

Figure S14. The HMBC spectrum of **2** in DMSO-*d*6

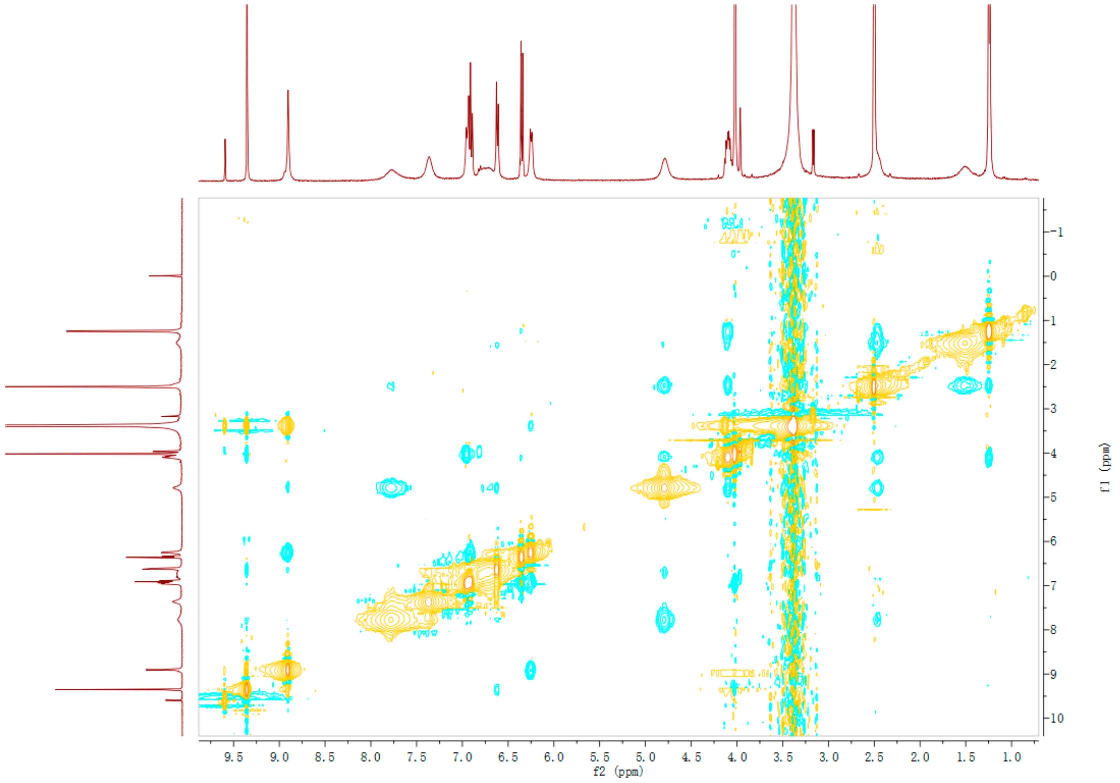

Figure S15. The ^{1}H-^{1}H NOESY spectrum of **2** in DMSO-d_6

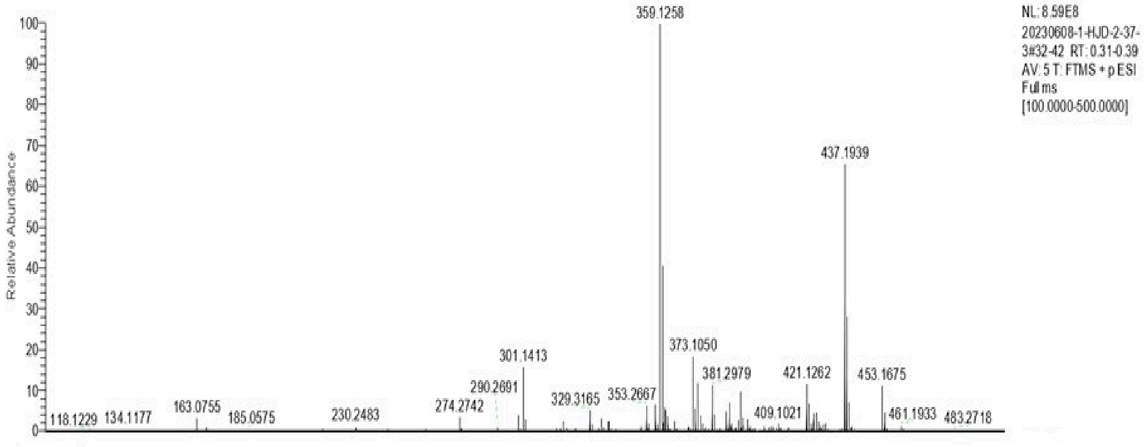

Figure S16. The HR-ESI-MS spectrum of **2** in CD_3OD

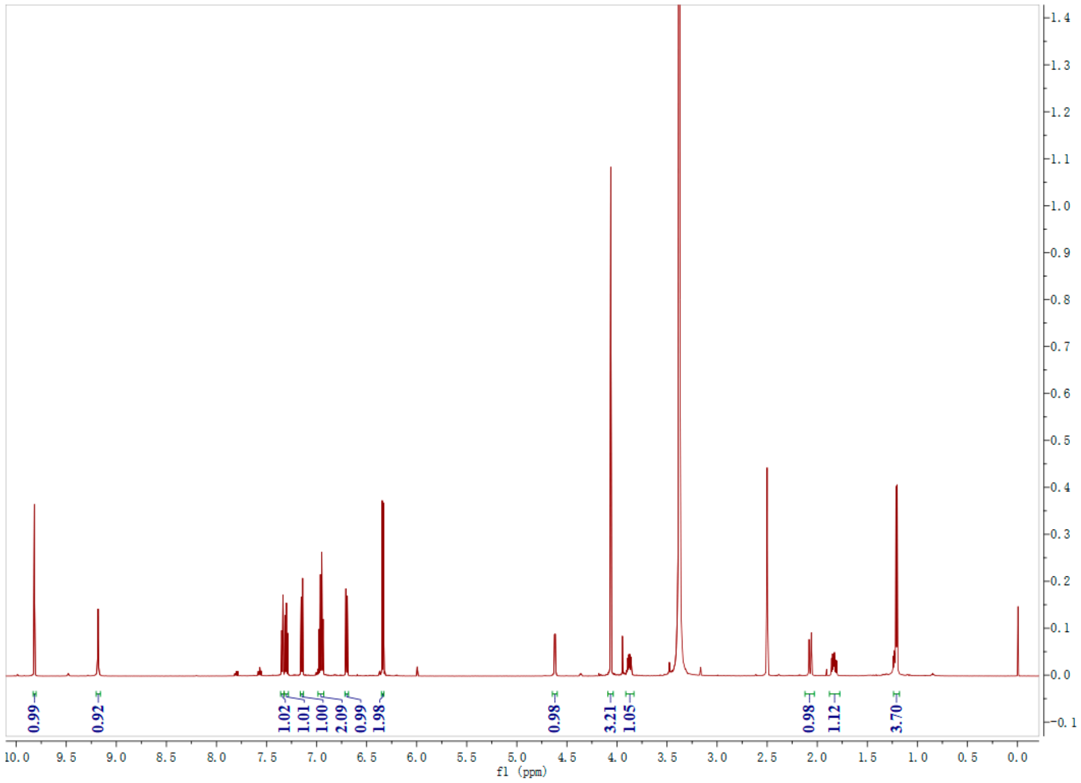

Figure S17. The ^{1}H NMR spectrum of **3** in DMSO-d_6

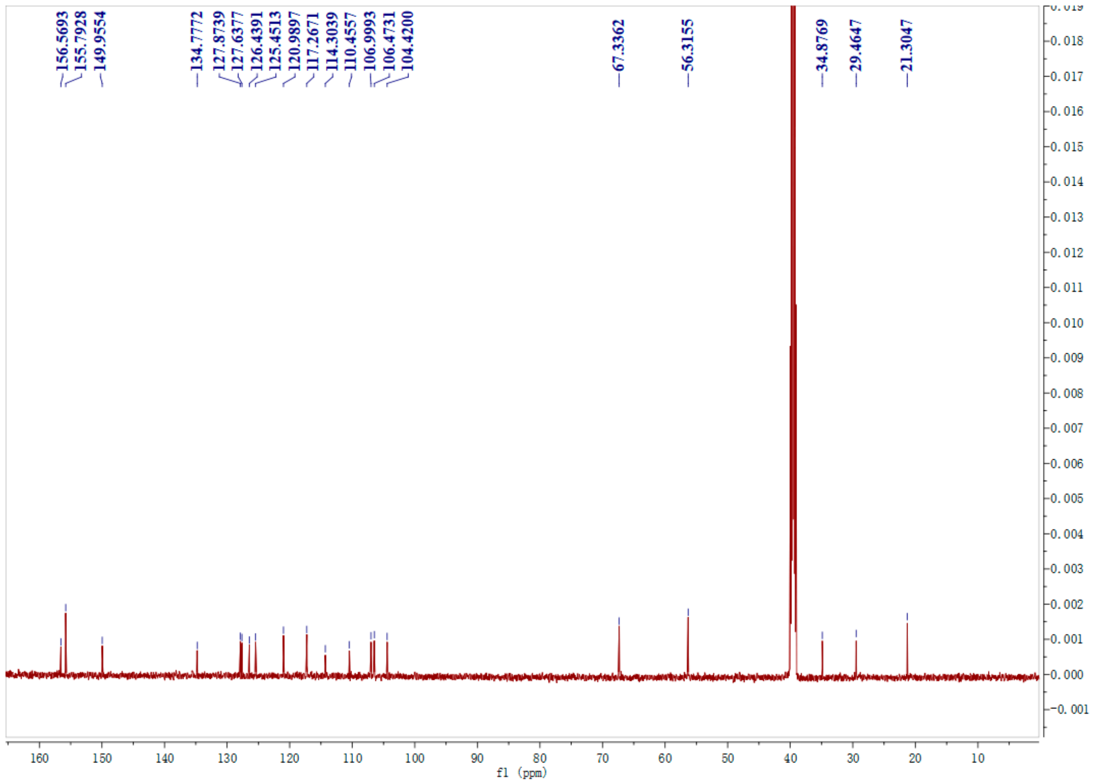

Figure S18. The ^{13}C NMR spectrum of **3** in DMSO-d_6

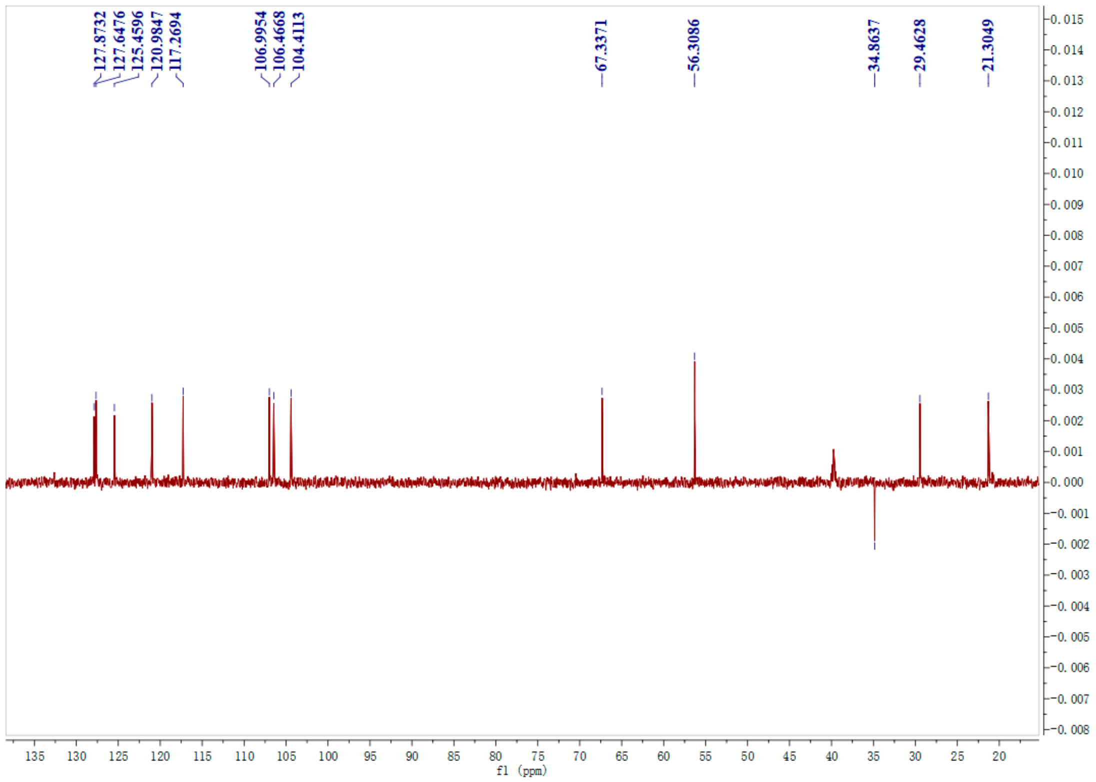

Figure S19. The DEPT-135 spectrum of **3** in DMSO-*d*6

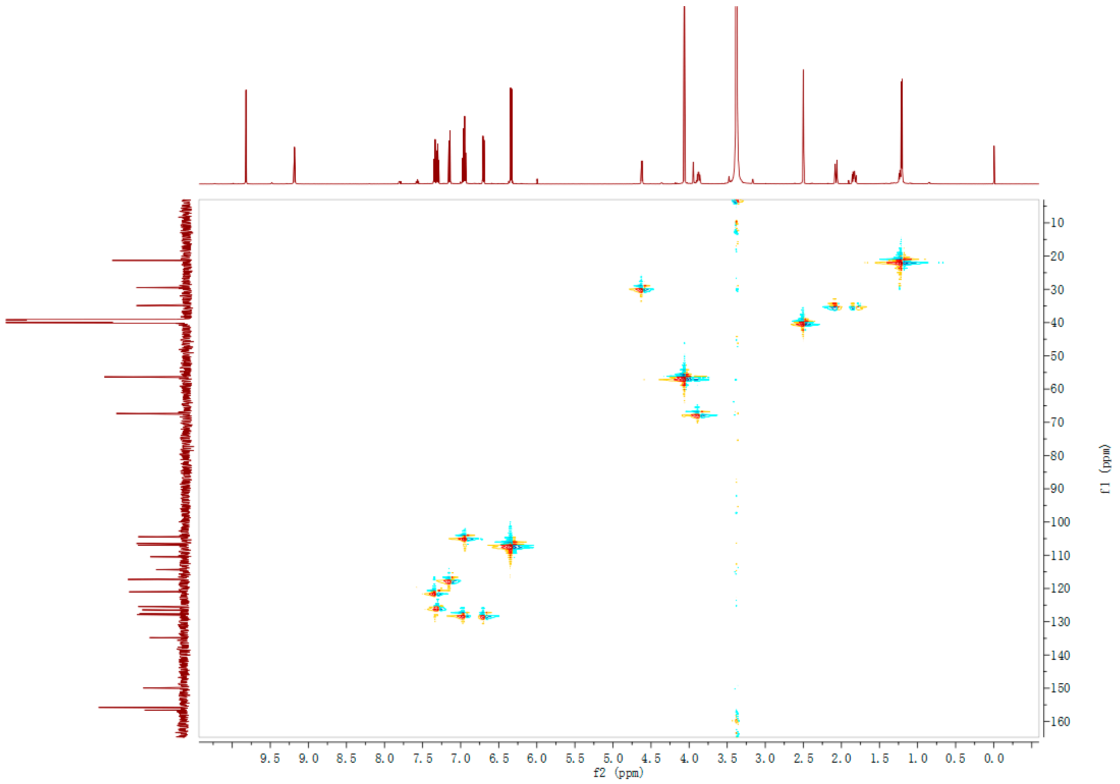

Figure S20. The HSQC spectrum of **3** in DMSO-*d*6

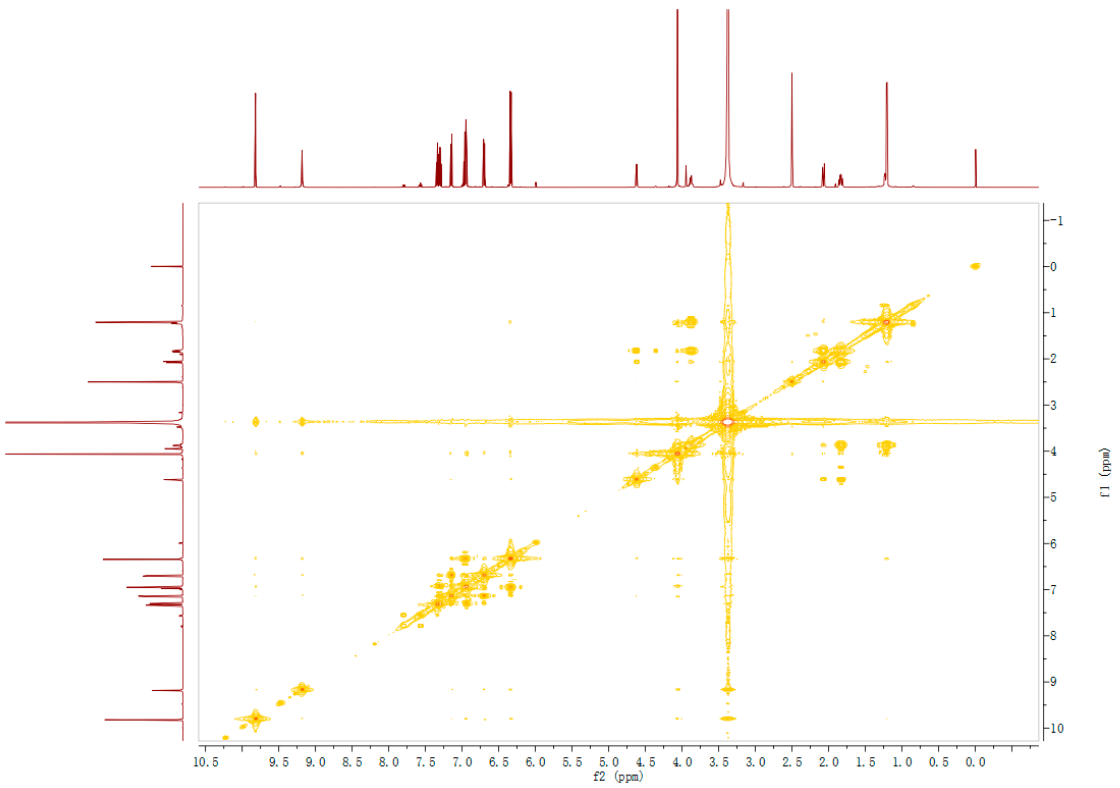

Figure S21. The ^{1}H-^{1}H COSY spectrum of **3** in DMSO-*d*6

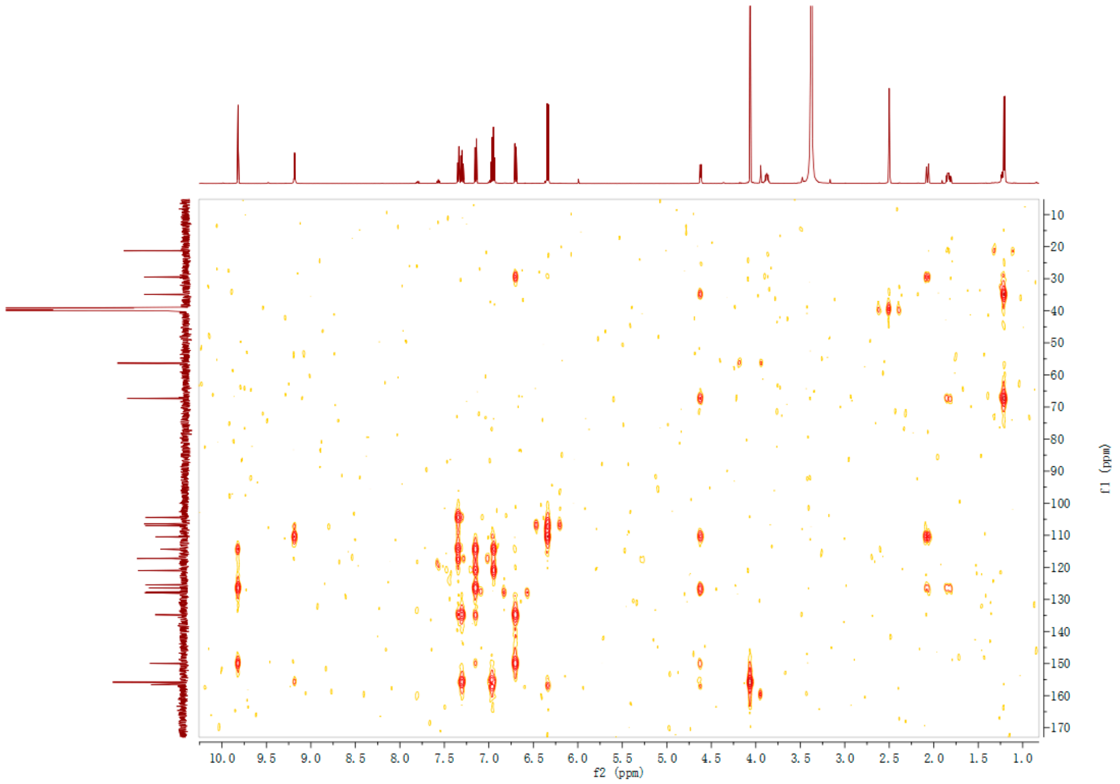

Figure S22. The HMBC spectrum of **3** in DMSO-*d*6

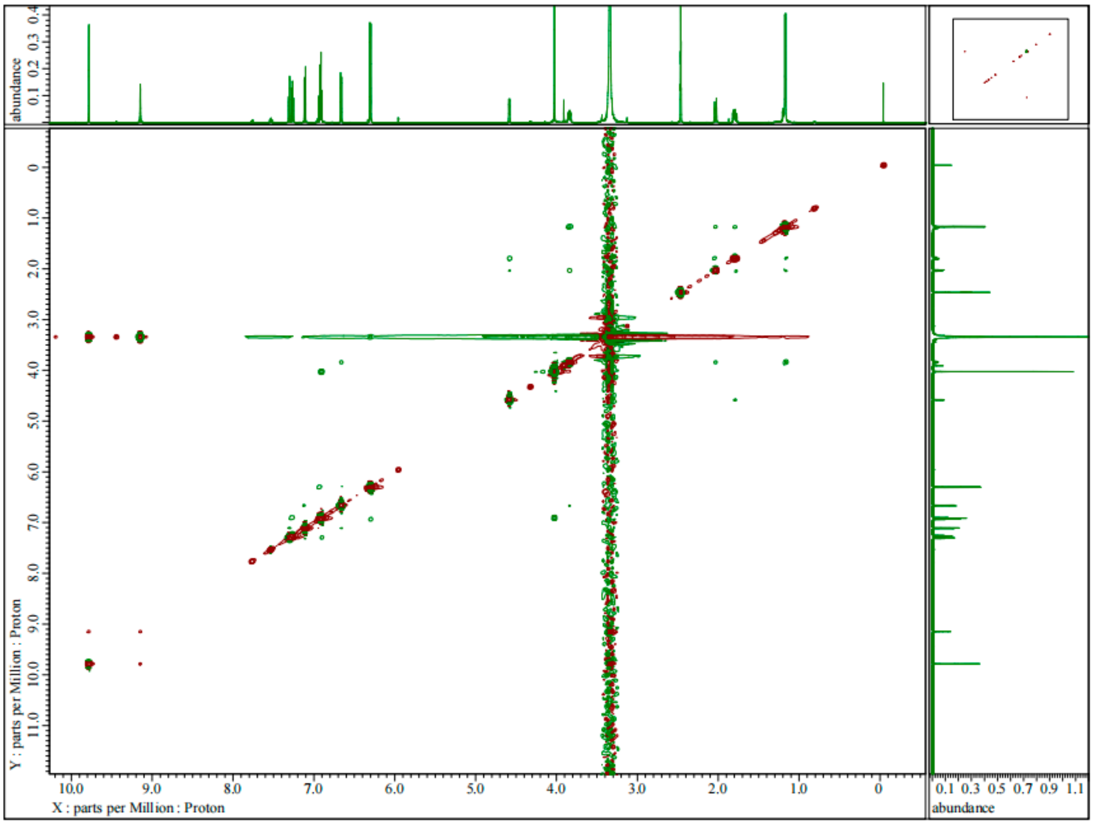

Figure S23. The ^{1}H-^{1}H NOESY spectrum of **3** in DMSO-d_6

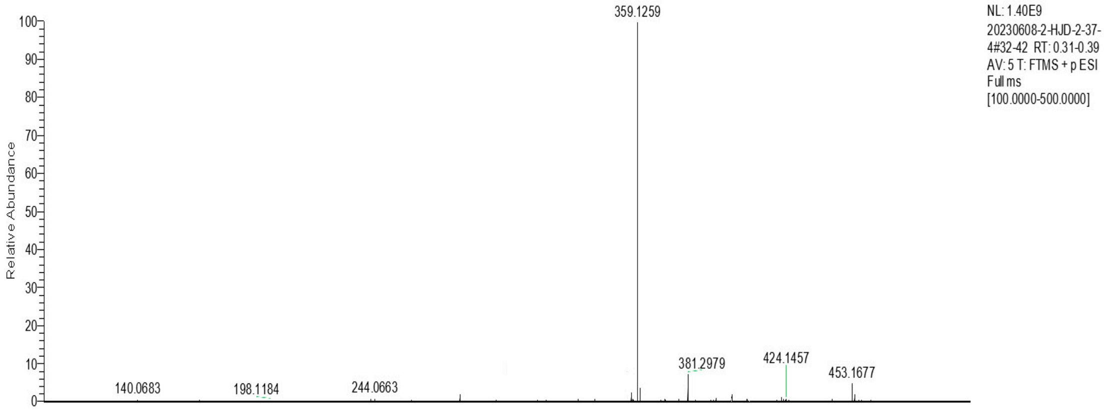

Figure S24. The HR-ESI-MS spectrum of **3** in CD_3OD

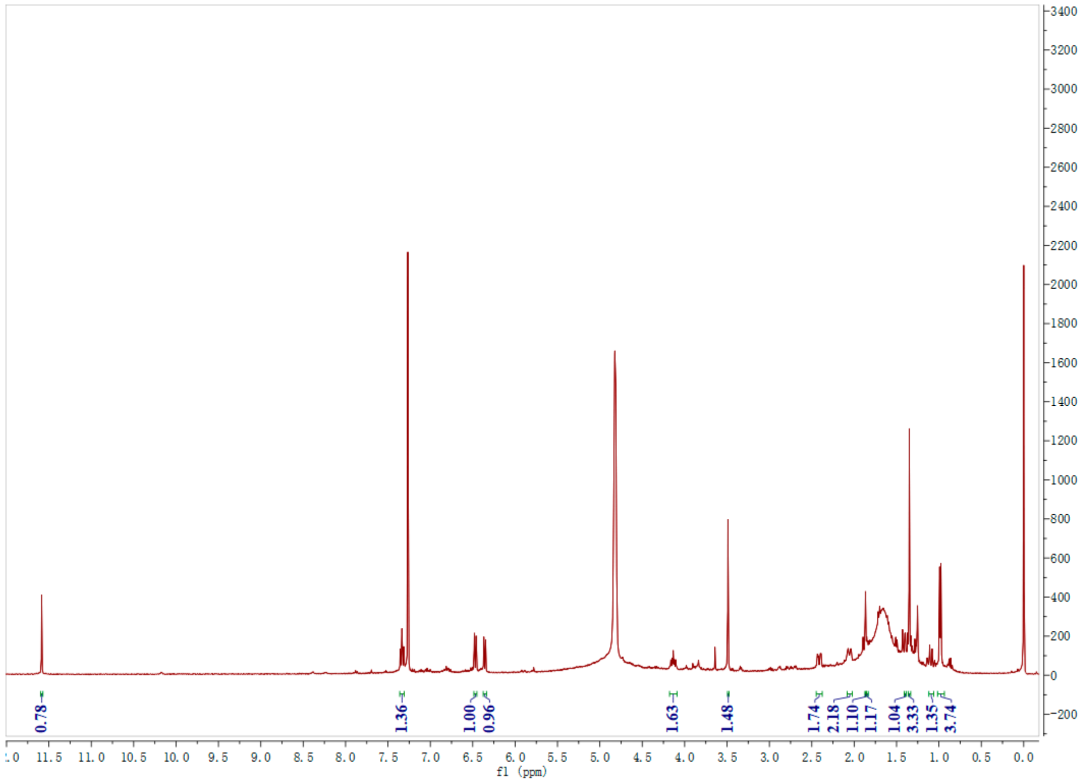

Figure S25. The ^{1}H NMR spectrum of **5** in $CDCl_3$

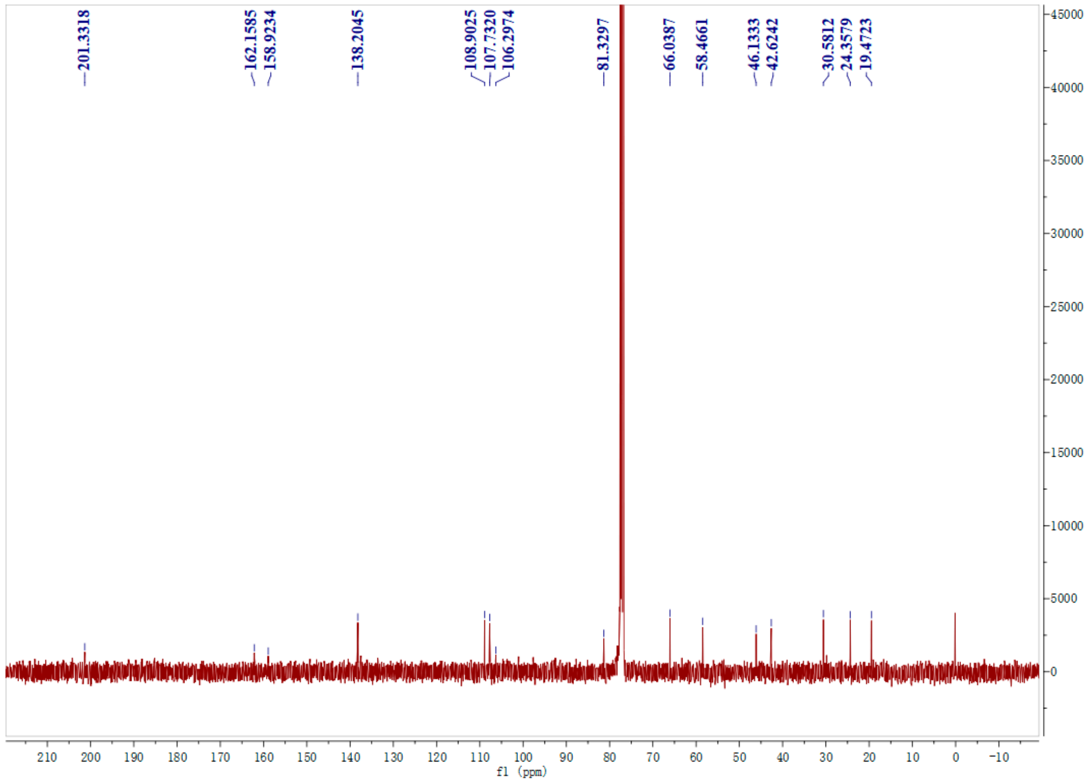

Figure S26. The ^{13}C NMR spectrum of **5** in $CDCl_3$

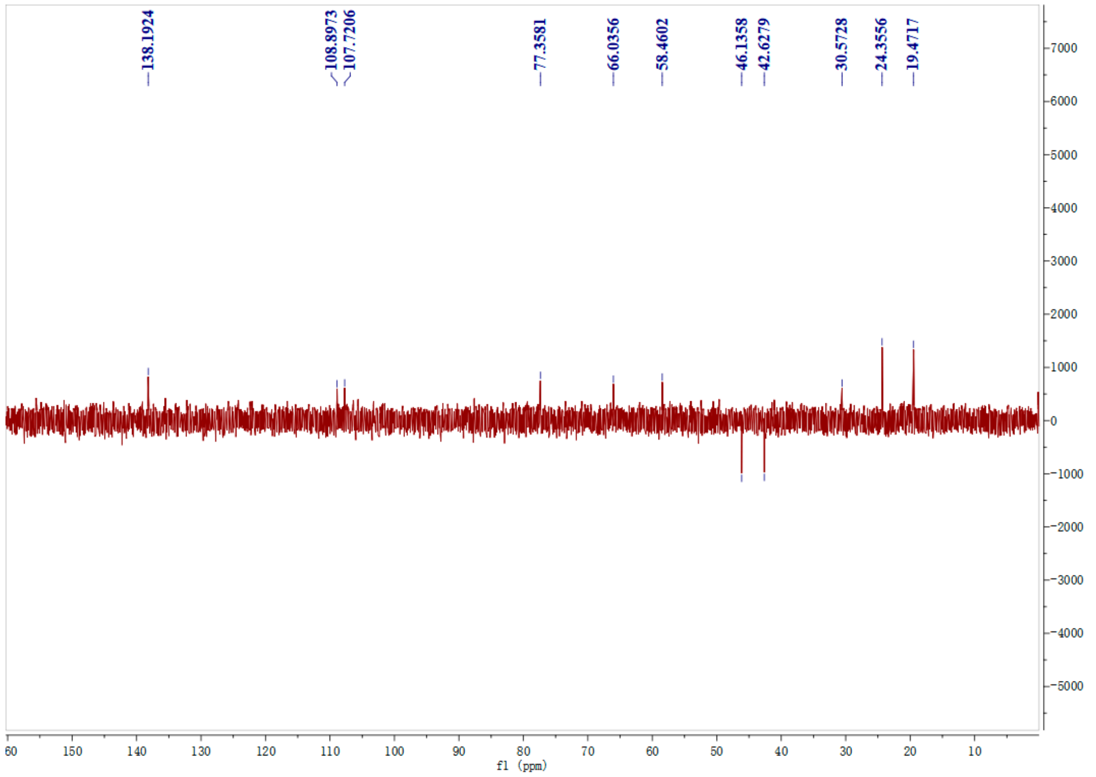

Figure S27. The DEPT-135 spectrum of **5** in $CDCl_3$

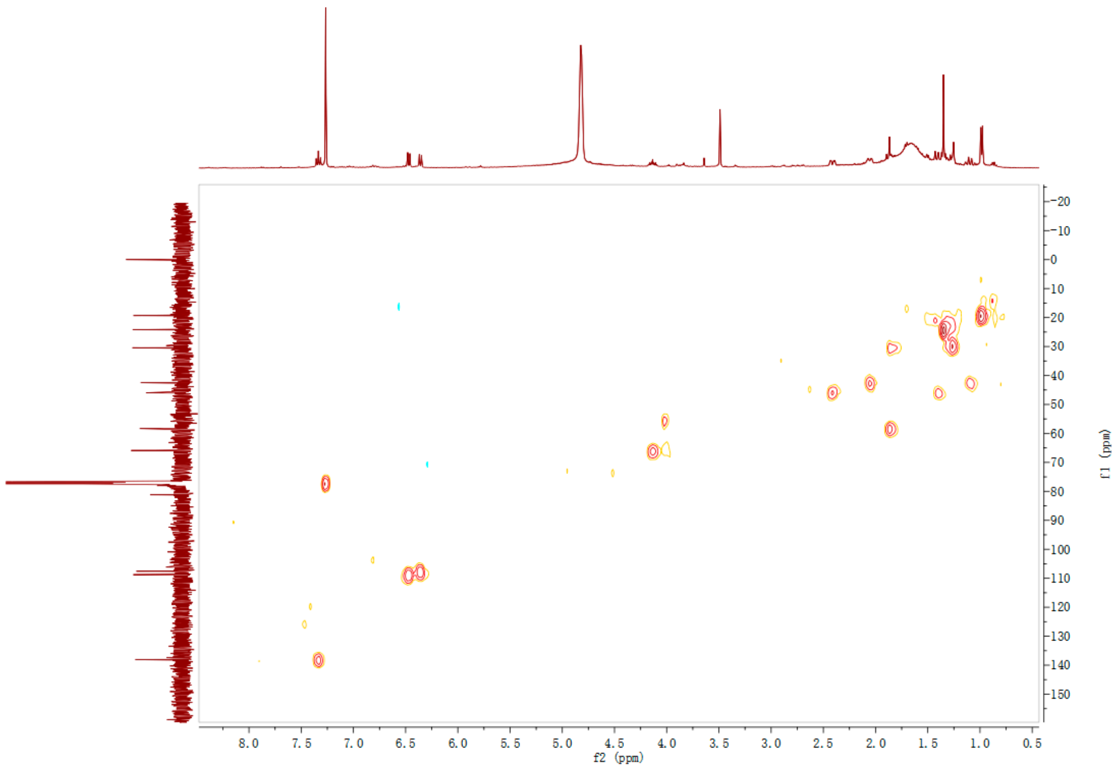

Figure S28. The HSQC spectrum of **5** in $CDCl_3$

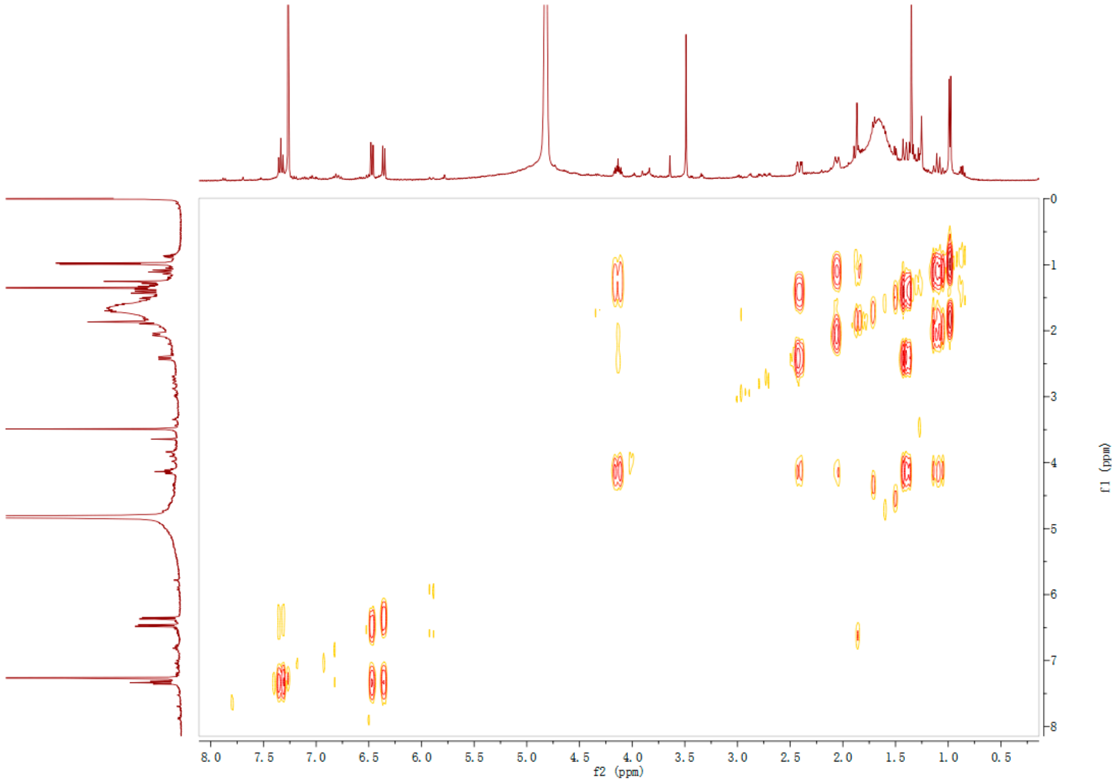

Figure S29. The ^{1}H-^{1}H COSY spectrum of **5** in CDCl3

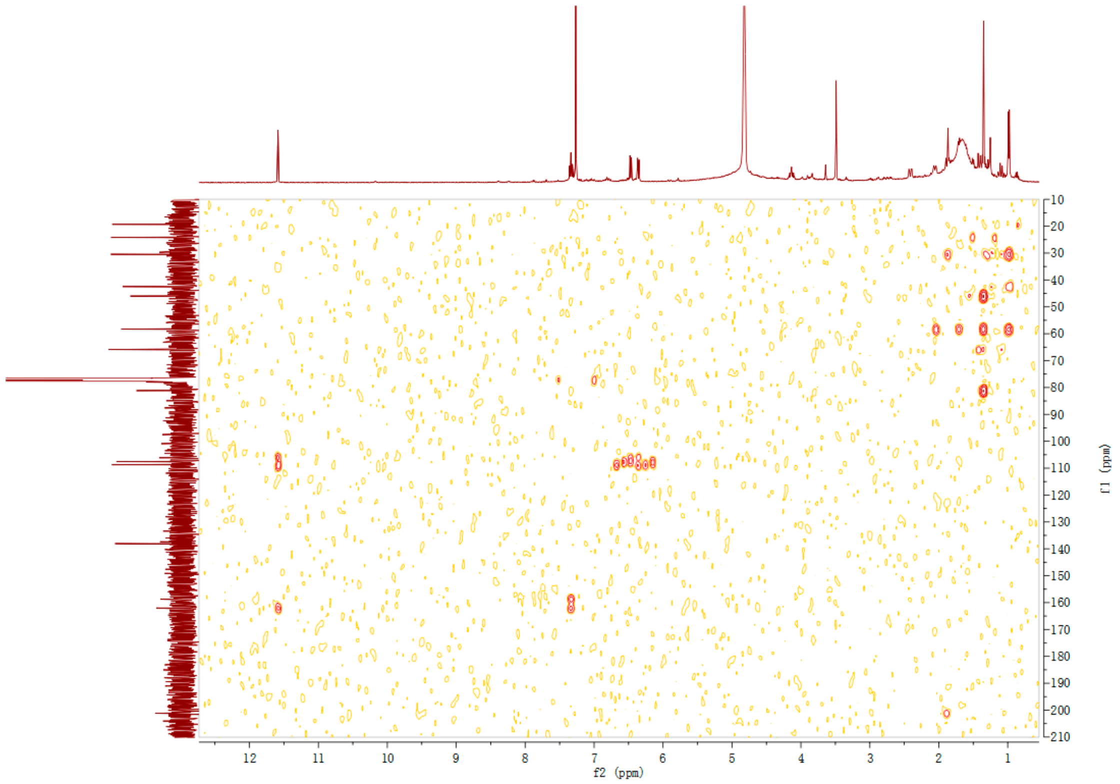

Figure S30. The HMBC spectrum of **5** in CDCl3

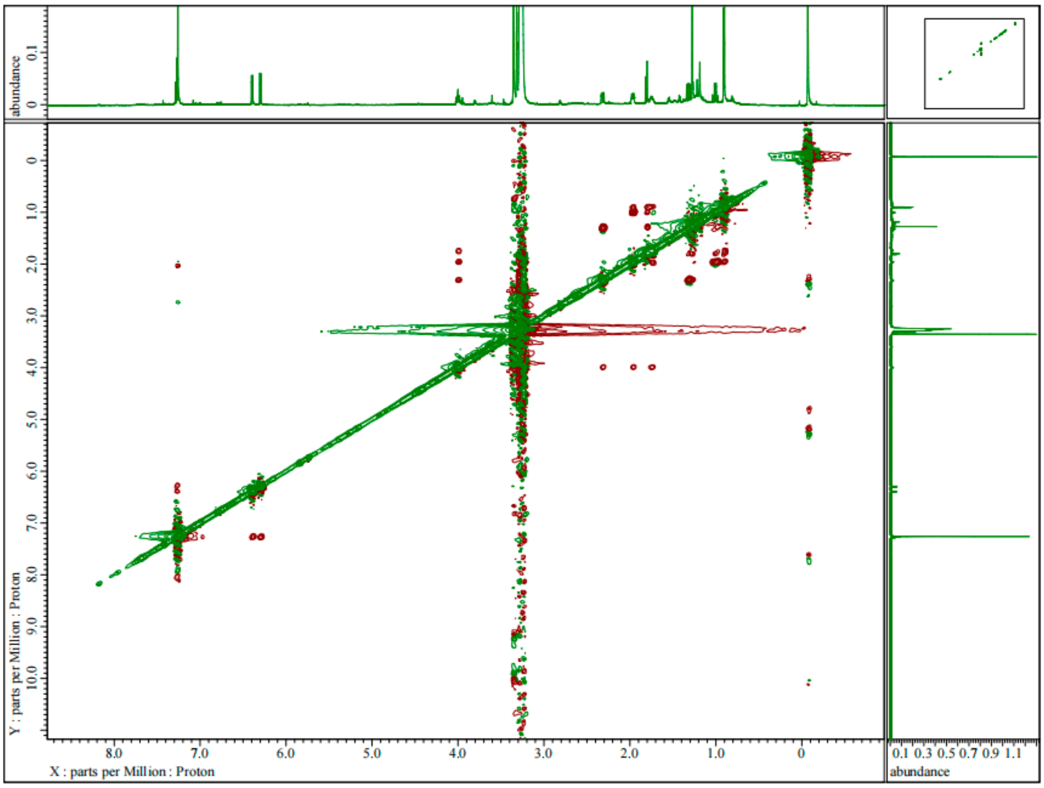

Figure S31. The ^{1}H-^{1}H NOESY spectrum of **5** in $CDCl_3$

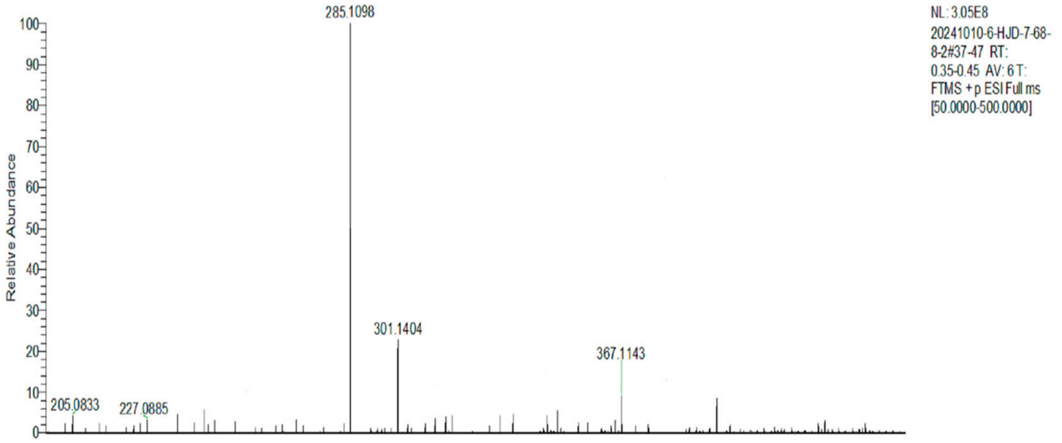

Figure S32. The HR-ESI-MS spectrum of **5** in CD_3OD

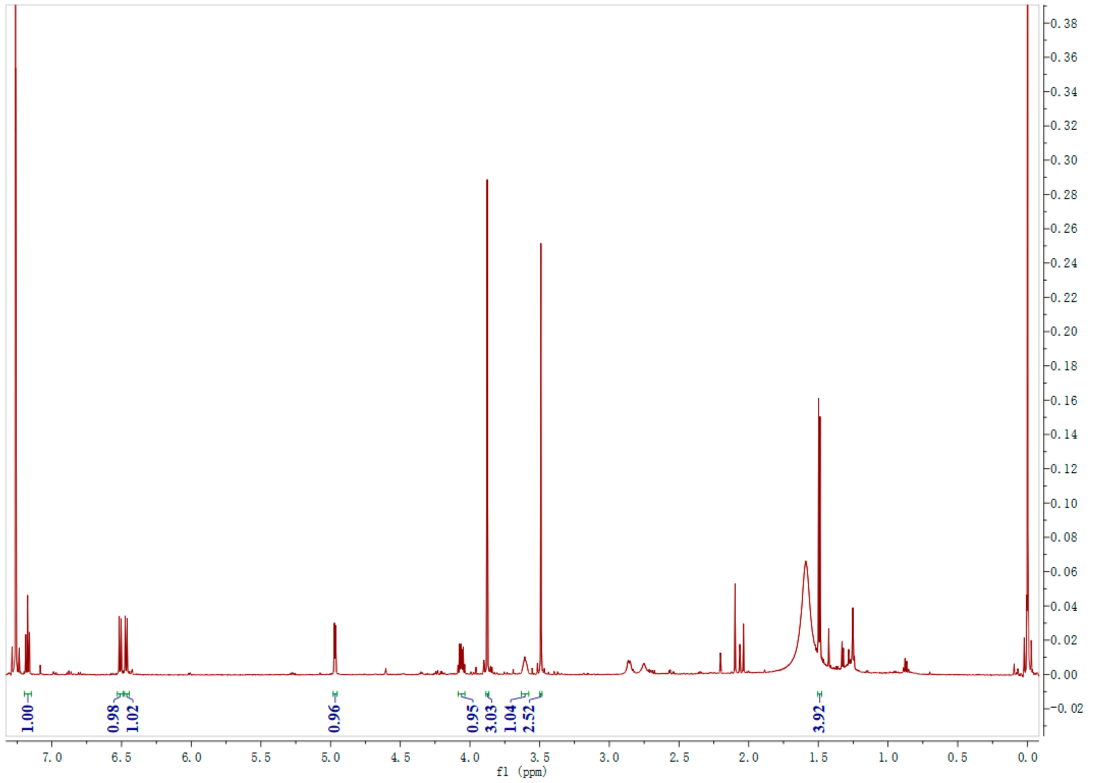

Figure S33. The ^{1}H NMR spectrum of **6** in $CDCl_3$

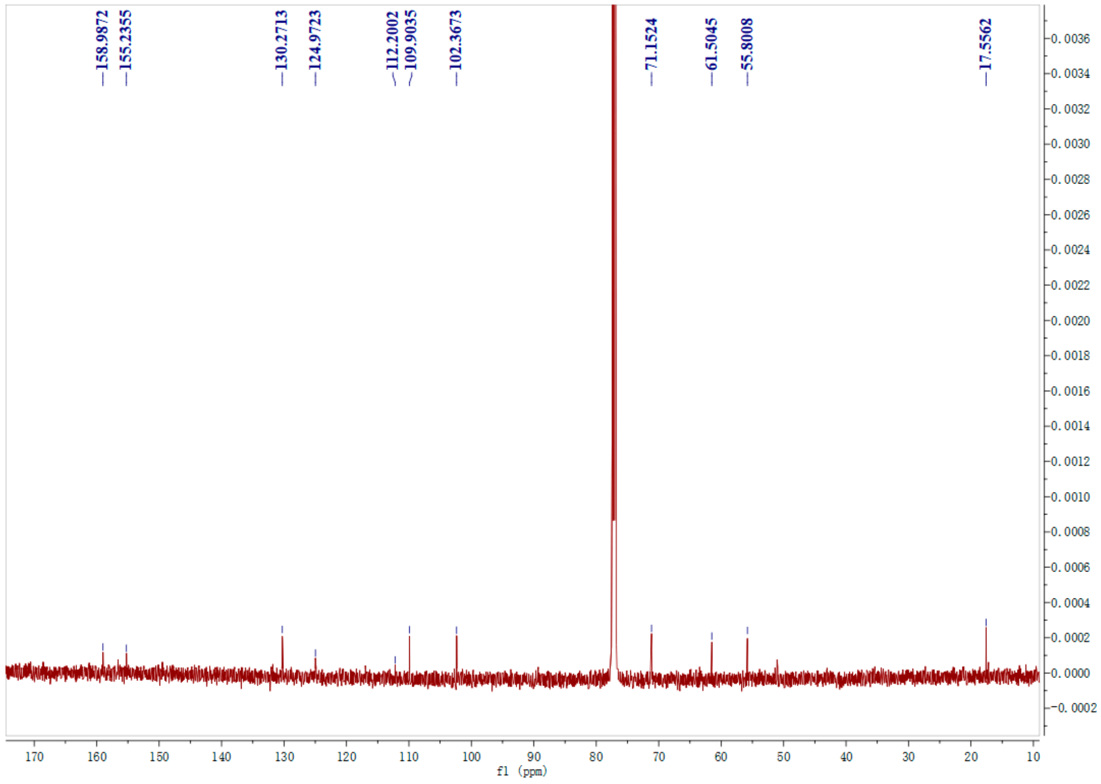

Figure S34. The ^{13}C NMR spectrum of **6** in $CDCl_3$

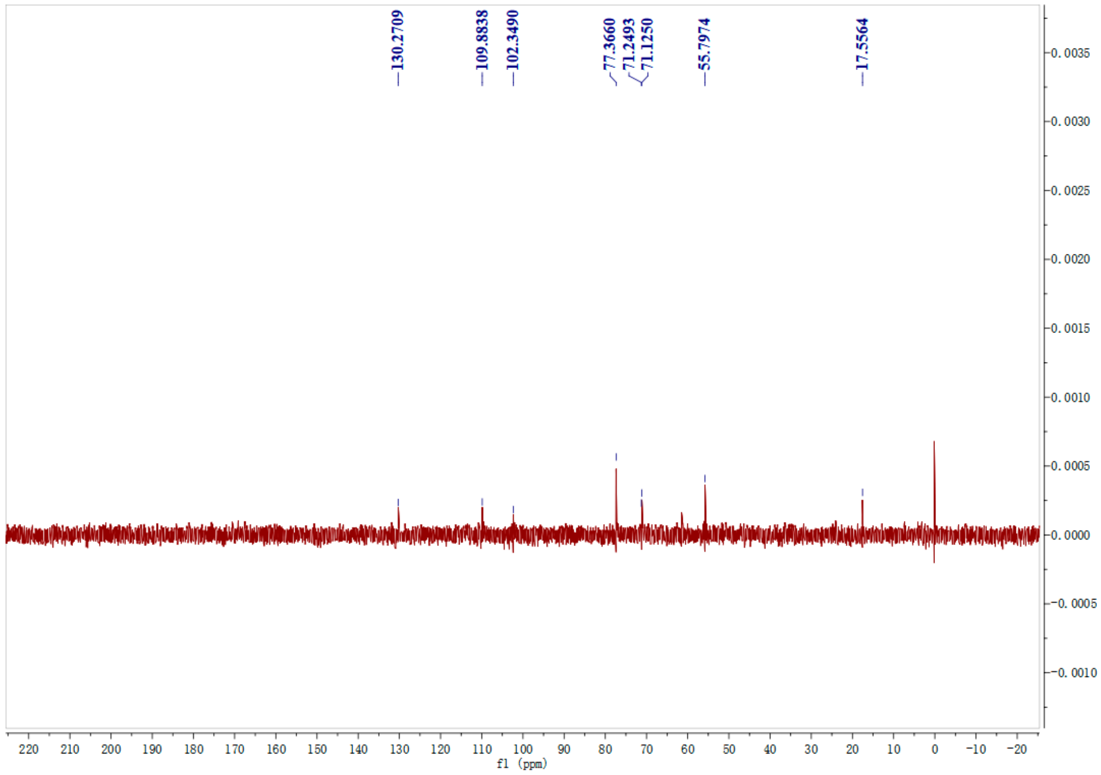

Figure S35. The DEPT-135 spectrum of **6** in $CDCl_3$

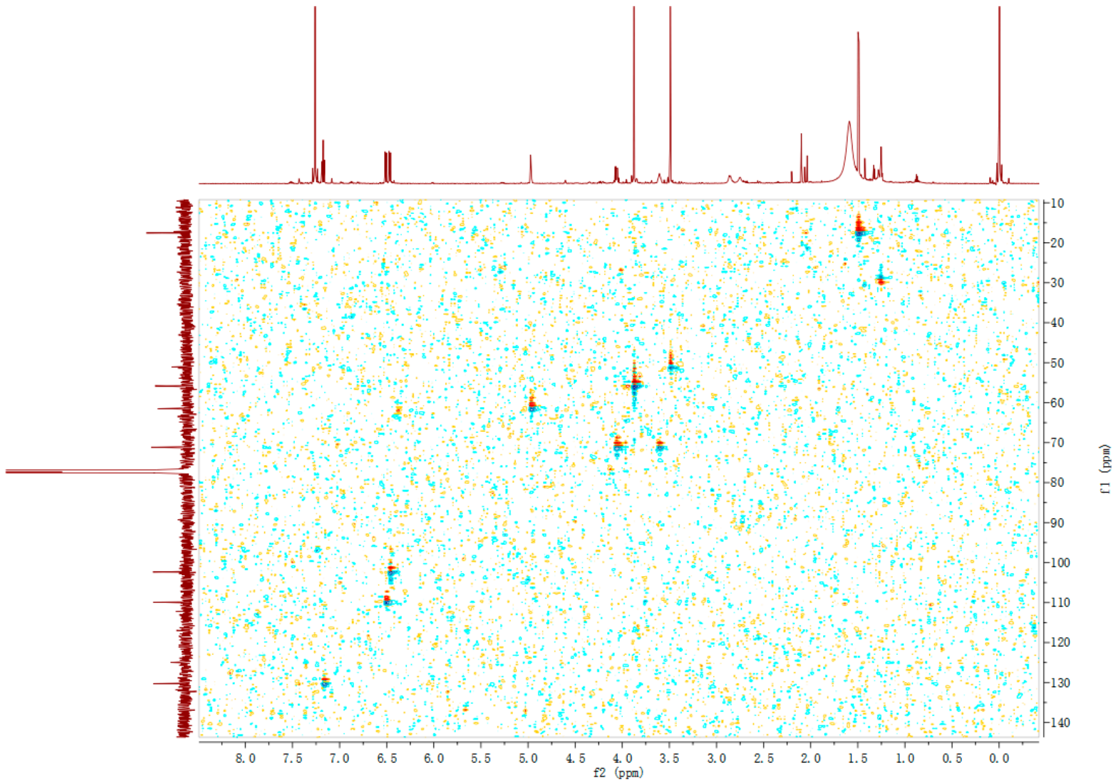

Figure S36. The HSQC spectrum of **6** in $CDCl_3$

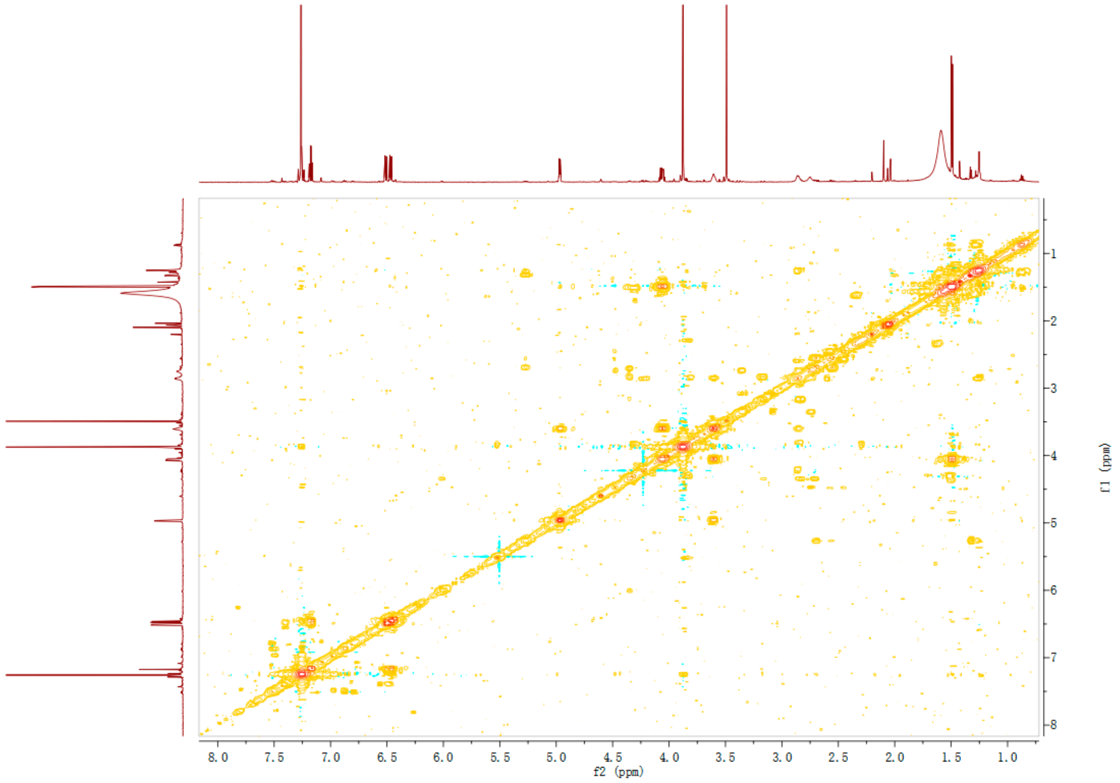

Figure S37. The ^{1}H-^{1}H COSY spectrum of **6** in $CDCl_3$

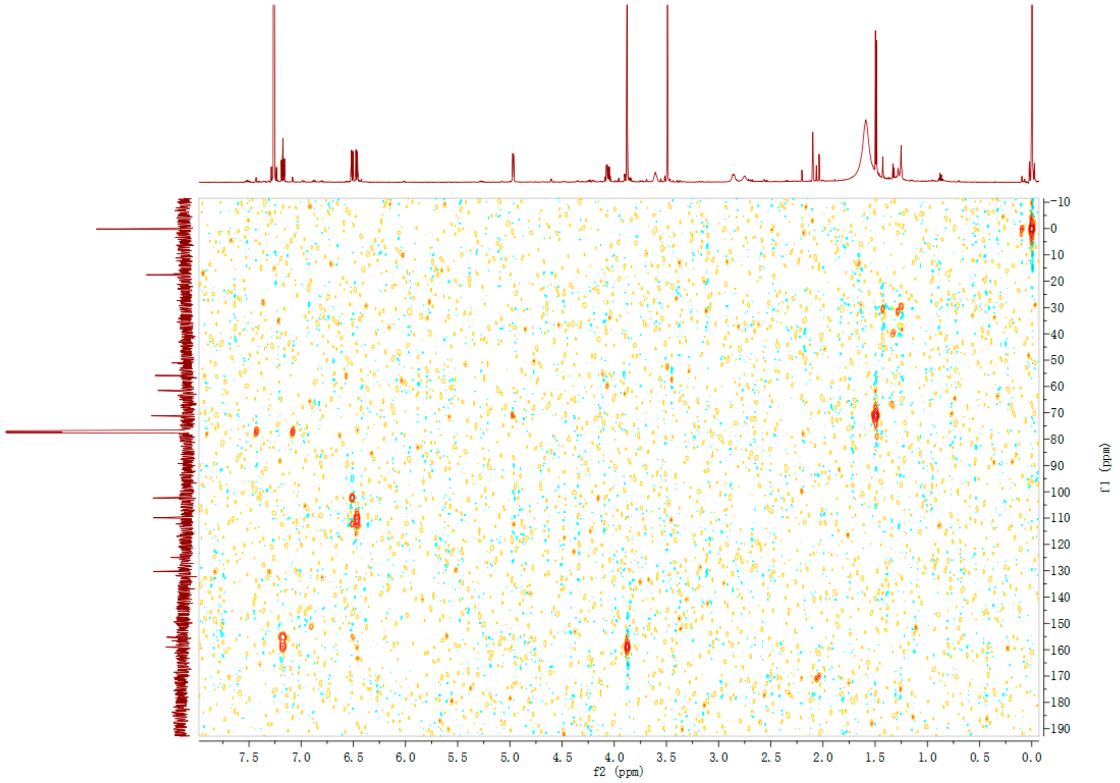

Figure S38. The HMBC spectrum of **6** in $CDCl_3$

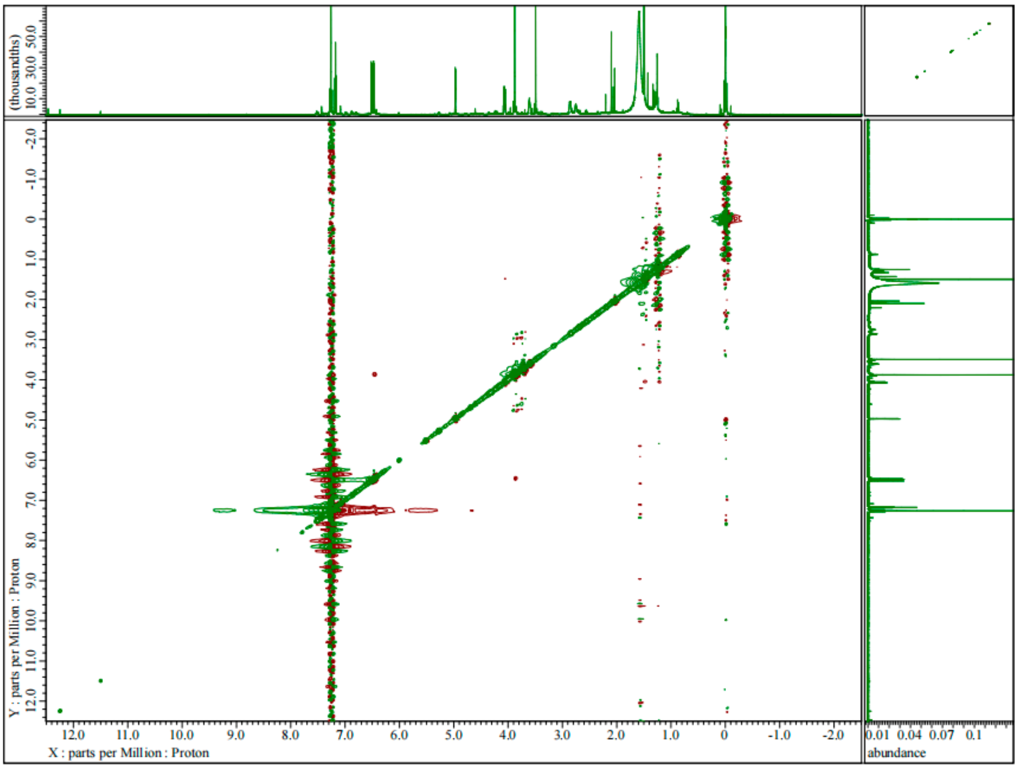

Figure S39. The ^{1}H-^{1}H NOESY spectrum of **6** in $CDCl_3$

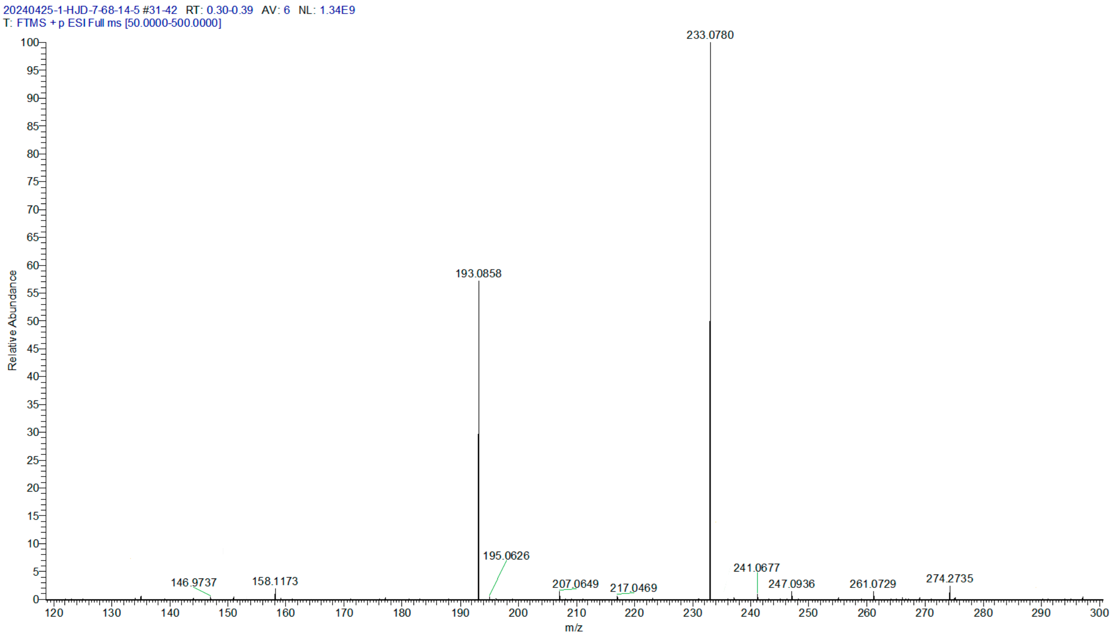

Figure S40. The HR-ESI-MS spectrum of **6** in CD_3OD

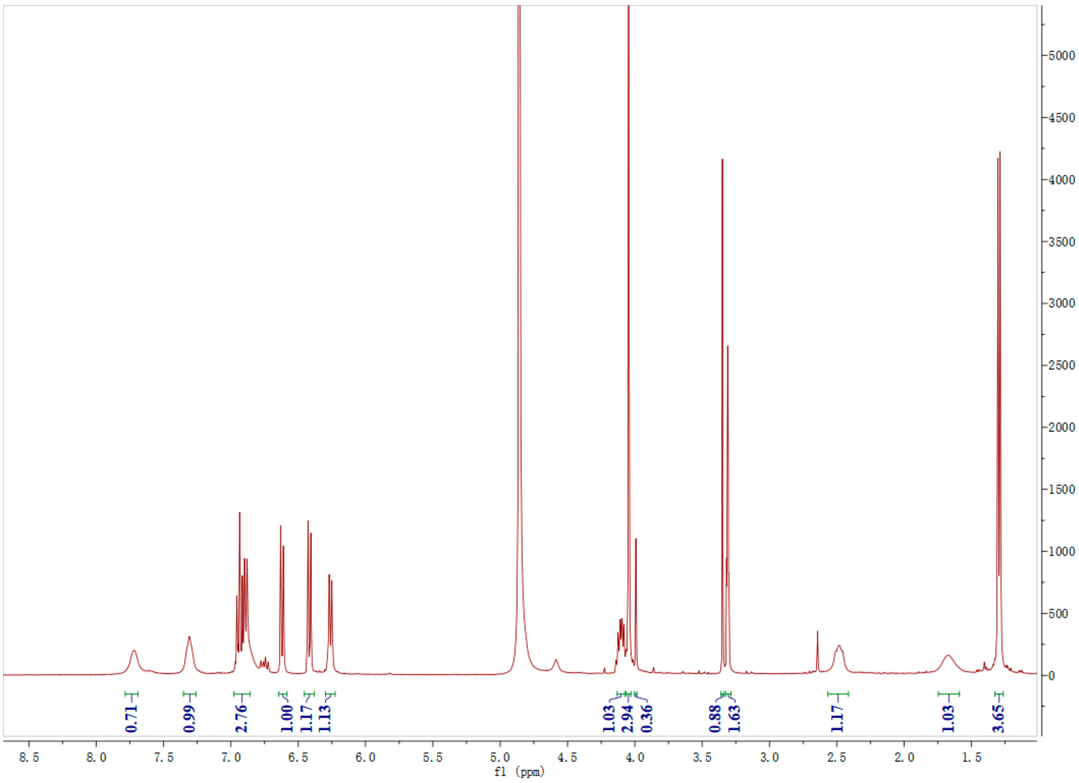

Figure S41. The ^{1}H NMR spectrum of **2** in CD_3OD-d_4

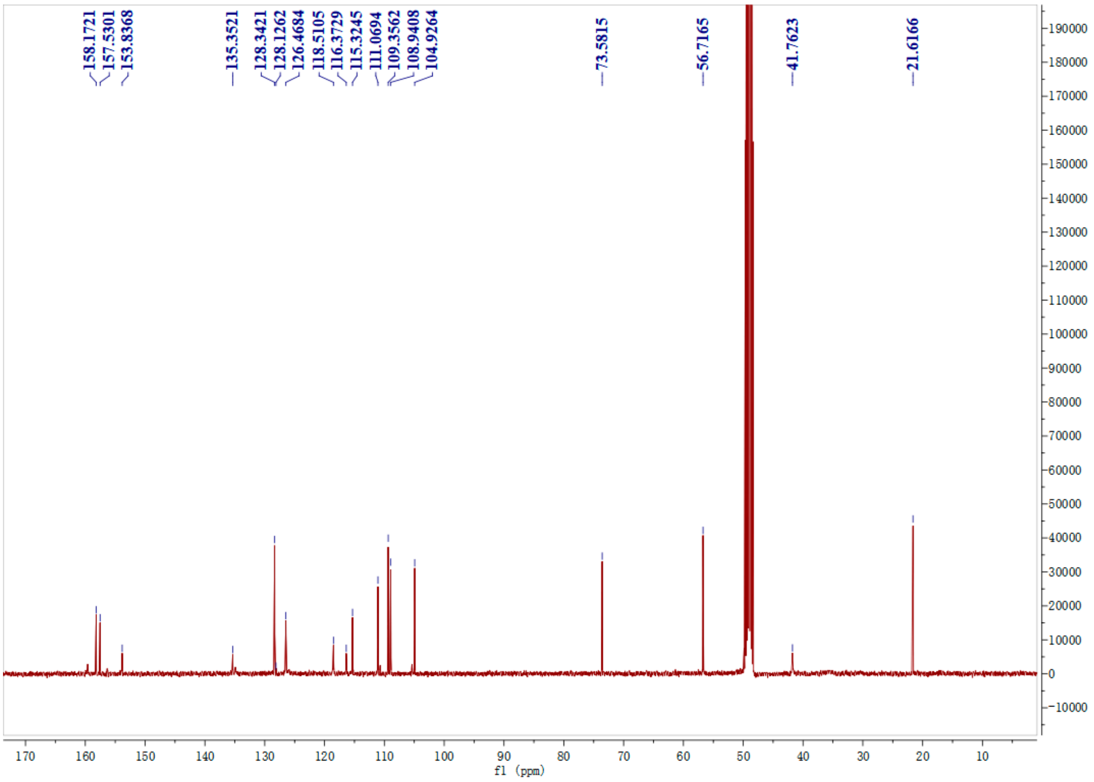

Figure S42. The ^{13}C NMR spectrum of **2** in CD_3OD-d_4

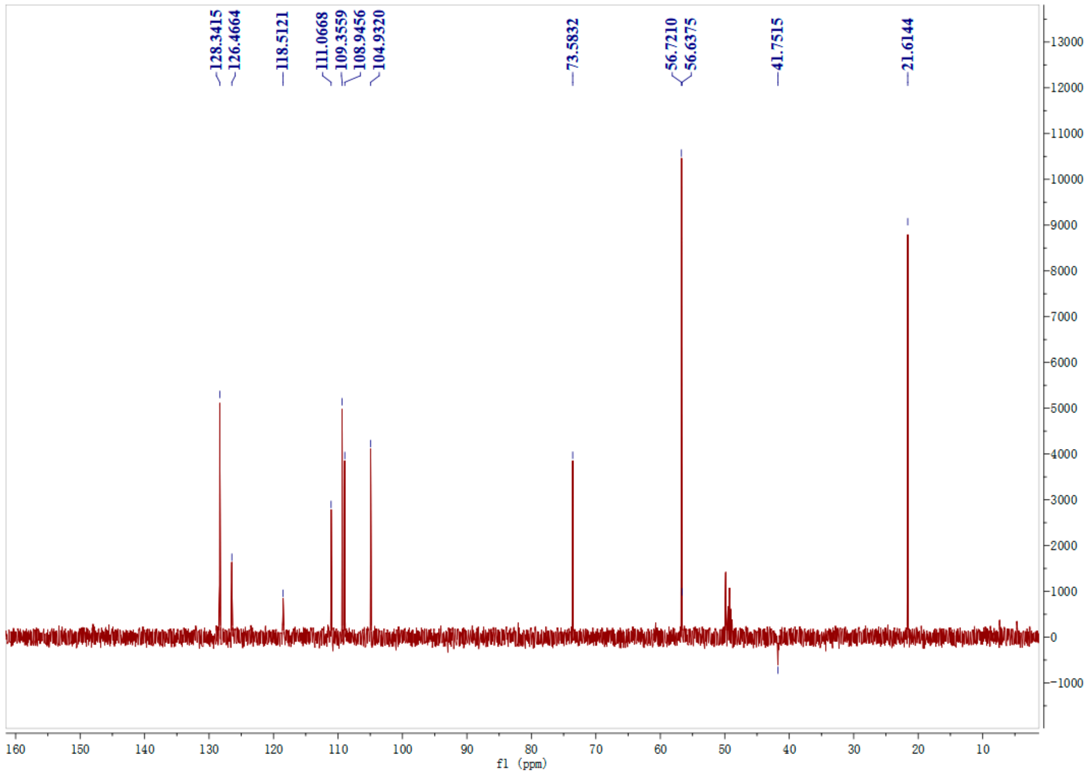

Figure S43. The DEPT-135 spectrum of **2** in CD_3OD-d_4

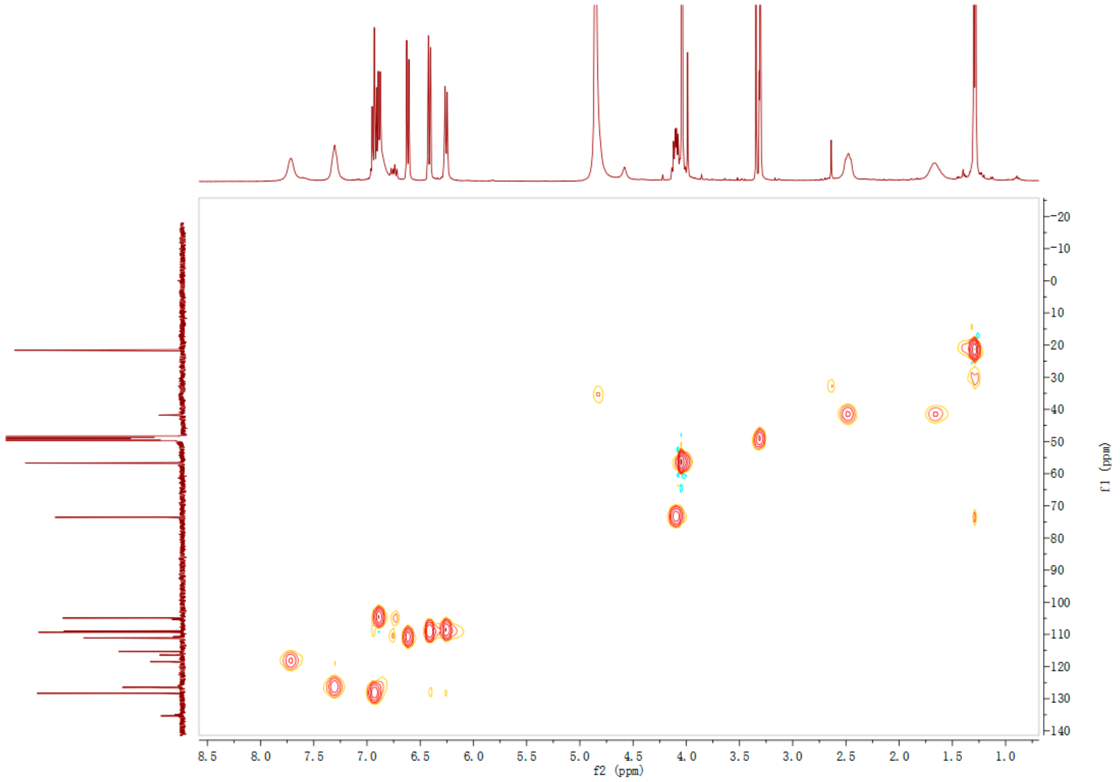

Figure S44. The HSQC spectrum of **2** in CD_3OD-d_4

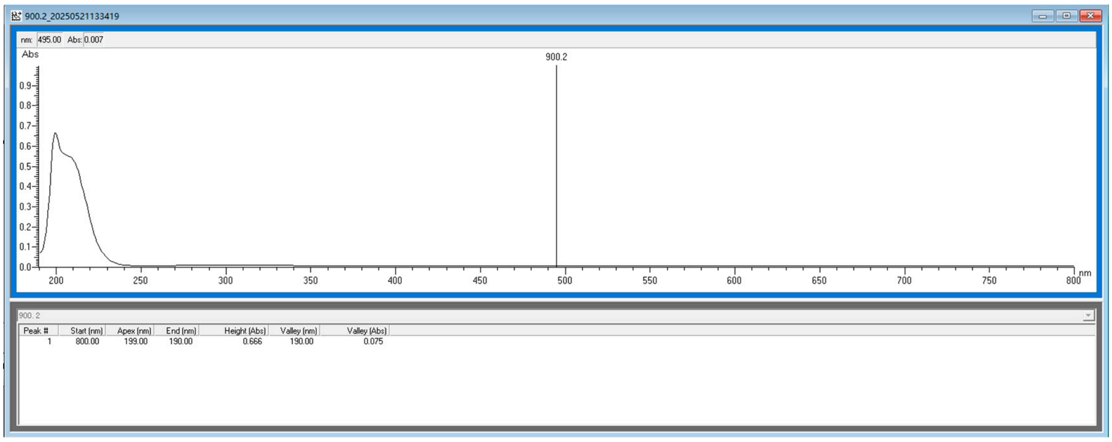

Figure S45. The UV spectrum of **1**

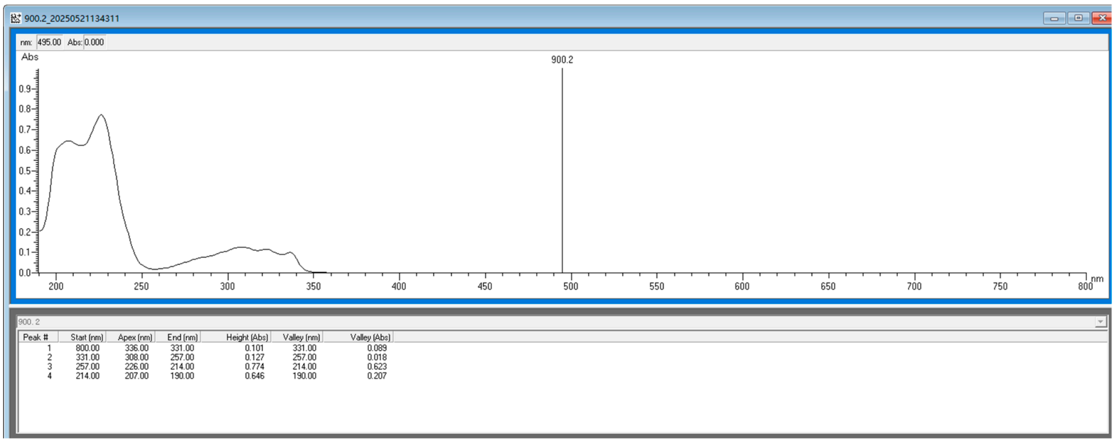

Figure S46. The UV spectrum of **2**

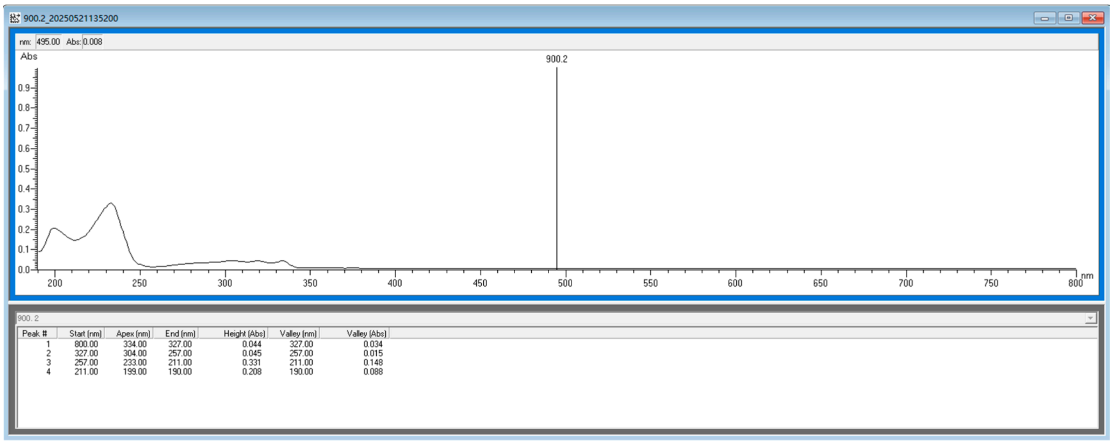

Figure S47. The UV spectrum of **3**

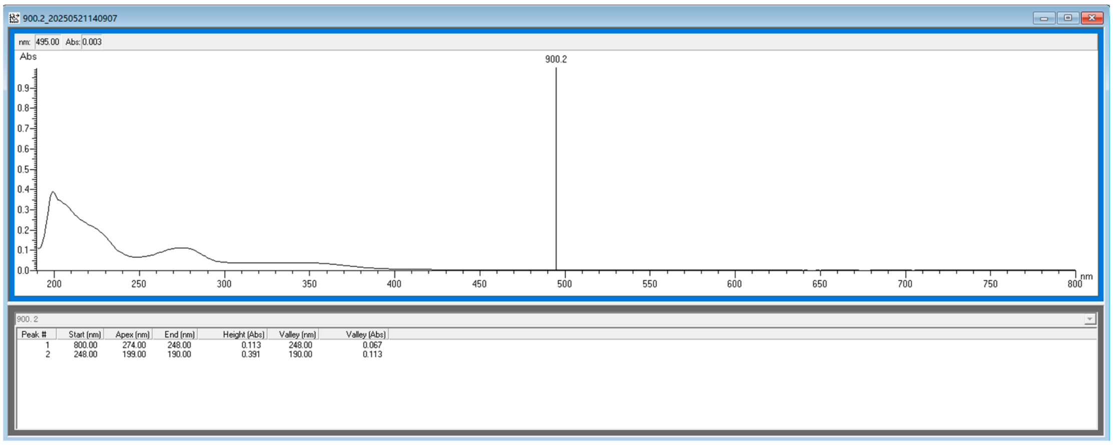

Figure S48. The UV spectrum of **5**

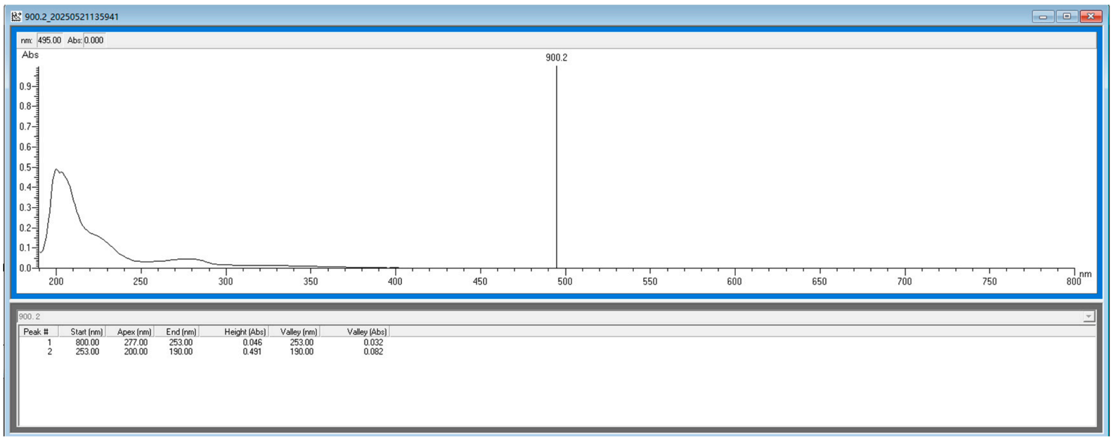

Figure S49. The UV spectrum of **6**

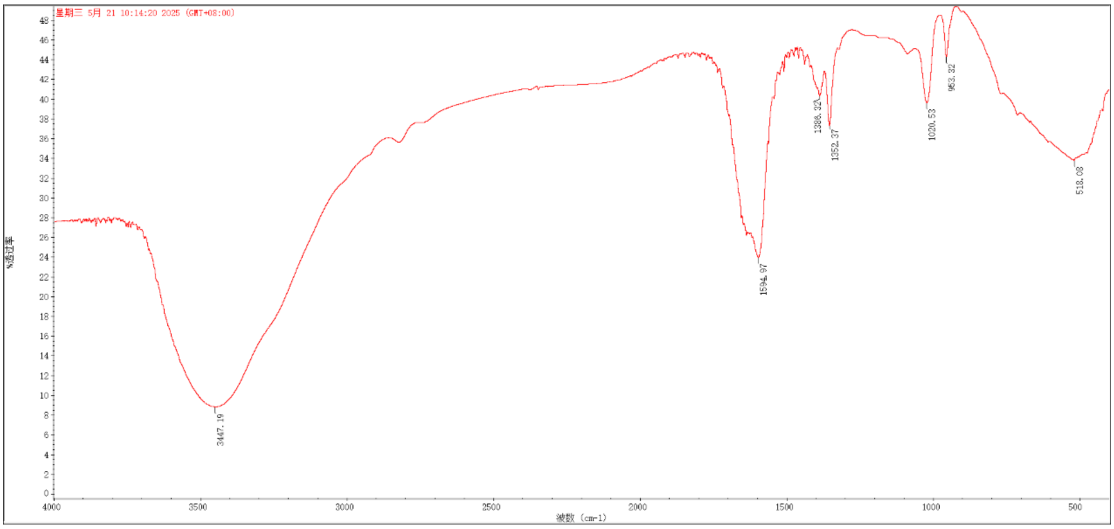

Figure S50. The IR spectrum of **1**

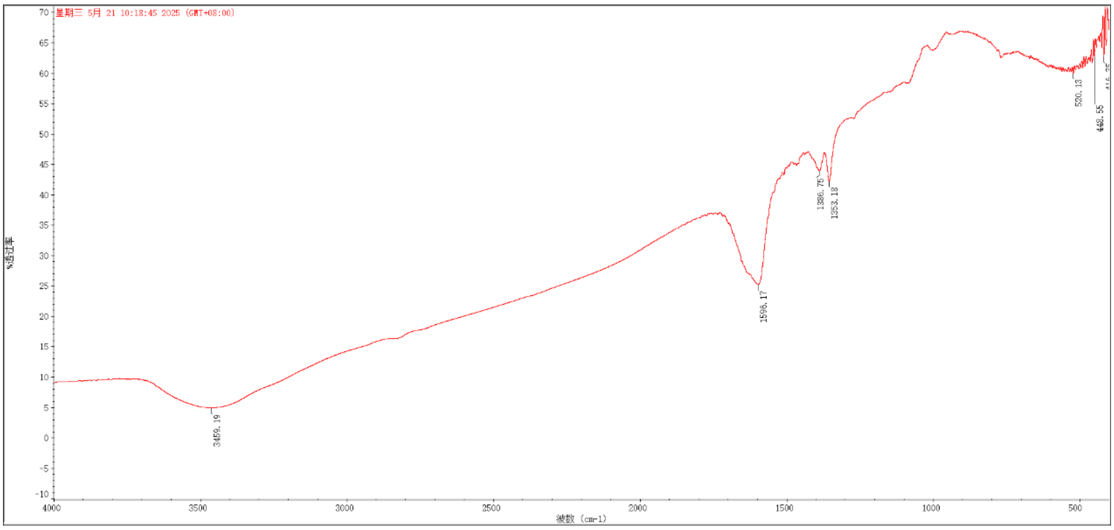

Figure S51. The IR spectrum of **2**

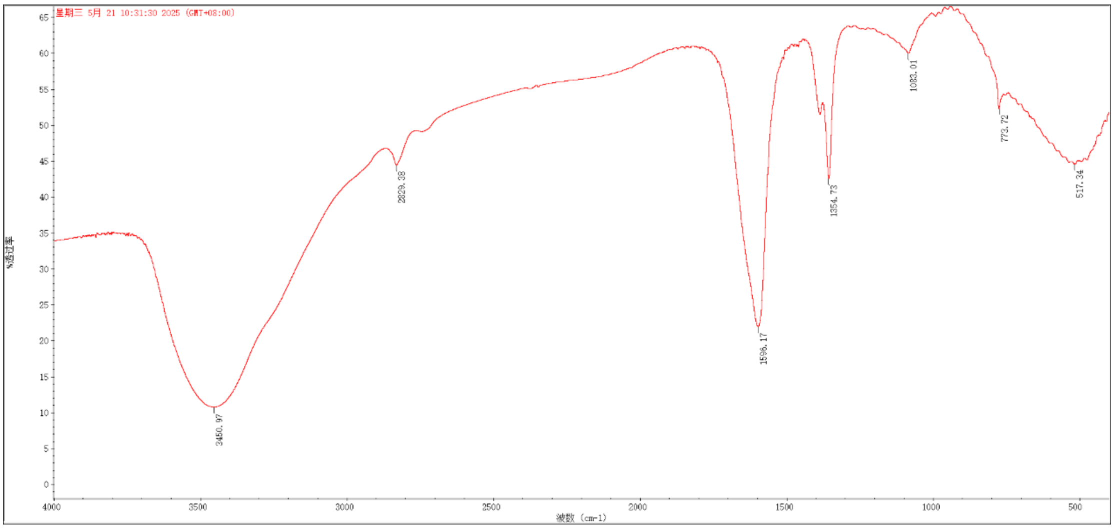

Figure S52. The IR spectrum of **3**

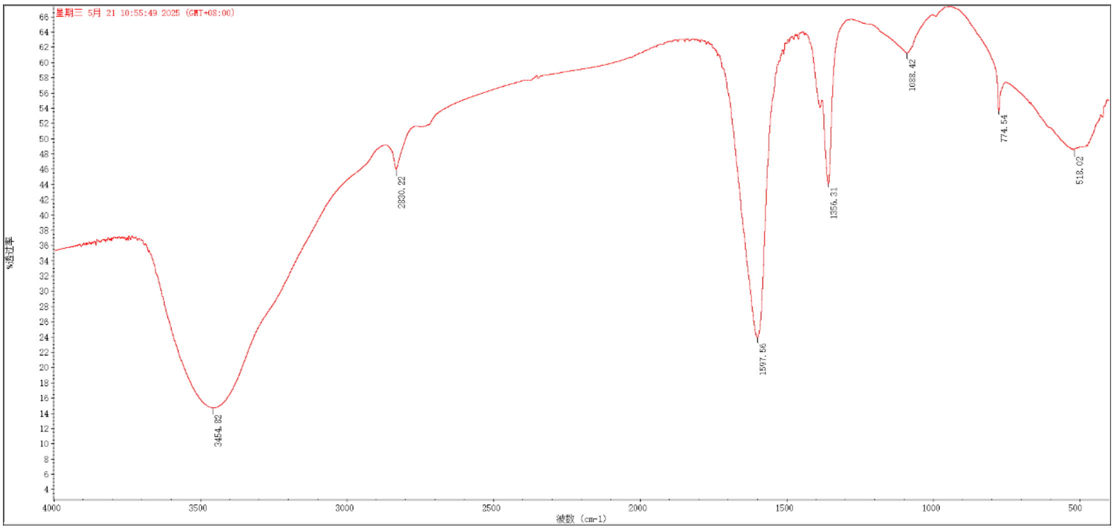

Figure S53. The IR spectrum of **5**

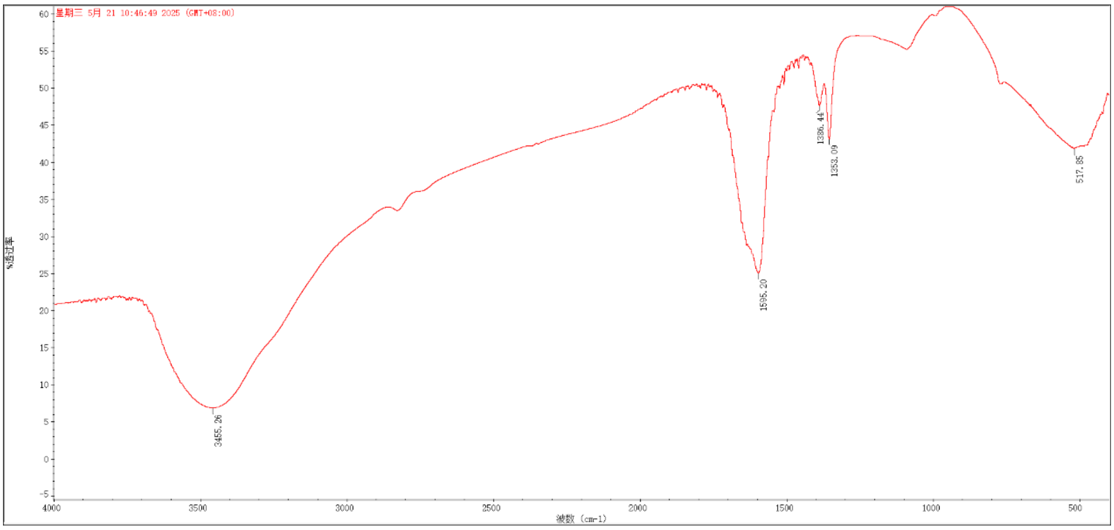

Figure S54. The IR spectrum of **6**

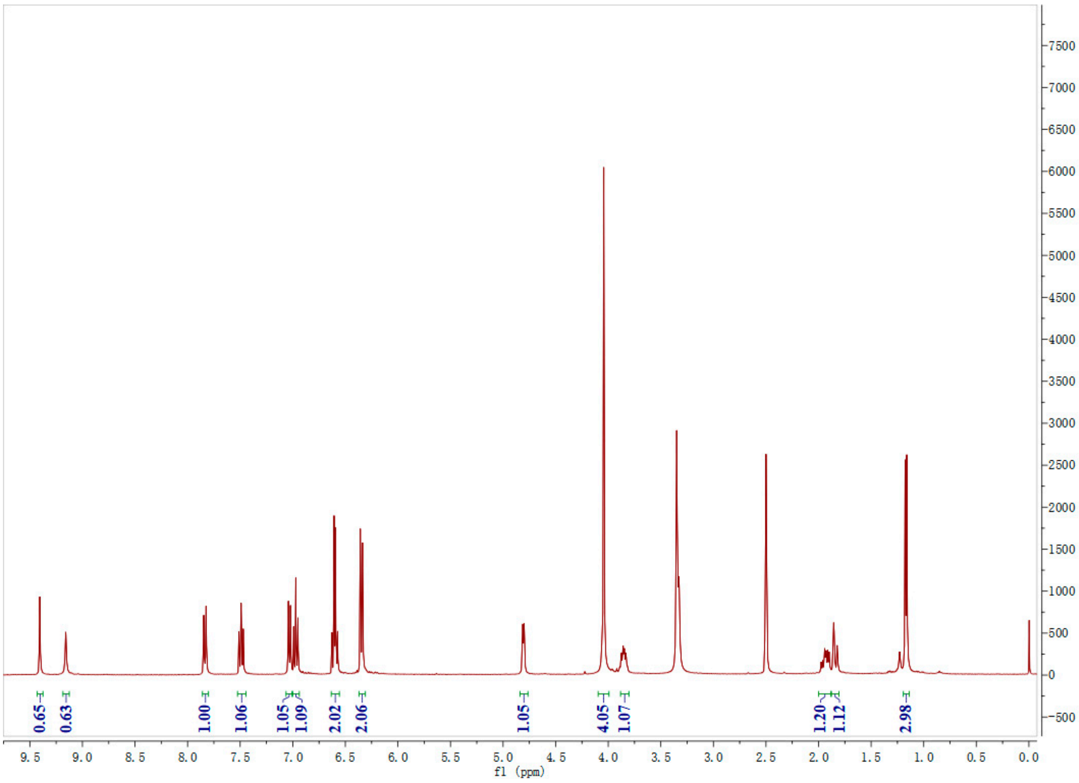

Figure S55. The ^{1}H NMR spectrum of **4** in DMSO-d_6

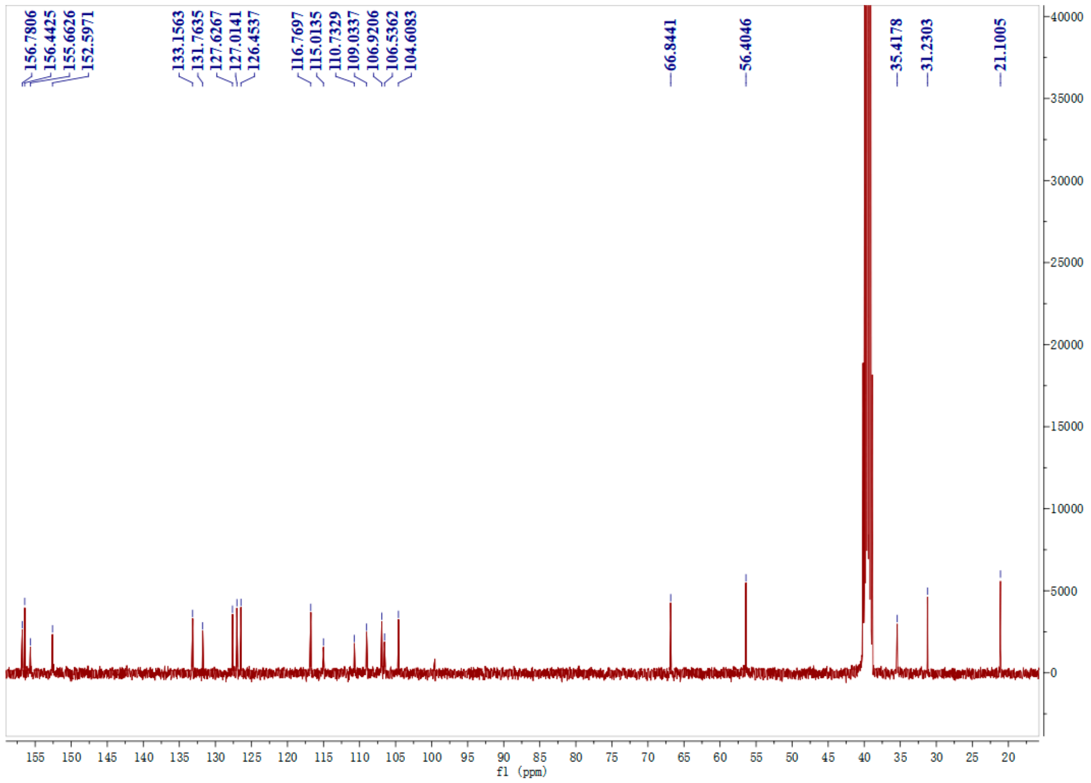

Figure S56. The ^{13}C NMR spectrum of **4** in DMSO-d_6

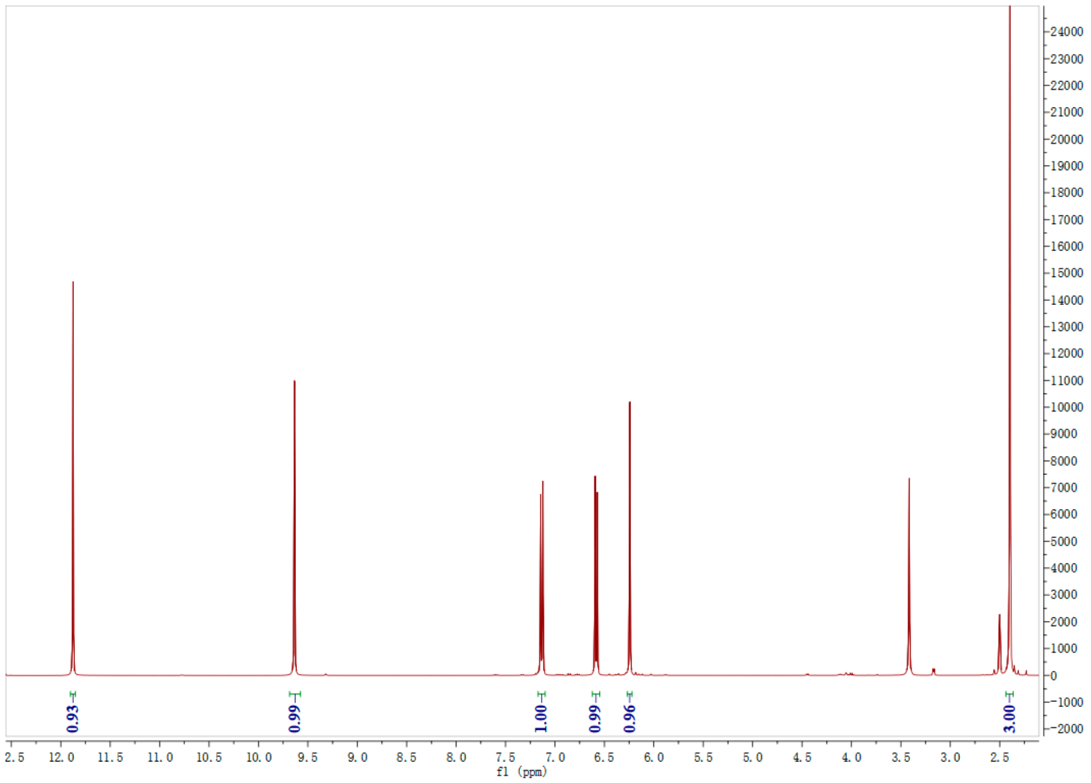

Figure S57. The ^{1}H NMR spectrum of **7** in DMSO-d_6

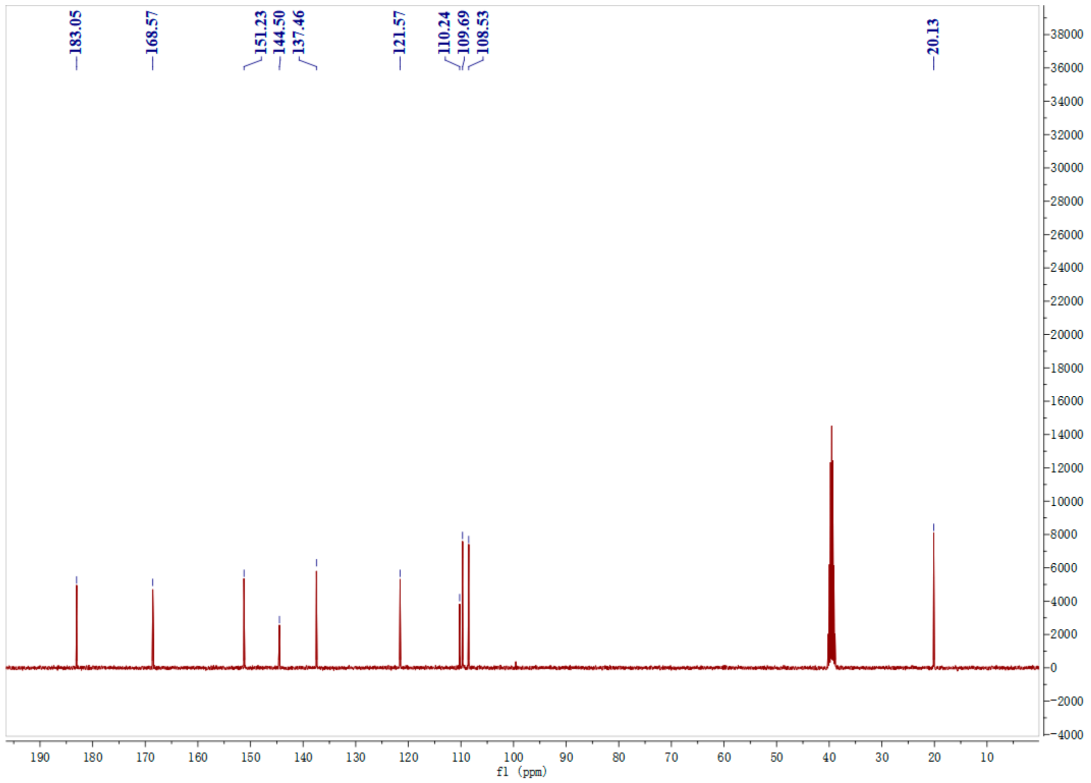

Figure S58. The ^{13}C NMR spectrum of **7** in DMSO-d_6

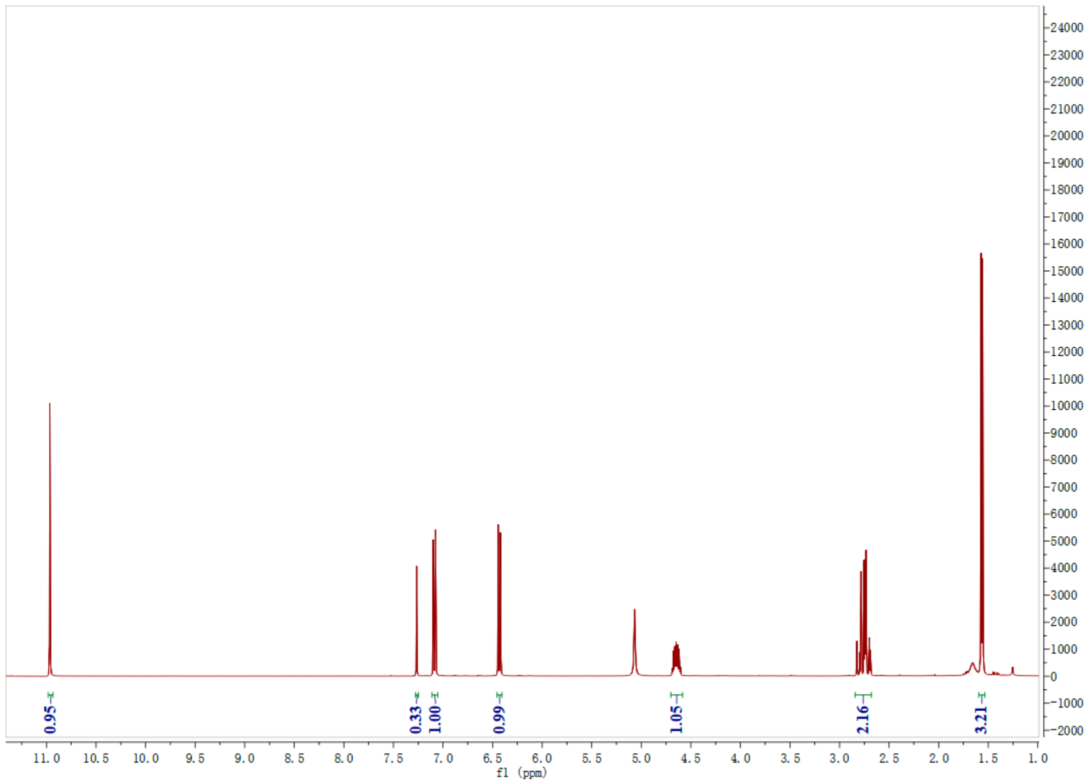

Figure S59. The ^{1}H NMR spectrum of **8** in $CDCl_3$

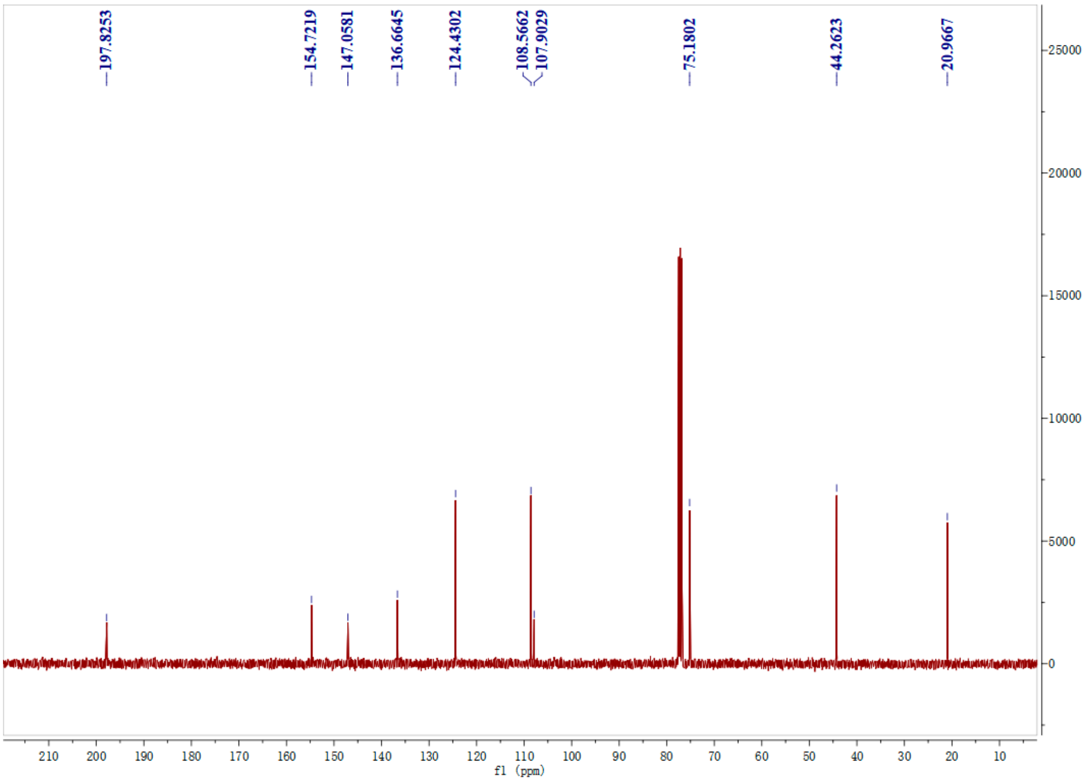

Figure S60. The ^{13}C NMR spectrum of **8** in $CDCl_3$

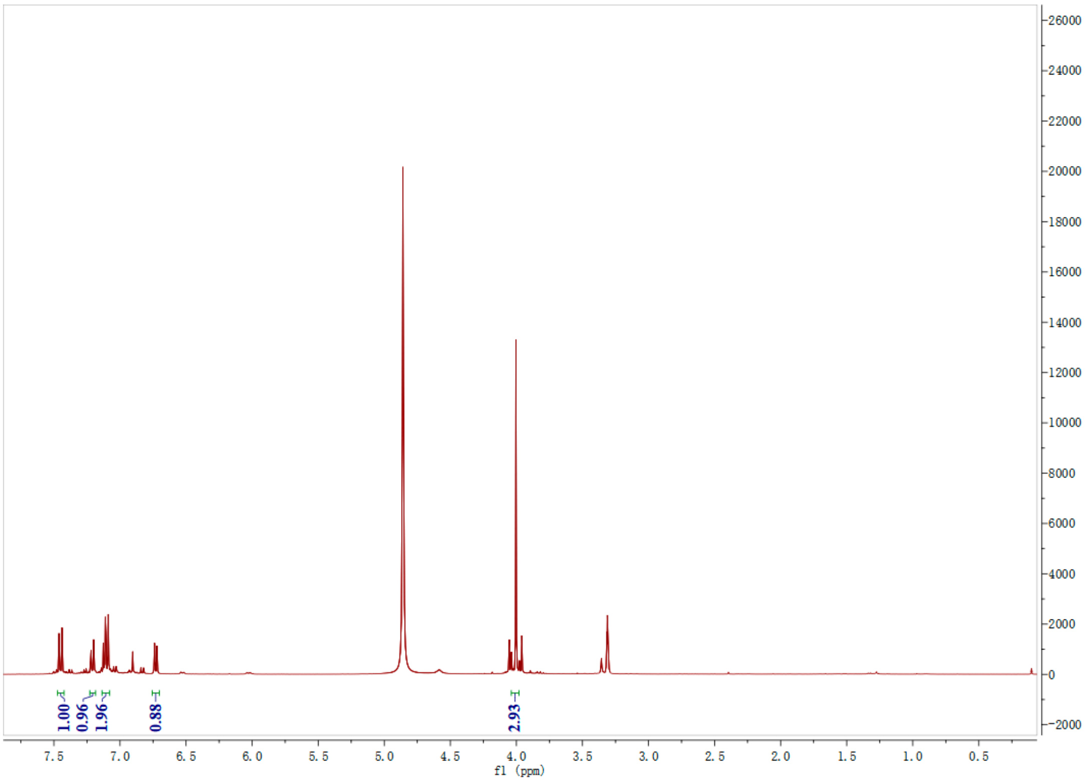

Figure S61. The ^{1}H NMR spectrum of **9** in CD_3OD

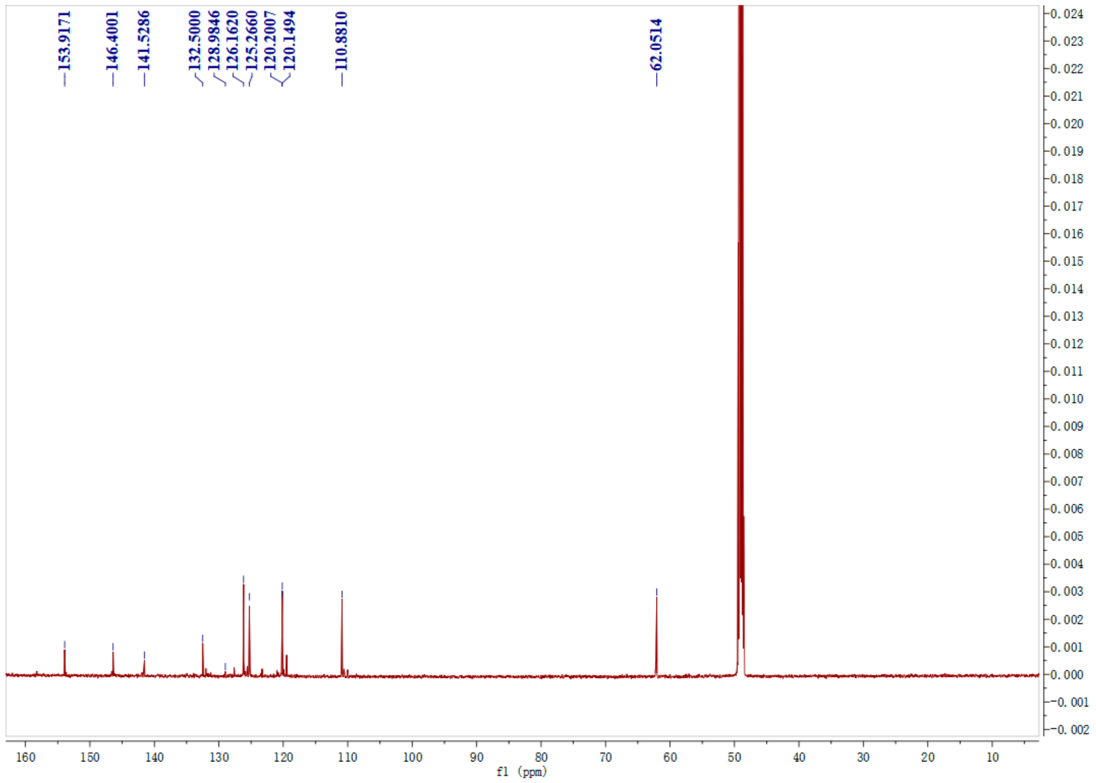

Figure S62. The ^{13}C NMR spectrum of **9** in CD_3OD

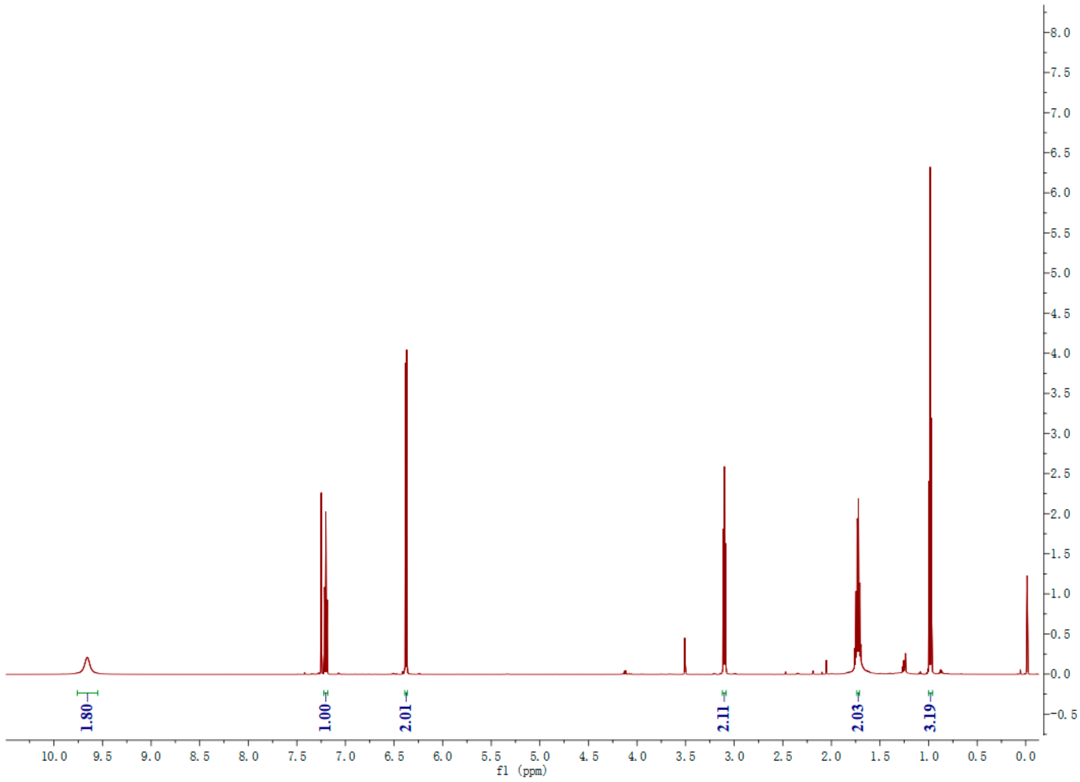

Figure S63. The ^{1}H NMR spectrum of **10** in $CDCl_3$

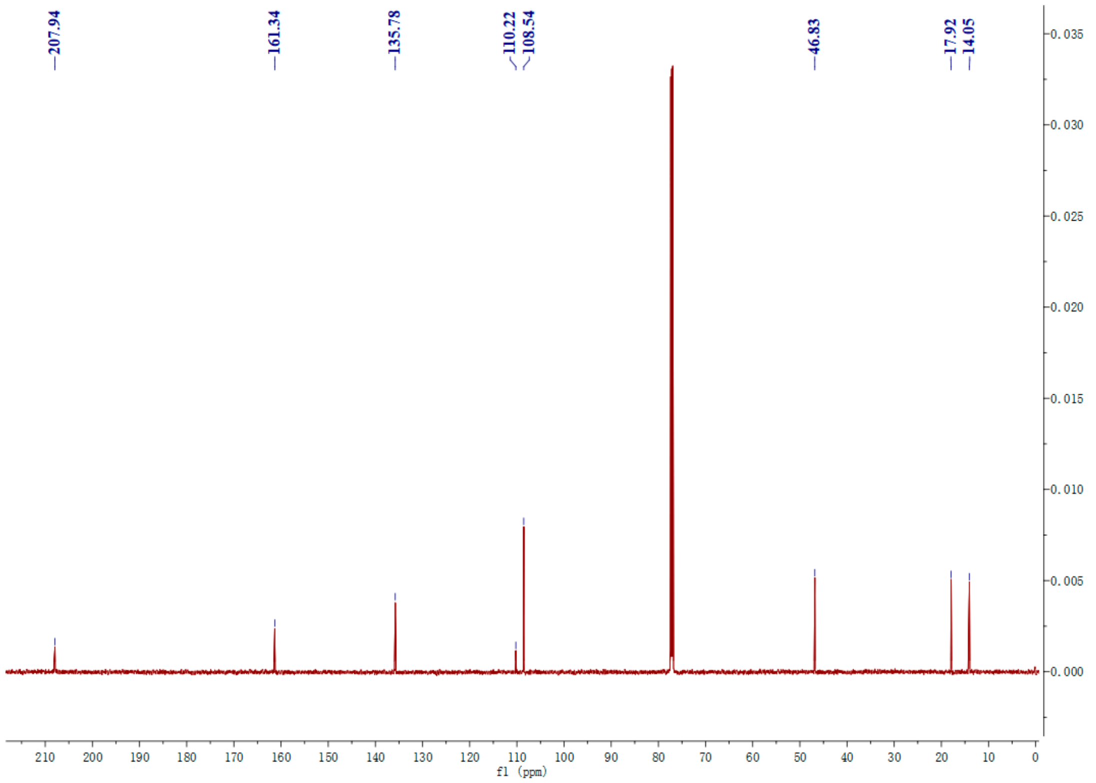

Figure S64. The ^{13}C NMR spectrum of **10** in $CDCl_3$
